# Supplementary material for: Time intervals and distances travelled for prehospital ambulance stroke care: data from the randomised-controlled ambulance-based Rapid Intervention with Glyceryl trinitrate in Hypertensive stroke Trial-2 (RIGHT-2)
Source: BMJ Open. 2022 Nov 21;12(11):e060211. doi: 10.1136/bmjopen-2021-060211 (PMC9680177; doi:10.1136/bmjopen-2021-060211)
Supplement: Supplementary data [file bmjopen-2021-060211supp001.pdf]

**SUPPLEMENTAL MATERIAL**

# Time intervals and distances travelled for pre-hospital ambulance stroke care: data from the Rapid Intervention with Glyceryl trinitrate in Hypertensive stroke Trial-2 (RIGHT-2)

## TABLE OF CONTENTS


| | Page |
|---|---|
| Title | 1 |
| Supplemental Background | 1 |
| Supplemental Methods | 1 |
| Supplemental Results | 2 |
| List of supplemental Tables and Figures | 5 |
| Supplemental Tables (start from) | 6 |
| Supplemental Figures (start from) | 14 |
| Supplemental References | 22 |


## SUPPLEMENTAL BACKGROUND

In the 2011 census of England and Wales, 43.7 million people lived in urban areas (defined as an area with greater than 10,000 inhabitants[1]) with 9.3 million people (17.6%) living in rural areas. Conversely, rural areas cover 85% of the land area of England and Wales. The proportion of urban and rural populations covered varied between ambulance service with rural ranging from 0.2% (LAS) to 32.8% (WAS).

## SUPPLEMENTAL METHODS

### RIGHT-2 trial

Patients were excluded if they had any of the following: resided in a nursing home, hypoglycaemia (<2.5mmol/l), evidence of seizure at presentation, known to have a terminal illness, taken sildenafil (or equivalent) within 24 hours, or previously been enrolled into RIGHT-2.



Dixon M, *et al. BMJ Open* 2022; 12:e060211. doi: 10.1136/bmjopen-2021-060211

Patients were randomised (1:1) to receive either GTN patches or sham patches. A randomisation sequence was generated by the trial programmer at the Nottingham Stroke Trials Unit using random-permuted fixed-size blocks stratified by ambulance station. Identical looking numbered treatment packs were sent in blocks (four treatment packs per block) to each ambulance station. Trial-trained paramedics only carry one treatment pack at any time.

Participants were taken to hospitals with an acute stroke service - these joined the trial depending on capacity and feasibility to receive enrolled patients and deliver the trial protocol.[2] Information on participants was entered into the main trial database by hospital-based research staff, including the data collected in the ambulance (baseline, during treatment) and in hospital (admission, day 2, discharge or death, final diagnosis).

The trial was funded by the British Heart Foundation, sponsored by the University of Nottingham, had Health Research Authority ethics committee approval, and was eligible for National Institute of Health Research Clinical Research Network support.

**Data sharing**

Individual participant data will be shared with the Blood pressure in Acute Stroke Collaboration (BASC) and Virtual International Stroke Trials Archive (VISTA). From 1 Jan 2022, the Chief Investigator (with approval from the Trial Steering Committee as necessary) will consider other requests to share individual participant data via email at: right-2@nottingham.ac.uk. We will require a protocol detailing hypothesis, aims, analyses, and intended tables and figures. Where possible, we will perform the analyses; alternatively, de-identified data and a data dictionary will be supplied for the necessary variables for remote analysis. Any sharing will be subject to a signed data access agreement. Ultimately, the entire trial dataset will be published.

## SUPPLEMENTAL RESULTS

Although all 10 stroke-receiving hospitals participated in the trial in the EMAS area, not all hospitals in the other seven ambulance services took part in the trial (range 1-13; Table 1).

**Time Intervals**

The extended time from the emergency call to dispatch of 5 minutes for SWAS was due, in part, to a service-wide evaluation of a new ambulance dispatch model (Ambulance Response Programme). The extended time from dispatch to arrival at scene of 12 minutes for WAS is likely to reflect, in part, the high proportion of rural-based patients in Wales and the WAS Clinical Response Model which was introduced during the trial and differs to England.

The time on scene in EMAS was comparable to a cohort of non-RIGHT-2 stroke patients despite the addition of consent, randomisation and treatment activities (34 [26, 44] vs 32 [23, 41] minutes, p=0.12) (Supplemental Table VI).

RIGHT-2 treatment was administered to 910 (80%) patients prior to departing scene and 239 (20%) patients en-route to hospital highlighting the speed and simplicity of the intervention.

**Multiple comparison testing for Supplemental Table IV:**

Significant differences existed between ambulance service with regards to timings:

- Emergency call to dispatch: LAS differs from EEAS p<0.001, EMAS p<0.001, SCAS p=0.011, SWAS p=0.008, WAS p<0.001 WMAS p=0.015 and YAS p=0.002. SWAS differs from EEAS p<0.001, EMAS p<0.001, SCAS p=0.001, WAS p<0.001 and YAS p<0.001
- Dispatch to arrival at scene: LAS differs from EEAS p=0.022, YAS p<0.001, SWAS p<0.001 and WAS p<0.001. EMAS differs from YAS p=0.024, SWAS p<0.001 and WAS p=0.003. EEAS differs from WAS p=0.036
- Dispatch to arrival of RIGHT-2 Paramedic: LAS differs from SWAS p=0.012, EEAS P=0.019, YAS p=0.002 and WAS p=0.003. EMAS differs from YAS p=0.037 and WAS p=0.030
- Time of arrival on scene to randomisation: WMAS differs from EEAS p=0.011, WAS p=0.015 and LAS p=0.001. EMAS differs from SWAS p<0.001, EEAS p<0.001, WAS p<0.001 and LAS p<0.001. YAS differs from EEAS p=0.003, WAS p=0.013 and LAS p<0.001
- Onset to randomisation: EMAS differs from SWAS p=0.021 and LAS p<0.001



Dixon M, *et al. BMJ Open* 2022; 12:e060211. doi: 10.1136/bmjopen-2021-060211

- Consent to randomisation: LAS differs from WMAS p=0.030, SWAS p<0.001, EMAS p<0.001, YAS p<0.001, WAS p<0.001 and EEAS p<0.001. SWAS differs from EEAS p=0.003
- Time on scene: EEAS differs from LAS p<0.001, YAS p=0.006, EMAS p=0.046. LAS differs from WAS p=0.001
- Depart scene to arrival at hospital: WMAS differs from EMAS p=0.018, EEAS p<0.001, WAS p<0.001 and SWAS p<0.001. YAS differs from EEAS p=0.003, WAS p=0.002 and SWAS p<0.001. SWAS differs from LAS p<0.001 and EMAS p<0.001
- Onset to hospital: EMAS differs from SWAS p=0.014 and WAS p=0.043. LAS differs from EEAS p<0.001, EMAS p<0.001, SWAS p<0.001, WAS p<0.001, WMAS p<0.001 and YAS p<0.001
- Randomisation to treatment: LAS differs from EEAS p<0.001, EMAS p<0.001, SWAS p<0.001, SCAS p<0.001, WAS p<0.001, WMAS p<0.001 and YAS p<0.001
- Randomisation to hospital: SWAS differs from YAS p<0.001 and WMAS p=0.023

**Distances**

Although it was not possible to collect information on where the ambulance was when dispatched to the scene for seven ambulance services, this information was available for EMAS; the median linear distance from point of dispatch to the stroke scene was 7.3 [3.5, 12.0] km.

**LIST OF SUPPLEMENTAL TABLES AND FIGURES**


| Supplemental Tables | |
|---|---|
| I | Comparison of timings and conveyance distance by urban versus rural geography |
| II | Demographic and baseline characteristics of patients enrolled into RIGHT-2 |
| III | Univariate correlations between baseline characteristics for age, sex, heart rate, systolic blood pressure and time from onset of symptoms to randomisation |
| IV | Timings (in minutes) by ambulance service |
| V | Timings: Onset of symptoms to emergency call |
| VI | Comparison of EMAS stroke patients in RIGHT-2 versus non-RIGHT-2 |
| VII | Timings: Symptom onset to arrival at hospital |
| VIII | Conveyance distances |

| Supplemental Figures | |
|---|---|
| I | Distribution of Randomisation by Ambulance Service |
| A | EMAS |
| B | EEAS |
| C | SCAS |
| D | SWAS |
| E | WAS |
| F | WMAS |
| G | YAS |




Dixon M, *et al. BMJ Open* 2022; 12:e060211. doi: 10.1136/bmjopen-2021-060211

**SUPPLEMENTAL TABLE I**. Comparison of timings and conveyance distance by urban versus rural geography. Data are median [25, 75 centile]; comparison by Mann-Whitney U test with difference (95% confidence intervals)

| | Urban | Rural | Difference (95% CI) | p |
|---|---|---|---|---|
| % rural | 13.3 | 30.0 | - | - |
| *Timing (minutes)* | | | | |
| Onset to emergency call | 23 [8, 70] | 17 [5, 58] | 19 (17, 23) | 0.003 |
| emergency call to dispatch | 3 [1, 7] | 3 [1, 8] | 3 (3, 3) | 0.17 |
| Onset to R2 scene | 47 [24, 95] | 43 [22, 84] | 45 (42, 48) | 0.06 |
| Onset to randomisation | 73 [47, 121] | 70 [45, 114] | 71 (68, 75) | 0.10 |
| Arrive to depart scene | 29 [22, 35] | 32 [24, 41] | 31 (30, 31) | <0.001 |
| Depart scene to hospital | 12 [9, 18] | 17 [10, 25] | 15 (14, 16) | <0.001 |
| *Distance (km)* | | | | |
| Conveyance | 5.7 [3.3, 10.6] | 11.7 [5.0, 20.4] | 9.5 (8.6, 10.3) | <0.001 |

Urban: LAS, WMAS, YAS, SCAS; rural: EMAS, EEAS, SWAS, WAS

**SUPPLEMENTAL TABLE II. Demographic and baseline characteristics of patients enrolled into RIGHT-2**

Data are number (%), median [interquartile range] or mean (standard deviation). Comparisons between ambulance services using Chi-square test, Kruskal-Wallis or one-way ANOVA.

| Characteristic | E&W | EEAS | EMAS | LAS | SCAS | SWAS | WAS | WMAS | YAS | p |
|---|---|---|---|---|---|---|---|---|---|---|
| Patients (%) | 1149 | 178 (15.5) | 218 (19.0) | 202 (17.6) | 7 (0.6) | 265 (23.1) | 89 (7.7) | 37 (3.2) | 153 (13.3) | - |
| Consent from (%) | | | | | | | | | | 0.58 |
| Patient | 603 (52.5) | 96 (52.2) | 111 (50.9) | 117 (57.9) | 4 (57.1) | 131 (49.4) | 48 (53.9) | 18 (48.6) | 81 (52.9) | |
| Relative/Friend | 431 (37.5) | 73 (41.0) | 90 (41.3) | 70 (34.7) | 1 (14.3) | 102 (38.5) | 23 (25.8) | 15 (40.5) | 57 (37.3) | |
| Paramedic | 115 (10.0) | 12 (6.7) | 17 (7.8) | 15 (7.4) | 2 (28.6) | 32 (12.1) | 18 (20.2) | 4 (10.8) | 15 (9.8) | |
| Age (years) | 72.5 (14.6) | 73.4 (13.4) | 71.6 (13.9) | 70.4 (16.3) | 76.6 (15.4) | 74.4 (14.7) | 74.7 (14.8) | 70.2 (17.2) | 71.1 (13.7) | 0.34 |
| <80 (%) | 714 (62.1) | 114 (64.0) | 144 (66.1) | 131 (64.9) | 3 (42.9) | 150 (56.6) | 48 (53.9) | 22 (59.5) | 102 (66.7) | |
| >=80 (%) | 435 (37.9) | 64 (36.0) | 74 (33.9) | 71 (35.1) | 4 (57.1) | 115 (43.4) | 41 (46.1) | 15 (40.2) | 51 (33.3) | |
| Female (%) | 555 (48.3) | 83 (46.6) | 98 (45.0) | 106 (52.5) | 1 (14.3) | 134 (50.6) | 44 (49.4) | 16 (43.2) | 73 (47.7) | |
| FAST (/3) | 2.6 (0.51) | 2.5 (0.53) | 2.6 (0.50) | 2.5 (0.50) | 2.1 (0.38) | 2.6 (0.51) | 2.6 (0.21) | 2.6 (0.55) | 2.7 (0.50) | 0.31 |
| 3 N(%) | 692 (60.1) | 98 (55.1) | 137 (62.8) | 107 (53.0) | 1 (14.3) | 166 (62.9) | 56 (63.6) | 22 (59.5) | 103 (67.3) | |
| 2 N(%) | 446 (8.8) | 77 (43.3) | 80 (36.7) | 95 (47.0) | 9 (85.7) | 95 (36.0) | 31 (35.2) | 14 (37.8) | 48 (31.14) | |
| 1 N(%) † | 11 (1.0) | 3 (1.7) | 1 (0.5) | 0 (0.0) | 0 (0.0) | 3 (1.1) | 1 (1.1) | 1 (2.7) | 2 (1.3) | |
| GCS (/15) | 13.9 (1.7) | 13.6 (1.8) | 14.0 (1.7) | 13.9 (1.7) | 13.7 (1.8) | 14.0 (1.6) | 13.9 (1.7) | 13.9 (1.7) | 13.9 (1.7) | 0.50 |
| <14 | 302 (26) | 57 (32.0) | 52 (23.9) | 49 (24.3) | 2 (28.6) | 66 (24.9) | 26 (29.2) | 11 (29.7) | 39 (25.5) | 0.65 |
| Haemodynamics | | | | | | | | | | |
| SBP (mmHg) | 162.1 (25.1) | 159.38 (25.4) | 164.4 (27.4) | 163.9 (26.2) | 166.4 (27.7) | 161.3 (25.0) | 162.8 (26.3) | 167.7 (28.4) | 162.2 (26.8) | 0.49 |
| DBP (mmHg) | 91.6 (17.9) | 90. (21.1) | 93.7 (19.4) | 92.1 (20.4) | 93.0 (12.4) | 91.2 (17.9) | 92.5 (17.7) | 90.5 (24.4) | 91.6 (18.6) | 0.68 |
| HR (bpm) | 82.2 (18.6) | 81.8 (18.3) | 83.3 (18.9) | 81.9 (20.1) | 79.1 (18.0) | 82.6 (22.2) | 82.4 (18.3) | 91.9 (26.7) | 82.2 (18.1) | 0.21 |
| Temperature | 36.4 (0.6) | 36.6 (0.6) | 36.5 (0.6) | 36.5 (0.6) | 36.5 (0.6) | 36.6 (0.6) | 36.5 (0.7) | 36.6 (0.6) | 36.3 (0.7) | 0.012 |
| Glucose (mmol/l) | 7.5 (3.3) | 7.5 (3.0) | 7.1 (2.6) | 7.8 (4.2) | 5.8 (1.0) | 8.1 (3.3) | 7.0 (2.3) | 6.9 (3.0) | 7.7 (4.0) | 0.18 |
| Diagnosis (%) | | | | | | | | | | 0.05 |
| ICH | 145 (12.6) | 23 (12.9) | 25 (11.5) | 19 (9.4) | 1 (14.3) | 39 (14.7) | 9 (10.1) | 4 (10.8) | 25 (16.3) | |
| Ischaemic | 597 (52.0) | 89 (50.0) | 122 (56.0) | 89 (44.1) | 5 (71.4) | 141 (53.2) | 50 (56.2) | 20 (54.1) | 81 (52.9) | |
| TIA | 109 (9.5) | 14 (7.9) | 21 (9.6) | 21 (10.4) | 0 (0.0) | 28 (10.6) | 12 (13.5) | 2 (5.4) | 11 (7.2) | |
| Mimic | 298 (25.9) | 52 (29.2) | 50 (22.9) | 73 (36.1) | 1 (14.3) | 57 (21.5) | 18 (20.2) | 11 (29.7) | 36 (23.5) | |

† Protocol violation

SBP: systolic blood pressure; DBP: diastolic blood pressure; HR: heart rate; bpm: beats per minute; E&W: England & Wales; EEAS – East of England Ambulance Service NHS Trust; EMAS – East Midlands Ambulance Service NHS Trust; FAST: Face arm speech test; GCS: Glasgow coma scale; ICH: intracerebral haemorrhage; LAS – London Ambulance Service; mRS: modified Rankin Scale (premorbid); OTR: onset to randomisation; SCAS – South Central Ambulance Service NHS Foundation Trust; SWAS – South Western Ambulance Service NHS Foundation Trust; TIA: transient ischaemic attack; WAS – Welsh Ambulance Service NHS Trust; YAS – Yorkshire Ambulance Service NHS Trust.

Multiple comparison procedure: Temperature - YAS differs from SWAS (p=0.002) and EEAS (p=0.002)



Dixon M, *et al. BMJ Open* 2022; 12:e060211. doi: 10.1136/bmjopen-2021-060211

**SUPPLEMENTAL TABLE III.** Univariate correlations between baseline characteristics for age, sex, heart rate, systolic blood pressure and time from onset of symptoms to randomisation. Data are Spearman or point-biserial correlation coefficient (p-value).

| | Sex | HR | SBP | Glucose | OTR | FAST | GCS |
|---|---|---|---|---|---|---|---|
| Age | -0.123 (<0.001) | -0.111 (<0.001) | 0.076 (0.010) | 0.140 (<0.001) | 0.072 (0.15) | -0.004 (0.90) | -0.158 (<0.001) |
| Sex | | -0.094 (0.001) | -0.069 (0.019) | -0.009 (0.76) | 0.037 (0.21) | 0.002 (0.95) | 0.009 (0.76) |
| HR | | | 0.119 (<0.001) | 0.069 (0.019) | -0.012 (0.67) | 0.007 (0.80) | 0.006 (0.85) |
| SBP | | | | 0.26 (1.00) | 0.005 (0.86) | -0.075 (0.011) | 0.044 (0.14) |
| Glucose | | | | | 0.023 (0.44) | 0.059 (0.45) | -0.77 (0.009) |
| OTR | | | | | | -0.137 (<0.001) | 0.102 (0.001) |
| FAST | | | | | | | -0.157 (<0.001) |

HR: heart rate; OTR: onset to randomisation; SBP: systolic blood pressure



Dixon M, *et al. BMJ Open* 2022; 12:e060211. doi: 10.1136/bmjopen-2021-060211

**SUPPLEMENTAL TABLE IV. Timings (in minutes) by ambulance service**

Data are median [interquartile range] (minimum-maximum). Comparisons by Kruskal-Wallis and multiple comparison procedures (between groups) using Dunn's test with Bonferroni correction.

| | | E&W | EEAS | EMAS | LAS | SCAS | SWAS | WAS | WMAS | YAS | p |
|---|---|---|---|---|---|---|---|---|---|---|---|
| Patients (N) | | 1149 | 178 | 218 | 202 | 7 | 265 | 89 | 37 | 153 | - |
| Onset † | emergency Call | 19 [5, 64] (-216, 920) | 18 [6, 69] (-58, 183) | 15 [4, 57] (-89, 661) | 25 [7, 76] (7, 76) | 24 [11, 48] (1, 776) | 18 [4, 55] (-216, 835) | 14 [4, 46] (-45, 199) | 27 [7, 75] (-25, 206) | 20 [8, 65] (-10, 514) | 0.36 |
| emergency Call | Dispatch | 3 [1, 7] (0, 158) | 1 [0, 4] (0, 115) | 2 [1, 4] (0, 73) | 4 [2, 8] (0, 78) | 0 [0, 1] (0, 3) | 5 [3, 14] (0, 131) | 1 [0, 11] (0, 158) | 2 [1, 3] (0,17) | 2 [1, 7] (0, 55) | <0.001 |
| Onset † | Arrive Scene (R1) | 40 [21, 84] (-124, 928) | 39 [19, 85] (-40, 205) | 30 [15, 74] (-75, 691) | 45 [19, 95] (-21, 928) | 35 [33, 56] (11, 792) | 45 [25, 79] (-124, 838) | 28 [25, 85] (-19, 207) | 39 [20, 95] (-14, 214) | 40 [24, 84] (5, 523) | 0.04 |
| Onset † | Arrive Scene (R2) | 44 [23, 86] (-124, 928) | 43 [21, 88] (-40, 206) | 35 [19, 80] (-75, 691) | 50 [22, 97] (-75, 928) | 35 [33, 56] (11, 792) | 58 [25, 83] (-124, 838) | 40 [25, 87] (-17, 207) | 39 [20, 95] (-14, 214) | 42 [25, 92] (5, 423) | 0.23 |
| Dispatch | Arrive Scene (R1) | 8 [5, 13] (-31, 61) | 8 [4, 14] (-26, 61) | 7 [4, 12] (-31, 36) | 6 [4, 9] (0, 28) | 11 [7, 15] (6, 22) | 10 [5, 17] (-18, 48) | 12 [6, 17] (-9, 40) | 9 [5, 13] (0, 39) | 9 [6, 23] (-31, 39) | <0.001 |
| Dispatch | RIGHT-2 paramedic | 10 [6, 16] (0, 75) | 10 [6, 19] (1, 61) | 8 [5, 15] (0, 53) | 8 [5, 13] (0, 65) | 11 [7, 15] (6, 22) | 11 [6, 18] (0, 75) | 12 [7, 18] (0, 72) | 9 [6, 14] (0, 39) | 9 [6, 13] (-31, 39) | <0.001 |
| Onset | Randomisation | 71 [45, 116] (4, 942) | 73 [47, 120] (11, 250) | 59 [35, 100] (4, 720) | 77 [51, 124] (15, 942) | 53 [45, 65] (19, 811) | 75 [49, 107] (6, 850) | 75 [48, 123] (11, 395) | 60 [32, 115] (17, 225) | 70 [45, 118] (15, 535) | <0.001 |
| Onset | Treatment | 72 [48, 117] (4, 942] | 73 [49, 73] (11, 251) | 60 [37, 104] (4, 720) | 77 [52, 124] (15, 942) | 59 [50, 75] (19, 816) | 78 [50, 109] (6, 874) | 77 [48, 120] (11, 230) | 65 [35, 118] (22, 229) | 71 [48, 119] (22, 535) | 0.005 |
| RIGHT-2 Paramedic arrival † | Consent | 19 [12, 29] (-17, 128) | 20 [15, 28] (-1, 91) | 15 [9, 22] (-5, 76) | 25 [18, 33] (1, 73) | 8 [5, 18] (2, 29) | 20 [13, 30] (-10, 128) | 23 [14, 32] (0, 87) | 16 [9, 21] (-9, 46) | 17 [10, 25] (-17, 65) | <0.001 |
| RIGHT-2 Paramedic arrival † | Randomisation | 22 [15, 31] (-34, 130) | 23 [18, 32] (1, 95) | 17 [11, 27] (-34, 80) | 26 [19, 33] (1, 73) | 11 [8, 19] (5, 30) | 22 [16, 32] (-10, 130) | 25 [18, 35] (0, 87) | 17 [12, 24] (-8, 46) | 19 [12, 27] (-13, 71) | <0.001 |
| Consent | Randomisation | 1 [0, 4] (0, 30) | 2 [0, 4] (0, 30) | 1 [0, 4] (0, 24) | 0 [0, 0] (0, 18) | 1 [0, 5] (0, 9) | 0 [0, 4] (0, 20) | 2 [0, 5] (0, 29) | 1 [0, 3] (0, 10) | 1 [0, 4] (0, 23) | <0.001 |
| Arrive Scene | Depart scene | 33 [26, 46] (0, 224) | 38 [29, 49] (15, 224) | 33 [25, 44] (10, 94) | 31 [25, 38] (8, 72) | 29 [23, 40] (10, 41) | 43 [34, 87] (0, 162) | 38 [28, 51] (15, 114) | 31 [25, 38] (16, 46) | 32 [24, 43] (0, 84) | <0.001 |
| Onset | Hospital | 97 [71, 141] (26, 953) | 103 [73, 149] (29, 295) | 87 [65, 129] (26, 748) | 95 (68, 144] (32, 953) | 90 [81, 115] (55, 841) | 106 [77, 141] (32, 889) | 109 [78, 153] (39, 430) | 86 [58, 150] (31, 256) | 92 [70, 131] (38, 545) | 0.008 |
| Randomisation | Treatment | 0 [0, 2] (0, 57) | 0 [0, 3] (0, 20) | 0 [0, 2] (0, 57) | 0 [0, 0] (0, 9) | 5 [0, 8] (0, 9) | 1 [0,4] (0, 27) | 0 [0, 2] (0, 19) | 1 [0, 4] (0, 24) | 0 [0, 2] (0, 22) | <0.001 |
| Randomisation | Hospital | 24 [16, 34] (-13, 229) | 25 [18, 33] (2, 229) | 26 [17, 35] (-13, 92) | 14 [10, 21] | 35 [30, 36] (25, | 29 [21, 39] (4, 85) | 30 [19, 42] (-1, | 22 [19, 28] (6, 48) | 23 [16, 29 (-3, 86)] | <0.001 |

| | | | | | | | | | | | |
|---|---|---|---|---|---|---|---|---|---|---|---|
| | | | | | (0, 52) | 62) | | 92) | | | |
| Depart scene | Hospital | 15 [10, 23] (0, 98) | 17 [10, 23] (1, 47) | 14 [9, 23] (0, 50) | 13 [10, 19] (2, 49) | 24 [14, 25] (4, 33) | 20 [12, 29] (0, 98) | 16 [10, 28] (0, 56) | 9 [7, 13] (-3, 27) | 12 [8, 17] (0, 45) | <0.001 |

† Negative times: paramedic already at scene

E&W: England & Wales; EEAS – East of England Ambulance Service NHS Trust; EMAS – East Midlands Ambulance Service NHS Trust; LAS – London Ambulance Service; R1: First resource; R2: RIGHT-2 trained paramedic (if not on first resource) – two ambulance services permitted single responder paramedics to participate, otherwise R2 trained paramedics arrive on double-crewed ambulances; SCAS – South Central Ambulance Service NHS Foundation Trust; SWAS – South Western Ambulance Service NHS Foundation Trust; WAS – Welsh Ambulance Service NHS Trust; YAS – Yorkshire Ambulance Service NHS Trust.

Results of multiple comparison testing are given in the Supplemental material.



Dixon M, *et al. BMJ Open* 2022; 12:e060211. doi: 10.1136/bmjopen-2021-060211

**SUPPLEMENTAL TABLE V.** Timings: Onset of symptoms to emergency call. Data are N (%), median [25, 75 centile]; comparison by Kruskal-Wallis test.

| | E&W | EEAS | EMAS | LAS | SCAS | SWAS | WAS | WMAS | YAS | p |
|---|---|---|---|---|---|---|---|---|---|---|
| Minutes<br>N (%)<br>Median [25, 75 centile] | 1149<br>19 [5, 64] | 178 (15.5)<br>18 [6, 69] | 218 (19.0)<br>15 [4, 57] | 202 (17.6)<br>25 [7, 76] | 7 (0.6)<br>24 [11, 48] | 265 (23.1)<br>18 [4, 55] | 89 (7.7)<br>14 [4, 46] | 37 (3.2)<br>27 [7, 75] | 153 (13.3)<br>20 [8, 65] | 0.36 |
| N (%) | | | | | | | | | | 0.012 |
| <10 | 430 (37.4) | 67 (37.6) | 94 (43.1) | 68 (33.7) | 1 (14.3) | 102 (38.5) | 40 (44.9) | 12 (32.4) | 46 (30.1) | |
| 11-20 | 169 (14.7) | 26 (14.6) | 29 (13.3) | 27 (13.4) | 1 (14.3) | 39 )14.7) | 11 912.4) | 4 (10.8) | 32 (20.9) | |
| 21-30 | 84 (7.3) | 9 (5.1) | 18 (8.3) | 14 (6.9) | 2 (28.6) | 23 (8.7) | 3 (3.4) | 3 (8.1) | 12 (7.8) | |
| 31-60 | 164 (14.3) | 23 (12.9) | 25 (11.5) | 29 (14.4) | 2 (28.6) | 43 (16.2) | 16 (18.0) | 5 (13.5) | 21 (13.7) | |
| 61-240 | 290 (25.2) | 53 (29.8) | 50 (22.9) | 58 (28.7) | 0 (0.0) | 57 (21.5) | 18 (20.2) | 13 (35.1) | 41 (26.8) | |
| >240 † | 11 (1.0) | 0 (0.0) | 2 (0.9) | 6 (3.0) | 1 (14.3) | 1 (0.4) | 88 (98.9) | 0 (0.0) | 1 (0.7) | |

† >240 minutes is protocol violation, typically due to wake-up stroke or uncertainty of onset time.



Dixon M, *et al. BMJ Open* 2022; 12:e060211. doi: 10.1136/bmjopen-2021-060211

**SUPPLEMENTAL TABLE VI.** Comparison of EMAS stroke patients in RIGHT-2 versus non-RIGHT-2. Data are median of time (min) [25, 75 centile] or distance km [25, 75 centile]

| | RIGHT-2 | Non-RIGHT-2 | p |
|---|---|---|---|
| Patients (N) | 218 | 49 | |
| Time | | | |
| Symptom onset – emergency call | 15 [5, 57] | † | |
| emergency call – dispatch | 2 [1, 5] | 2 [1, 3] | 0.48 |
| emergency call – scene arrival | 10 [7, 16] | 12 [8, 18] | 0.17 |
| Time on scene | 34 [26, 44] | 32 [23, 41] | 0.12 |
| Scene arrival - hospital | | | |
| Scene departure – hospital | 14 [9, 22] | 17 [12, 25] | 0.18 |
| Symptom onset – hospital | 86 [65, 128] | † | |
| emergency call - hospital | 63 [48, 76] | 62 [49, 82] | 0.80 |
| Distance | | | |
| Dispatch - Scene | 7.3 [3.5, 12.0] | 9.6 [3.6, 16.7] | 0.23 |
| Scene - hospital | 10.0 [0.4, 64.7] | 15.9 [7.6, 24] | 0.011 |

† Symptom onset time not available



Dixon M, *et al. BMJ Open* 2022; 12:e060211. doi: 10.1136/bmjopen-2021-060211

**SUPPLEMENTAL TABLE VII.** Timings: Symptom onset to arrival at hospital (minutes). Data are N (%), median [25, 75 centile]; comparison by Kruskal-Wallis test.

| Minutes | E&W | EEAS | EMAS | LAS | SCAS | SWAS | WAS | WMAS | YAS | p |
|---|---|---|---|---|---|---|---|---|---|---|
| N (%) | 1149 | 178 | 218 | 202 | 7 | 265 | 89 | 37 | 153 | |
| Median [25, 75 centile] | 97 [71, 141] | 103 [73, 149] | 87 [65, 129] | 95 (68, 144] | 90 [81, 115] | 106 [77, 141] | 109 [78, 153] | 86 [58, 150] | 92 [70, 131] | 0.008 |
| N(%) | | | | | | | | | | 0.040 |
| <30 | 6 (0.5) | 1 (0.6) | 5 (2.3) | 0 (0.0) | 0 (0.0) | 0 (0.0) | 0 (0.0) | 0 (0.0) | 0 (0.0) | |
| 31-60 | 157 (13.7) | 15 (8.4) | 39 (17.9) | 37 (18.3) | 1 (14.3) | 24 (9.1) | 9 (10.1) | 10 (27.0) | 22 (14.4) | |
| 61-90 | 350 (30.5) | 61 (34.3) | 70 (32.1) | 54 (26.7) | 3 (42.9) | 75 (28.3) | 23 (25.8) | 10 (27.0) | 54 (35.3) | |
| 91-120 | 227 (19.8) | 29 (16.3) | 40 (18.3) | 39 (19.3) | 2 (28.6) | 62 (23.4) | 20 (22.5) | 5 (13.5) | 30 (19.6) | |
| 121-240 | 371 (32.3) | 68 (38.2) | 58 (26.6) | 64 (31.7) | 0 (0.0) | 96 (36.2) | 34 (38.2) | 11 (29.7) | 40 (26.1) | |
| >240 | 38 (3.3) | 4 (2.2) | 6 (2.8) | 8 (4.0) | 1 (14.3) | 8 (3.0) | 3 (3.4) | 1 (2.7) | 7 (4.6) | |

**SUPPLEMENTAL TABLE VIII.** Conveyance distances (kilometres). Data are median of distance (minimum-maximum). Comparison by Kruskal-Wallis test and multiple comparison procedure. One ambulance service was unable to provide location data.

| | E&W | EEAS | EMAS | SCAS | SWAS | WAS | WMAS | YAS | p |
|---|---|---|---|---|---|---|---|---|---|
| N (%) | 936 | 178 | 213 | 7 | 263 | 87 | 37 | 152 | |
| Median | 10.0 | 12.3 | 9.4 | 19.9 | 13.6 | 12.1 | 4.1 | 6.4 | <0.001 |
| (min, max) | [4.4, 18.4] | [4.5, 20.9] | [4.4, 19.1] | [2.7, 19.8] | [6.2, 20.1] | [4.7, 23.8] | [3.4, 10.2] | [3.3, 10.8] | |
| | (0.4, 64.7) | (0.6, 34.0) | (1.1, 59.9) | (1.9, 22.4) | (0.6, 51.3) | (0.6, 64.7) | (0.9, 28.8) | (0.4, 44.3) | |
| N (%) | | | | | | | | | <0.001 |
| <5 Km | 273 (29.2) | 49 (27.7) | 66 (31.0) | 2 (28.6) | 49 (18.6) | 20 (23.2) | 23 (62.2) | 64 (42.1) | |
| 5-10 km | 193 (20.6) | 25 (14.1) | 42 (19.7) | 0 (0.0) | 61 (23.2) | 18 (20.7) | 4 (10.8) | 43 (28.3) | |
| 10.1-15 km | 142 (15.2) | 27 (15.3) | 31 (14.6) | 1 (14.3) | 41 (15.6) | 13 (14.9) | 5 (13.5) | 24 (15.8) | |
| 15.1-20 km | 112 (12.0) | 22 (12.4) | 26 (12.2) | 1 (14.2) | 44 (16.7) | 10 (11.5) | 3 (8.1) | 6 (3.9) | |
| 20.1-25 km | 107 (11.4) | 34 (19.2) | 25 (11.7) | 3 (42.9) | 28 (10.6) | 10 (11.5) | 1 (2.7) | 6 (3.9) | |
| >=25 km | 109 (11.6) | 20 (11.3) | 23 (10.8) | 0 (0.0) | 40 (15.2) | 16 (18.4) | 1 (2.7) | 9 (5.9) | |

Multiple Comparison testing:
WMAS differs from EMAS p=0.016, EEAS p=0.001, SWAS p<0.001 and WAS p=0.001
YAS differs from EMAS p=0.002, EEAST p<0.001, SWAS p<0.001 and WAS p<0.001



Dixon M, *et al. BMJ Open* 2022; 12:e060211. doi: 10.1136/bmjopen-2021-060211

**SUPPLEMENTAL FIGURE I. Distribution of participants by Ambulance Service**

Map pins indicate location of participants recruited

**A) East Midlands Ambulance Service NHS Trust**

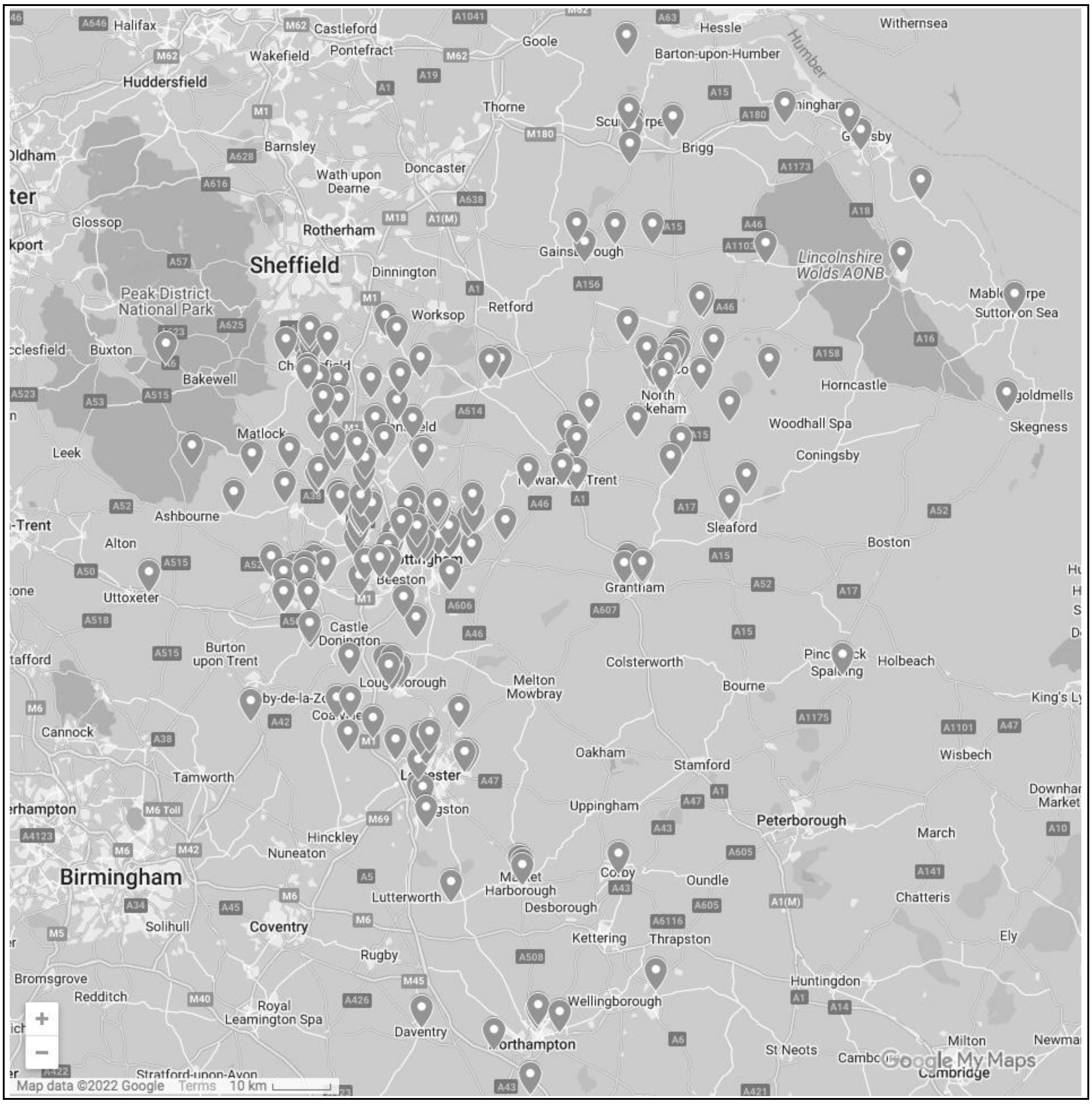

## B) East of England Ambulance Service NHS Trust

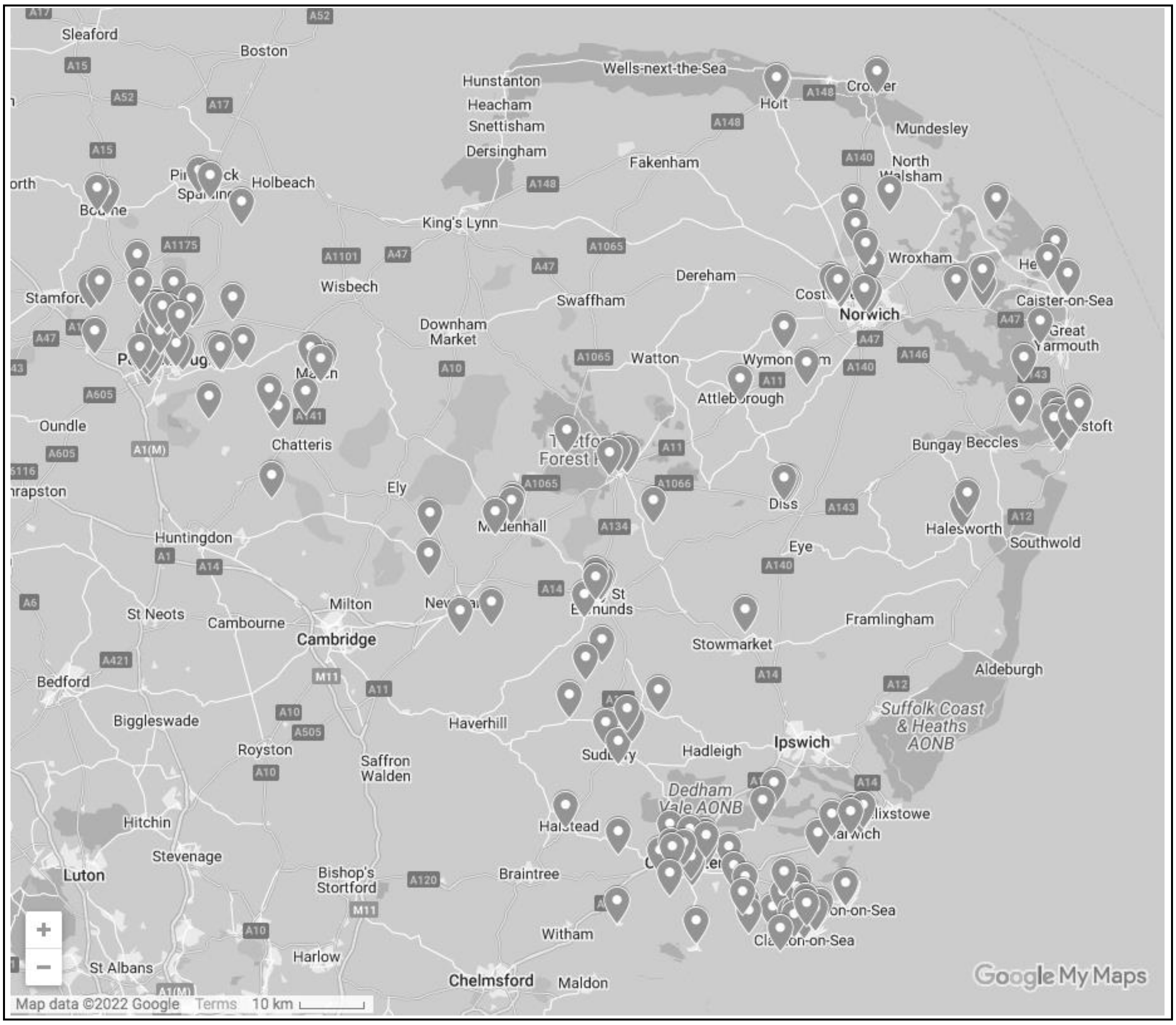

## C) South Central Ambulance Service NHS Foundation Trust

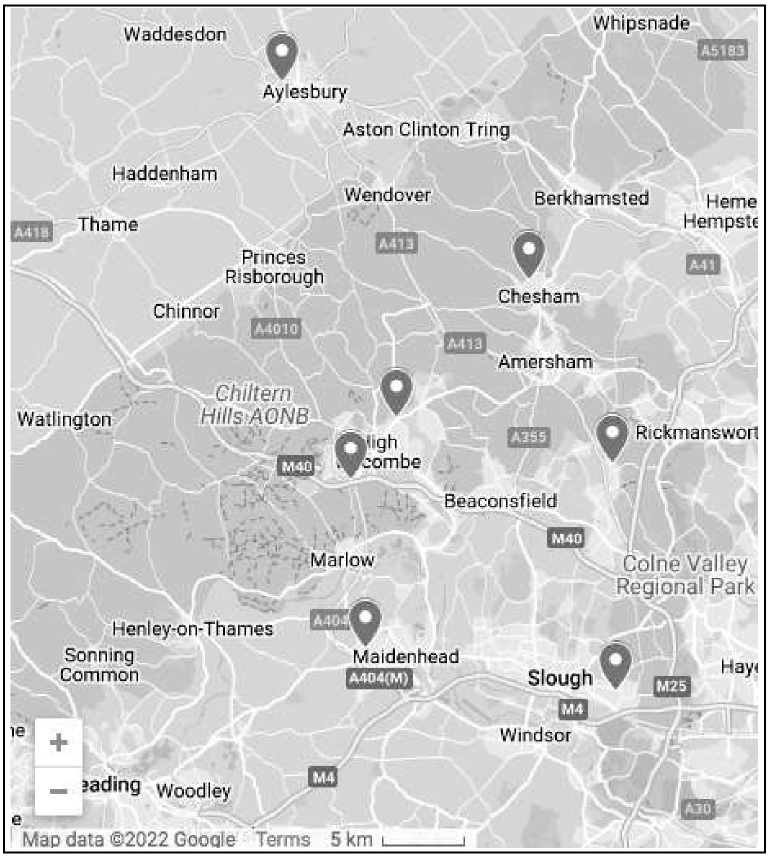

## D) South Western Ambulance Service NHS Foundation Trust

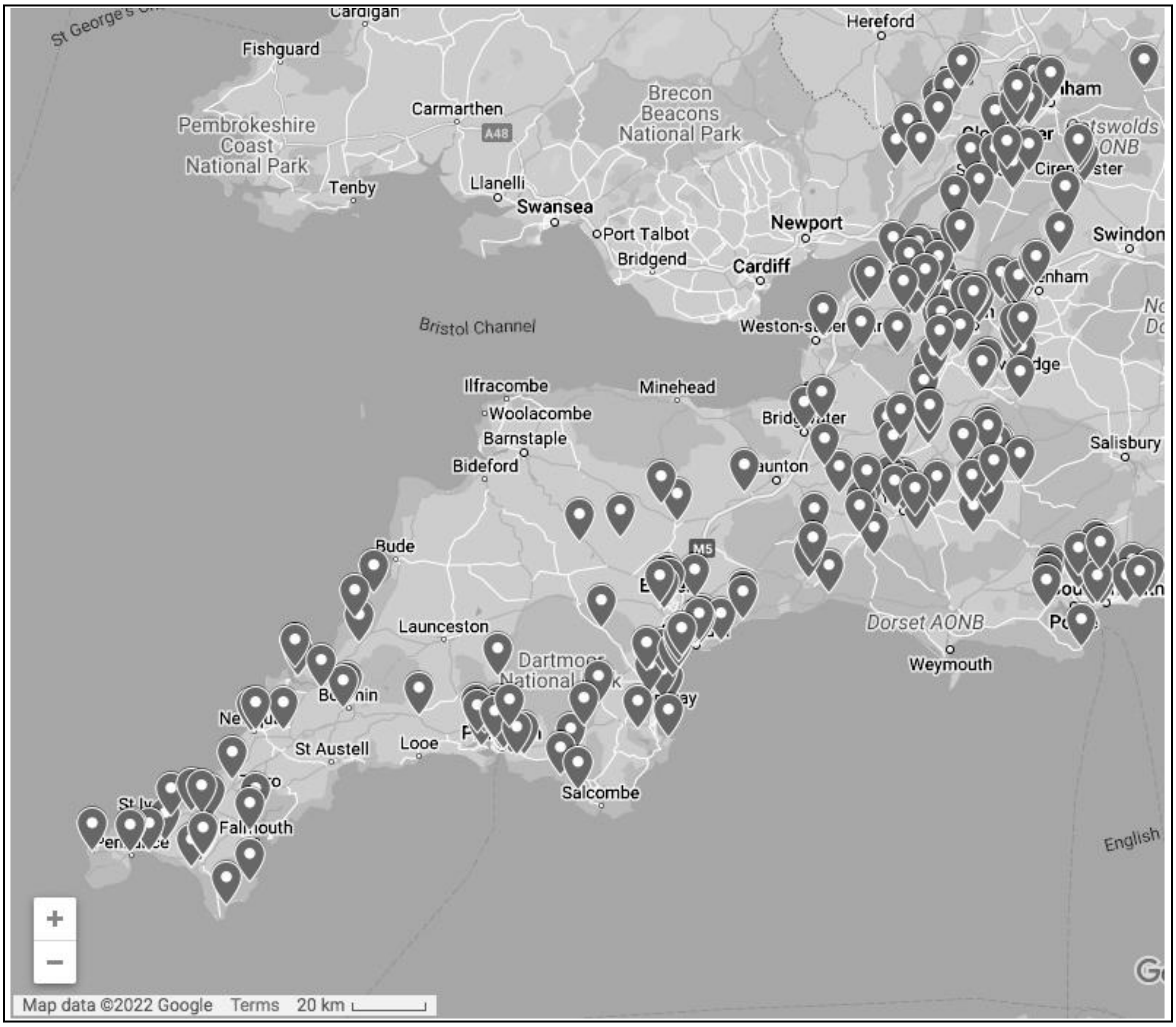

## E) Welsh Ambulance Service NHS Trust

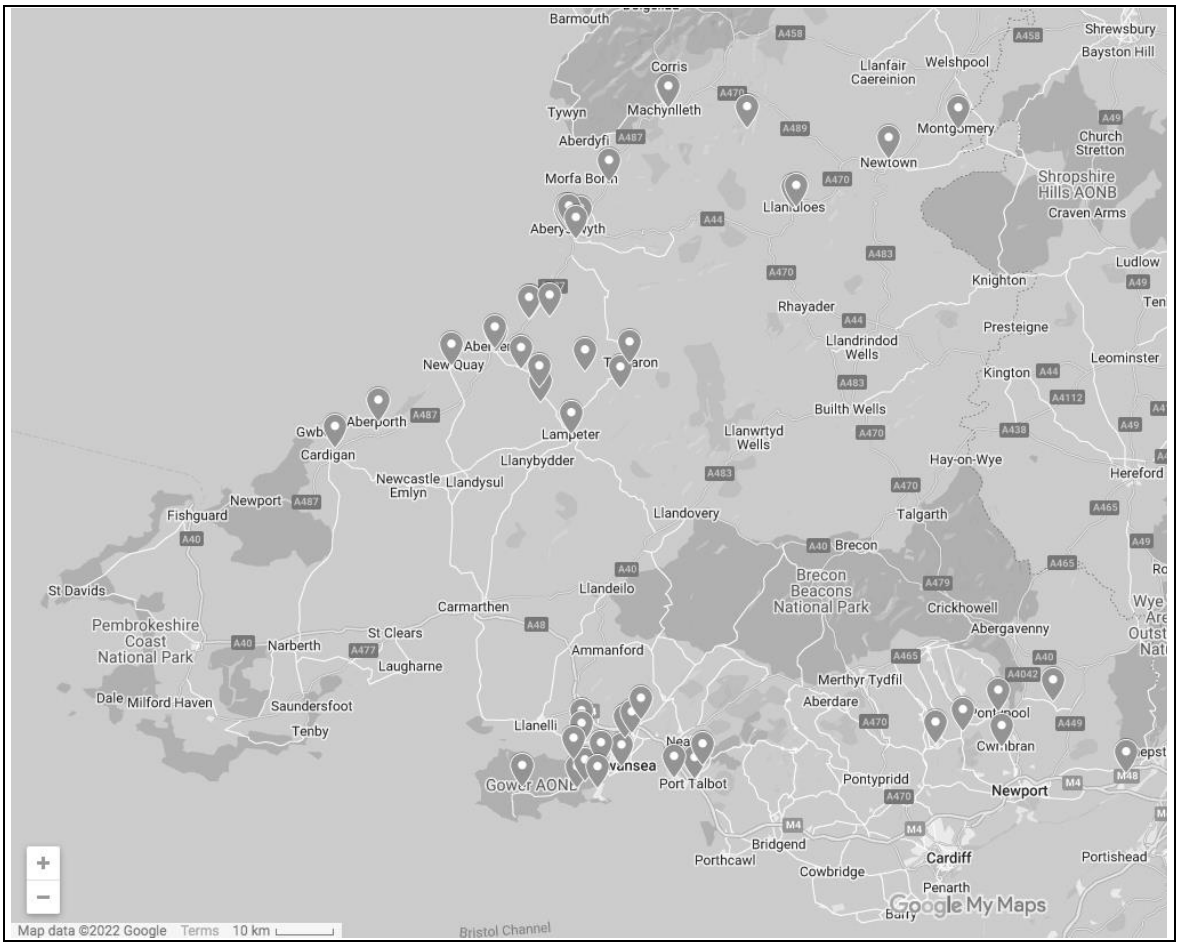

## F) West Midlands Ambulance Service University NHS Foundation Trust

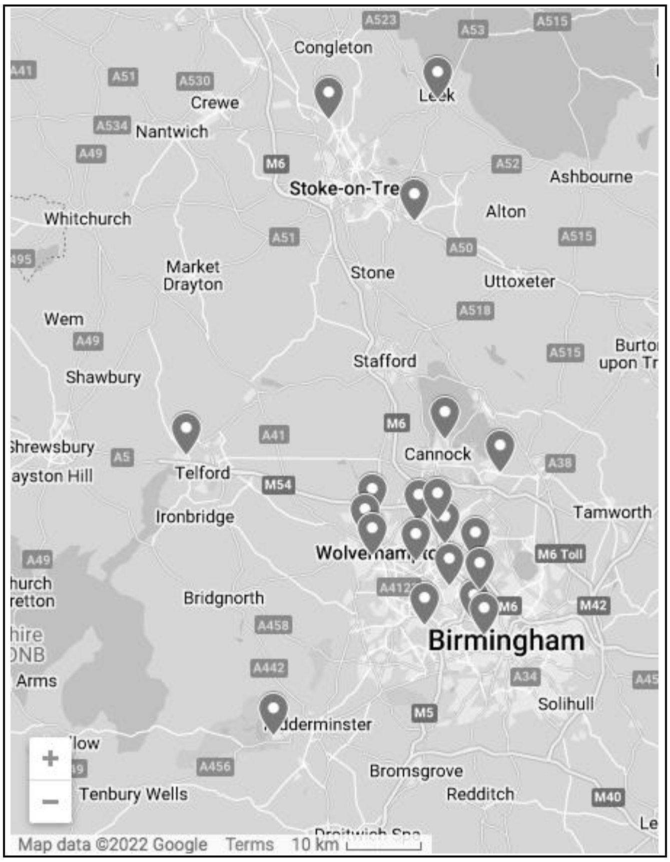

## G) Yorkshire Ambulance Service NHS Trust

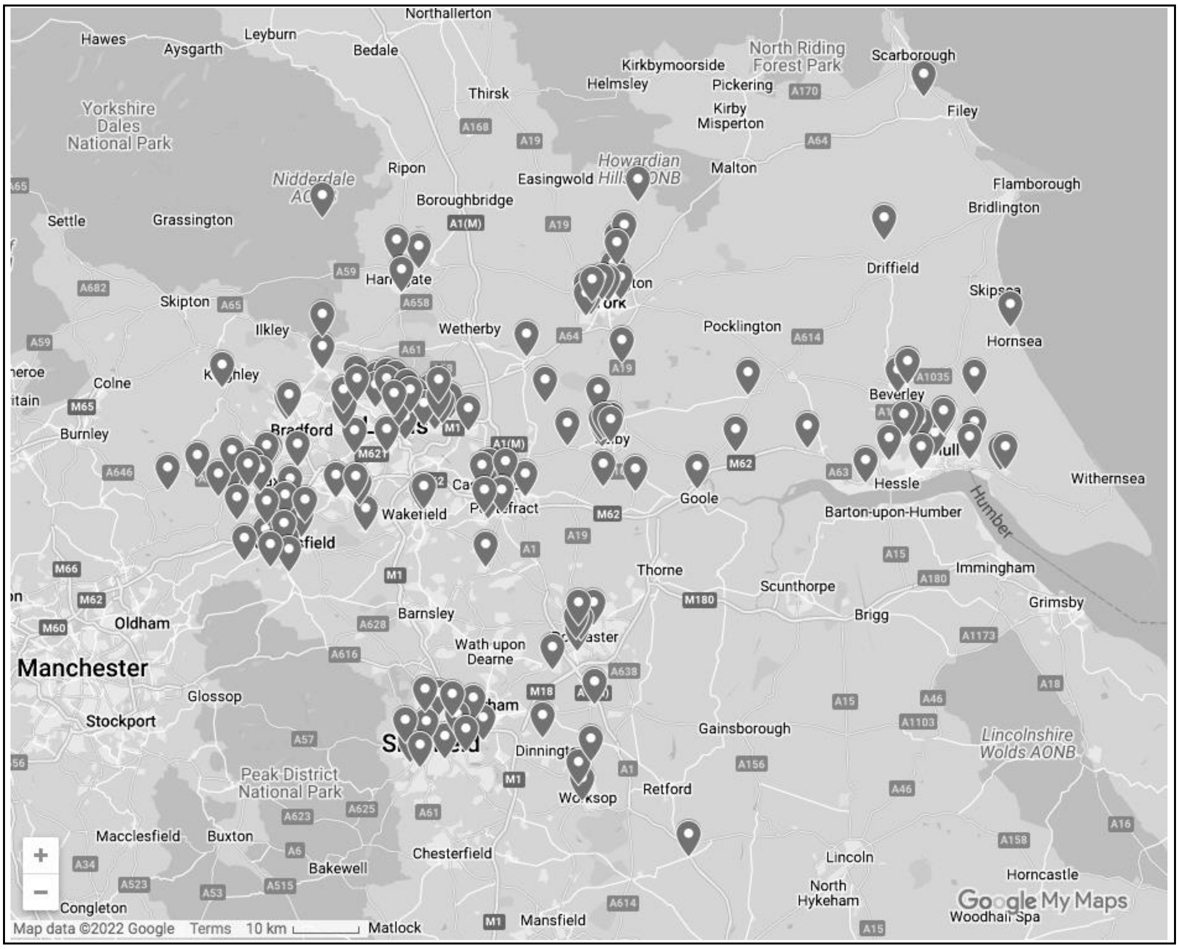

## SUPPLEMENTAL REFERENCES

1. Office for National Statistics. 2011 Census Analysis - Comparing Rural and Urban Areas of England and Wales: Office for National Statistics, 2013.
2. Appleton JP, Scutt P, Dixon M, et al. Ambulance-delivered transdermal glyceryl trinitrate versus sham for ultra-acute stroke: Rationale, design and protocol for the Rapid Intervention with Glyceryl trinitrate in Hypertensive stroke Trial-2 (RIGHT-2) trial (ISRCTN26986053). *Int J Stroke* 2017;0(0):1-16. doi: 10.1177/1747493017724627
