# Supplementary material for: Time intervals and distances travelled for prehospital ambulance stroke care: data from the randomised-controlled ambulance-based Rapid Intervention with Glyceryl trinitrate in Hypertensive stroke Trial-2 (RIGHT-2)
Source: BMJ Open. 2022 Nov 21;12(11):e060211. doi: 10.1136/bmjopen-2021-060211 (PMC9680177; doi:10.1136/bmjopen-2021-060211)
Supplement: Author's manuscript [file bmjopen-2021-060211.draft_revisions.pdf]

BMJ Open is committed to open peer review. As part of this commitment we make the peer review history of every article we publish publicly available.

When an article is published we post the peer reviewers' comments and the authors' responses online. We also post the versions of the paper that were used during peer review. These are the versions that the peer review comments apply to.

The versions of the paper that follow are the versions that were submitted during the peer review process. They are not the versions of record or the final published versions. They should not be cited or distributed as the published version of this manuscript.

BMJ Open is an open access journal and the full, final, typeset and author-corrected version of record of the manuscript is available on our site with no access controls, subscription charges or pay-per-view fees (http://bmjopen.bmj.com).

If you have any questions on BMJ Open's open peer review process please email info.bmjopen@bmj.com

BMJ Open

Time intervals and distances travelled for pre-hospital ambulance stroke care: data from the randomised-controlled ambulance-based Rapid Intervention with Glyceryl trinitrate in Hypertensive stroke Trial-2 (RIGHT-2)

Journal:	*BMJ Open*
Manuscript ID	bmjopen-2021-060211
Article Type:	Original research
Date Submitted by the Author:	06-Jan-2022

Complete List of Authors:	Dixon, Mark; University of Nottingham Faculty of Medicine and Health Sciences, Division of Mental Health and Clinical Neuroscience; East Midlands Ambulance Service NHS Trust,<br>Appleton, Jason; University Hospitals Birmingham NHS Foundation Trust<br>Scutt, Polly; University of Nottingham Faculty of Medicine and Health Sciences, Division of Mental Health and Clinical Neuroscience<br>Woodhouse, Lisa; University of Nottingham Faculty of Medicine and Health Sciences, Division of Mental Health and Clinical Neuroscience<br>Haywood, Lee; University of Nottingham Faculty of Medicine and Health Sciences, Division of Mental Health and Clinical Neuroscience<br>Havard, Diane; University of Nottingham Faculty of Medicine and Health Sciences, Division of Mental Health and Clinical Neuroscience<br>Williams, Julia; University of Hertfordshire, Division of Paramedic Science, School of Health and Social Work<br>Siriwardena, Aloysius; University of Lincoln, School of Health and Social Care<br>Bath, Philip; University of Nottingham Faculty of Medicine and Health Sciences, Division of Mental Health and Clinical Neuroscience<br>RIGHT-2 Investigators, ,; University of Nottingham Faculty of Medicine and Health Sciences, Division of Mental Health and Clinical Neuroscience

Keywords:	Stroke < NEUROLOGY, ACCIDENT & EMERGENCY MEDICINE, Stroke medicine < INTERNAL MEDICINE

SCHOLARONE™ Manuscripts

I, the Submitting Author has the right to grant and does grant on behalf of all authors of the Work (as defined in the below author licence), an exclusive licence and/or a non-exclusive licence for contributions from authors who are: i) UK Crown employees; ii) where BMJ has agreed a CC-BY licence shall apply, and/or iii) in accordance with the terms applicable for US Federal Government officers or employees acting as part of their official duties; on a worldwide, perpetual, irrevocable, royalty-free basis to BMJ Publishing Group Ltd ("BMJ") its licensees and where the relevant Journal is co-owned by BMJ to the co-owners of the Journal, to publish the Work in this journal and any other BMJ products and to exploit all rights, as set out in our licence.

The Submitting Author accepts and understands that any supply made under these terms is made by BMJ to the Submitting Author unless you are acting as an employee on behalf of your employer or a postgraduate student of an affiliated institution which is paying any applicable article publishing charge ("APC") for Open Access articles. Where the Submitting Author wishes to make the Work available on an Open Access basis (and intends to pay the relevant APC), the terms of reuse of such Open Access shall be governed by a Creative Commons licence – details of these licences and which Creative Commons licence will apply to this Work are set out in our licence referred to above.

Other than as permitted in any relevant BMJ Author's Self Archiving Policies, I confirm this Work has not been accepted for publication elsewhere, is not being considered for publication elsewhere and does not duplicate material already published. I confirm all authors consent to publication of this Work and authorise the granting of this licence.

For peer review only

Time intervals and distances travelled for pre-hospital ambulance stroke care: data from the randomised-controlled ambulance-based Rapid Intervention with Glyceryl trinitrate in Hypertensive stroke Trial-2 (RIGHT-2)

Mark Dixon, paramedic divisional senior clinical leader;[1,2] Jason P Appleton, consultant neurologist;[1,3] Polly Scutt, medical statistician;[1] Lisa J Woodhouse, medical statistician;[1] Lee J Haywood, database programmer;[1] Diane Havard, senior trial manager;[1] Julia Williams, Professor of paramedic science;[4] A Niroshan Siriwardena, Professor of primary and prehospital care;[5] Philip M Bath, Professor of stroke medicine;[1] on behalf of the *RIGHT-2 Investigators.*

[1]Stroke Trials Unit, Division of Mental Health and Clinical Neuroscience, University of Nottingham, Nottingham, UK.

[2]East Midlands Ambulance Service NHS Trust, Nottingham, UK.

[3]Stroke, University Hospitals Birmingham NHS Foundation Trust, Birmingham, UK.

[4]Division of Paramedic Science, School of Health and Social Work, University of Hertfordshire, Hatfield, UK.

[5]School of Health and Social Care, University of Lincoln, Lincoln, UK.

Corresponding author

Philip M Bath, Stroke Trials Unit, Division of Mental Health and Clinical Neuroscience, University of Nottingham, Queen's Medical Centre campus, Nottingham, NG7 2UH UK.

Email: philip.bath@nottingham.ac.uk

Tel: 0115 823 1765

Fax: 0115 823 1767

Word Count

2,859 (Excluding title page, abstract, references, figures and tables, authorship and contributorship statements)

References

36

For peer review only - http://bmjopen.bmj.com/site/about/guidelines.xhtml

ABSTRACT

Objectives

Ambulances offer the first opportunity to evaluate hyperacute stroke treatments. We investigated the conduct of a hyperacute stroke study in the ambulance-based setting with a particular focus on timings and logistics of trial delivery.

Design

Multicentre prospective, single-blind, parallel group randomised controlled trial in eight NHS ambulance services in England and Wales; 54 acute stroke centres. Paramedics enrolled patients with likely stroke into the Rapid Intervention with Glyceryl trinitrate in Hypertensive stroke Trial-2 (RIGHT-2) and administered active transdermal glyceryl trinitrate or sham. Response time intervals, distances travelled and baseline characteristics of patients are described and compared between ambulance services.

Results

Paramedics enrolled 1,149 patients between September 2015 and May 2018. Final diagnosis: intracerebral haemorrhage 13%, ischaemic stroke 52%, TIA 9%, mimic 26%. Timings (minutes) were (median [25, 75 centile]): onset to 999-call 19 [5, 64]; onset to randomisation 71 [45, 116]; total time at scene 33 [26, 46]; depart scene to hospital 15 [10, 23]; randomisation to hospital 24 [16, 34] and onset to hospital 97 [71, 141]. Ambulances travelled (km) 10 [4, 19] from scene to hospital. Timings and distances differed between ambulance service, e.g. onset to randomisation (fastest 53, slowest 77 minutes; $p<0.001$), distance from scene to hospital (least 4, most 20 km; $p<0.001$).

Conclusion

We completed a large pre-hospital stroke trial involving a simple-to-administer intervention across multiple ambulance services. The time from onset to randomisation and modest distances travelled support the applicability of future large-scale paramedic-delivered ambulance-based stroke trials in urban and rural locations.

For peer review only - http://bmjopen.bmj.com/site/about/guidelines.xhtml

Registration

ISRCTN26986053

Funding

British Heart Foundation

Abstract word count

248

Keywords

Ambulance, paramedic, stroke, glyceryl trinitrate, logistics

For peer review only

For peer review only - http://bmjopen.bmj.com/site/about/guidelines.xhtml

STRENGTHS AND LIMITATIONS

- Effective treatment for acute stroke is time dependent and emergency medical services play a vital role in the early recognition, assessment and transportation of stroke patients.
- The first multicentre paramedic-delivered ambulance-based randomised-controlled trial in stroke in the United Kingdom.
- This study provides the first evidence that ambulance-based pre-hospital stroke-research may be performed across multiple NHS organisations.
- Ambulance response time intervals and distances are reported for 1,149 patients.
- Timing and logistic data may not be fully representative of all urban and rural locations due to non-participation of some hospitals within ambulance service regions.

For peer review only

For peer review only - http://bmjopen.bmj.com/site/about/guidelines.xhtml

INTRODUCTION

Routine pre-hospital management of suspected acute stroke involves rapid identification of suspected stroke using a validated stroke screening tool, prompt transport, pre-arrival notification and primary stabilisation to the nearest appropriate receiving stroke centre.[1] The mainstays for hyperacute management of stroke in hospital include urgent neuroimaging, stroke unit care, reperfusion therapy for ischaemic stroke, and blood pressure (BP) lowering for intracerebral haemorrhage.[2] For reperfusion therapies, shortening the time from symptom onset to treatment improves functional outcome and this has become the aim of pre-hospital and in-hospital acute stroke services.[3-6] Thus, ambulance services play a crucial role in assessing, identifying and conveying patients with suspected stroke to primary and comprehensive stroke centres, which may include bypassing local emergency departments.

Timely pre-hospital care for stroke is dependent upon several factors that include rapid recognition of potential stroke and calling for help,[7] ambulance response times encompassing symptom onset to arrival at hospital,[8] distance from scene to hospital,[9] and the accuracy of identifying patients with true stroke or transient ischaemic attack (TIA) from those with a stroke mimic.[10] There are a small, but growing number of studies that explore randomised paramedic-initiated interventions commencing in the ambulance for acute stroke. However, few studies have systematically analysed these parameters and the factors that influence them in acute pre-hospital stroke practice.[4 11-13]

Here, we report the logistics underlying patient recruitment to the Rapid Intervention with Glyceryl trinitrate (GTN) in Hypertensive stroke Trial-2 (RIGHT-2), a large ambulance-based stroke trial in the UK that investigated the efficacy of transdermal glyceryl trinitrate as a paramedic-delivered intervention in suspected acute stroke. Specifically, ambulance response times and distance travelled in this setting are assessed.

For peer review only - http://bmjopen.bmj.com/site/about/guidelines.xhtml

METHODS

RIGHT-2 trial

RIGHT-2 (registration ISRCTN26986053) commenced recruitment in September 2015 with the first participant recruited on 22 October 2015.

RIGHT-2 was a multicentre prospective, single-blind, parallel group randomised controlled trial; the protocol, statistical analysis plan, baseline data, main results and subgroup results in participants with a final diagnosis of intracerebral haemorrhage (ICH) are published.[14-18] Briefly, adult patients with suspected stroke presenting to the emergency service via a 999-call were recruited if they: were FAST-positive (facial weakness, arm weakness, speech abnormality; with test score 2 or 3), had systolic BP of >120mmHg, were within 4 hours of symptom onset, presented to trial-trained paramedics from eight UK ambulance services, and were to be taken to a trial-participating hospital. Patients were randomised to receive transdermal glyceryl trinitrate (GTN) or sham patch in the ambulance and this was continued for three further days during hospital admission.[19] The study was undertaken across eight UK ambulance services (AS): East of England AS (EEAS), East Midlands AS (EMAS), London AS (LAS), South-Central AS (SCAS), South-West AS (SWAS), Welsh AS (WAS), West-Midlands AS (WMAS) and Yorkshire AS (YAS). All participating ambulance services used FAST identification and protocols consistent with national guidelines.

For each eligible patient, the enrolling paramedic assessed capacity and obtained patient or proxy consent (from a relative on the scene, or from the paramedic witnessed by a colleague), completed a written case report form to capture in-ambulance baseline and on-treatment data, and applied the transdermal patch of GTN or sham dressing.[14] Ambulance-related data not recorded at source were confirmed by research paramedics from participating ambulance services after review of control room timing logs or patient care records, and then entered into the trial database.

Timings and distances

Timings were obtained from each ambulance service (time of emergency 999-call, resource dispatch, scene arrival and departure, hospital arrival) and from paramedic records (consent for trial enrolment, randomisation, application of study treatment). Paramedic-documented history provided the time of symptom onset or, where

For peer review only - http://bmjopen.bmj.com/site/about/guidelines.xhtml

unclear, the last known well time.

Distance measurements were calculated from the address or postcode of the emergency location, where available, to the expected stopping point for the ambulance at the destination hospital (accident and emergency or stroke unit entrance) to the nearest 10 metres using Google Maps; one ambulance service was unable to provide postcode information due to time constraints. One ambulance service was able to provide the linear distance from the location of the ambulance at the point of dispatch to the scene of the emergency.

A comparison of urban versus rural ambulance services arbitrarily divided ASs by <25% rural versus >25% rural (as defined in Table 1; Supplemental Table I).

For peer review only

For peer review only - http://bmjopen.bmj.com/site/about/guidelines.xhtml

Table 1. Characteristics of participating ambulance service as of 31st May 2018. Data are numbers (%)

	E&W	EEAS	EMAS	LAS	SCAS	SWAS	WAS	WMAS	YAS
Time in trial (months)	32	27	32	14	4	27	22	14	29
Patients	1149	178	218	202	7	265	89	37	153
Participating hospitals	54	5	10	3	1	13	4	5	13
Area (km^2)	122,065	19,424	16,710	1,605	9,204	25,899	20,735	12,949	15,539
Population									
Overall (x1000)	53,000	5,800	4,800	8,600	7,000	5,300	2,900	5,600	5,338
Living in rural areas† (%)	17.6	28.9	26.7	0.2	20.4	31.6	32.8	15.1	17.5
Strokes (/yr) ††	90,781	9,145	9,246	13,118	7,763	10,442	7,400	8,701	7,931
Adjusted ratio /1000	1.71	1.58	1.92	1.52	1.11	1.97	2.55	1.55	1.49
Call volume (/day)	24,661	2,800	2,500	5,193	1,479	3,077	1,331	3,000	2,336
Participating Ambulance Stations	270	24	50	23	3	73	34	17	63
Paramedics employed	22,000	2,000	1,111	2,864	1,780	1,788	1,310	1,300	1,592
Trained in RIGHT-2	1,492	145	193	325	63	313	165	124	142
Paramedics who recruited	516	58	75	120	6	112	47	23	75
Patients/paramedic	2.22	3.06	2.90	1.68	1.16	2.37	1.89	1.60	2.04

† 2011 Census[20] †† Number of suspected stroke patients assessed face-to-face 2015/6
E&W: England & Wales; EEAS – East of England Ambulance Service NHS Trust; EMAS – East Midlands Ambulance Service NHS Trust; LAS – London Ambulance Service; SCAS – South Central Ambulance Service NHS Foundation Trust; SWAS – South Western Ambulance Service NHS Foundation Trust; WAS – Welsh Ambulance Service NHS Trust; YAS – Yorkshire Ambulance Service NHS Trust.

For peer review only

For peer review only - http://bmjopen.bmj.com/site/about/guidelines.xhtml

Comparison of trial and non-trial patients

One ambulance service provided response time interval and distance data for a cohort (n=49) of confirmed stroke patients who were not enrolled into RIGHT-2 (attended by non-trial trained paramedics) but were transported to the same specialist stroke centres participating in the trial.

Statistical Analysis

Time intervals (in minutes), distances (kilometres, km) and baseline characteristics were compared between ambulance services using chi-square and Kruskal-Wallis (one-way ANOVA on ranks) tests. Multiple comparison procedures (Dunn's with Bonferroni correction) were used to assess which ambulance service differed from the others. Spearman and point-biserial correlations were performed to identify the relationship between baseline variables, times and distances. Data are number (%), median [interquartile range] or mean (standard deviation). Statistical significance was defined overall at $p<0.05$, and at $p<0.001$ for correlation matrices and multiple comparisons. Statistical analyses were conducted with SPSS version 24 (IBM, New York).

Patient and Public Involvement

This study was supported by public members of the trial steering committee who were involved throughout, including in trial design, development, conduct, periodic review and dissemination of results.

For peer review only - http://bmjopen.bmj.com/site/about/guidelines.xhtml

RESULTS

RIGHT-2 recruited 1,149 patients between September 2015 and May 2018. Table 1 outlines patient recruitment across the various participating ambulance services, which collectively covered an area of 122,065 km^2 in England and Wales (i.e. 42% of the land area of these countries). Ambulance services varied considerably in size (1,605 km^2 vs 25,899 km^2), population served per service (2.9M vs 8.6M)[20] and annual stroke events (7,400 vs 13,118) (Table 1). Altogether 1,492 paramedics volunteered to be trained in the trial, of whom 516 (36%) recruited at least one patient. Where two or more trial paramedics were present at scene, the paramedic initiating randomisation was credited. On average, 2.2 patients were recruited by each paramedic who enrolled at least one patient although this varied between ambulance services (1.1 vs 3.1).

Patient characteristics

Of the 1,149 patients recruited, average age was 73 (15) years, female 48%, BP 162 (25)/92 (18) mmHg, Glasgow coma scale (GCS) 13.9 (1.7), and FAST score of three 60% (Supplemental Table II). The final diagnosis varied between ambulance services, with the rate of conditions mimicking acute neurovascular disease ranging from 14.3% to 36.1%. Baseline temperature also varied. Otherwise, baseline characteristics did not differ between ambulance service. As age increased, BP and glucose were higher, and heart rate, FAST and GCS lower (Supplemental Table III). 603 (53%) patients gave informed consent, 431 (38%) by relative and 115 (10%) by paramedic witnessed by a colleague on scene.

Time Intervals

The time intervals for various stages in the journey from stroke scene to hospital are shown in Supplemental Table IV. Overall, the median time from symptom onset to emergency 999-call was 19 [interquartile range 5, 64] minutes and this did not differ between ambulance services (Supplemental Tables IV and V). The median time from 999-call to ambulance dispatch was 3 [1, 7] minutes and varied between ambulance service (1 minute vs 5 minutes). An ambulance resource arrived at scene within 8 [5, 13] minutes from being dispatched (and 10 [6, 16] minutes if only including RIGHT-2 trained paramedics) with this varying between ambulance service (8 minutes vs 12 minutes).

For peer review only - http://bmjopen.bmj.com/site/about/guidelines.xhtml

The median time from onset of symptoms to randomisation was 71 [45, 116] minutes (Table 2, Figure 1) and this varied between ambulance service (53 minutes vs 77 minutes). Significantly, randomisation occurred within 30 and 60 minutes of symptom onset in 104 (9.1%) and 491 (42.9%) participants respectively (Table 2). Ambulance resources spent a median of 33 [26, 46] minutes on scene, though this varied between ambulance services (29 minutes vs 43 minutes) (Supplemental Table IV). Importantly, time on scene did not differ significantly when comparing RIGHT-2 patients versus non-RIGHT-2 patients 34 [26, 44] and 32 [23, 41] minutes respectively (Supplemental Table VI). Transfer time from scene to hospital was a median of 15 [10, 23] minutes, but varied between ambulance service (9 minutes vs 24 minutes) (Supplemental Table IV). The overall time from symptom onset to arrival at hospital was 97 [71, 141] minutes and also varied between ambulance services (86 minutes vs 109 minutes) (Supplemental Table VII). Time at scene was strongly positively correlated with time from scene to hospital (Table 3).

For peer review only

For peer review only - http://bmjopen.bmj.com/site/about/guidelines.xhtml

Table 2. Timings: Symptom onset to randomisation (OTR) (minutes). Data are N (%), median [25, 75 centile]; comparison by Kruskal-Wallis test.

Minutes	E&W	EEAS	EMAS	LAS	SCAS	SWAS	WAS	WMAS	YAS	2p
OTR										
N (%)	1149	178 (15.5)	218 (19.0)	202 (17.6)	7 (0.6)	265 (23.1)	89 (7.7)	37 (3.2)	153 (13.3)	
Median [25, 75 centile]	71 [45, 116]	73 [47, 120]	59 [35, 100]	77 [51, 124]	53 [45, 65]	75 [49, 107]	75 [48, 123]	60 [32, 115]	70 [45, 118]	0.001
N (%)										<0.001
≤30	104 (9.1)	15 (8.4)	38 (17.4)	11 (5.4)	1 (14.3)	16 (6.0)	7 (7.9)	6 (16.2)	10 (6.5)	
31-60	387 (33.8)	63 (35.4)	82 (37.6)	61 (30.2)	3 (42.9)	82 (30.9)	28 (31.5)	13 (35.1)	56 (36.6)	
61-120	394 (34.3)	57 (32.0)	53 (24.3)	76 (37.6)	2 (28.6)	113 (42.6)	32 (36.0)	10 (27.0)	51 (33.3)	
121-180	173 (15.1)	30 (16.9)	33 (15.1)	28 (13.9)	0 (0.0)	40 (15.1)	16 (18.0)	6 (16.2)	20 (13.1)	
181-240	76 (6.6)	12 (6.7)	9 (4.1)	20 (9.9)	0 (0.0)	13 (4.9)	5 (5.6)	2 (5.4)	15 (9.8)	
>240	15 (1.2)	1 (0.5)	3 (1.4)	6 (3.0)	1 (14.3)	1 (0.4)	1 (1.1)	0 (0.0)	2 (0.7)	

For peer review only

For peer review only - http://bmjopen.bmj.com/site/about/guidelines.xhtml

Table 3. Univariate Correlation between severity of symptoms, timings and distance from scene to hospital. Data are Spearman's coefficient (p-value).

	OTR	FAST	GCS	Scene	OTH	STH	Km
OTC	0.836 (<0.001)	-0.130 (<0.001)	0.086 (0.003)	0.05 (0.088)	0.802 (<0.001)	0.007 (0.80)	-0.070 (0.033)
OTR		-0.135 (<0.001)	0.102 (0.001)	0.263 (<0.001)	0.941 (<0.001)	0.244 (<0.001)	-0.61 (0.61)
FAST			-0.157 (<0.001)	-0.066 (0.026)	-0.133 (<0.001)	-0.046 (0.12)	0.803 (0.93)
GCS				-0.008 (0.80)	0.115 (<0.001)	0.084 (0.004)	0.076 (0.017)
Scene					0.326 (<0.001)	0.791 (<0.001)	0.104 (0.002)
OTH						0.403 (<0.001)	0.216 (<0.001)
STH							0.554 (<0.001)

OTC: onset to 999-call; OTR: onset to randomisation; FAST: face, arm, speech, time score. Scene: Total time spent at scene; STH: Time from scene to reach hospital; Km: distance (kilometres) from scene to hospital.

For peer review only

For peer review only - http://bmjopen.bmj.com/site/about/guidelines.xhtml

Distances

The median distance travelled from the postcode of the suspected stroke scene to the receiving hospital was 10.0 [4.4, 18.4] km, with considerable variation between ambulance services (4.1 km vs 19.9 km) (Supplemental Table VIII). Time from scene to hospital was moderately positively correlated with distance from scene to hospital. (Supplemental Figures I:A-G present geographical distribution of randomisation by ambulance service)

Urban versus rural services

When comparing urban and rural ambulance services (Supplemental Table I), there was no difference in receipt of the 999-call to dispatching a resource to scene, nor a difference in onset of symptoms to randomisation. The time spent at scene was marginally longer in rural locations and, as anticipated, both conveyance time and distance to the stroke centre was statistically different.

Comparison of trial and non-trial patients

In the ambulance service with times available for patients not enrolled in the trial, on scene to hospital arrival differed among patients enrolled and not enrolled in RIGHT-2, 10 [0.4, 64.7] versus 16 [7.6, 24.0] minutes (Supplemental Table VIII). The median distance from dispatch location to scene in the ambulance service with this available (EMAS) was 7.3 km [3.5, 12.0].

For peer review only

For peer review only - http://bmjopen.bmj.com/site/about/guidelines.xhtml

DISCUSSION

In this large national prehospital trial, 516 paramedics from eight ambulance services across England and Wales successfully recruited 1,149 participants and transported them to 54 hospitals. Paramedics assessed and diagnosed suspected stroke, consented patients and initiated randomised treatment. Key timings were: onset to 999-call 19 minutes, onset to scene 40 minutes, onset to randomisation 71 minutes, time at scene 33 minutes, randomisation to hospital 24 minutes, and depart scene to hospital arrival 15 minutes; all but the first two differed between ambulance services. The average distance travelled by one ambulance service from dispatch location to scene was 7.3 km and 10.0 km from scene to hospital for all participating ambulance services.

Pre-hospital time intervals in acute stroke have been described previously,[12 21-25] but rarely in randomised trials.[4 11 13] The symptom onset to randomisation time of 71 minutes in RIGHT-2 is consistent with two previous UK ambulance-based stroke trials (RIGHT 55 minutes; Paramedic Initiated Lisinopril For Acute Stroke Treatment (PIL-FAST) 70 minutes)[26 27] although these were small single centre pilot studies undertaken largely in urban settings. The large US Field Administration of Stroke Therapy - Magnesium trial[28] (FAST-MAG) reported a median of 45 minutes from symptom onset to receipt of study drug. Nevertheless, these times are all longer than UK multi-centre ambulance-based trials outside of stroke, notably the AIRWAYS-2 and PARAMEDIC-2 trials in cardiac arrest.[29 30] In PARAMEDIC-2, the onset of symptoms to initiation of treatment in the intervention group was just 21.5 minutes. The most important driver of this difference is most likely shorter onset to call times for cardiac arrest patients than for stroke, and suspected stroke may require more complex assessment both by call handlers and by paramedics on scene. Additional contributors are that cardiac arrest is allocated the highest dispatch priority, an immediate response and patients receive immediate trial treatment with emergency waiver of consent.

The explanation for differences in timings is probably multifactorial but the degree of urban versus rural population is one likely explanation. This was apparent for time spent at scene and both time and distance to hospital. As expected, there were no differences for receipt of call to dispatch, arrival of RIGHT-2 trained paramedic at scene nor onset of symptoms to randomisation.

For peer review only - http://bmjopen.bmj.com/site/about/guidelines.xhtml

There are several strengths of this study. First, RIGHT-2 involved eight of 11 ambulance services in England and Wales. Of those not participating, two were unable to join because they were involved in another ambulance-based stroke trial[31] and the other involved hospitals that were concerned about adversely impacting on recruitment to commercial trials. 516 paramedics (of 1,492 trained in RIGHT-2 procedures) consented and randomised a large number of participants, adhered to the protocol and completed specific data recording.

The consent model applied in RIGHT-2 is unlike any other ambulance-based studies to date as many have relied upon models of waiver of consent or deferred consent due to complexities of emergency presentations that could impact upon decision making and mental capacity or short intervention windows. RIGHT-2 sought to test a novel consent approach due to the varying severity of stroke where stroke patients' ability to consent in an informed manner to participate in a clinical study should not be overlooked but where the condition was of a severity rendering the patient unable to consent also provide an opportunity to participate by legal representative assent (relative, friend, carer or paramedic following capacity assessment) minimising selection bias. Second, the protocol required flexibility and adaptation to align with individual operational processes specific to each ambulance service to ensure successful delivery of the trial. Third, detailed logistic information on timing and distances travelled were collected. Last, the results highlight the successful delivery of a simple, ambulance-based intervention with 43% of patients receiving the intervention within two-hours of symptom onset without compromising time on scene required to complete additional research activity.

There are also several study limitations. Firstly, it is recognised that not every receiving stroke unit within each ambulance service could participate in RIGHT-2 due to capacity and competing research,[31 32] (this included concurrent commercial and post-arrival trials). Therefore, it must be considered that the timing and logistic data of participating hospitals may not be fully representative of all urban and rural locations. However, the intention was not to assess the differences between urban and rural settings, but to shed light on the conduct and deliverability of a pre-hospital intervention in stroke where time and distance may impede access to specialist stroke services. Furthermore, stroke unit hours of operation varied across the 54 centres with a small number of sites not accepting patients outside working hours which

For peer review only - http://bmjopen.bmj.com/site/about/guidelines.xhtml

impacted paramedics' decisions to randomise. This reduces the reflection of real-world emergency stroke care. Additionally, it is acknowledged that the recruitment criteria were broad which resulted in a higher than anticipated proportion of stroke mimics. To mitigate this, mobile stroke unit care is an emerging field where imaging and definitive care delivery at the scene reduces time delays in stroke[33] and could offer improved confidence and precision of diagnosis for prehospital trial enrolment.

Recognising that 516 of 1,492 RIGHT-2 trained paramedics (36%) identified and randomised eligible patients, this is consistent with other trials in pre-hospital stroke.[26] This, in part, is due to the voluntary participation of paramedics in research where records suggest that only one third of the paramedic workforce participate.[34] Further, in a UK system where response time is one benchmark of the quality of ambulance service provision, ambulance dispatchers are not able to assign specific research-trained personnel to specific emergency calls, instead allocating the nearest available resource to attend. Low recruitment must be considered during the development of ambulance-based trials and this factor alone has previously resulted in extended recruitment phases, retraining of researchers and extensive study drug availability to achieve pre-planned sample sizes.[28 35 36]

Finally, this paper does not capture the time-interval between arrival at hospital and the ambulance becoming available for the next emergency call (hospital turnaround). During this period, ambulance staff hand-over the patient to hospital staff, complete relevant documentation and prepare the vehicle for the next assignment. A rapid hospital turnaround is important for making the vehicle available for waiting emergency calls. Whilst the addition of research activity at scene may not delay enrolled patient treatment, it is possible that delay required to complete additional research activity steps after patient handover may prolong the turnaround phase.

In summary, we completed a large pre-hospital stroke trial involving a simple-to-administer intervention across multiple ambulance services. The time from onset to randomisation and modest distances travelled support the applicability of future large-scale paramedic-delivered ambulance-based stroke trials in urban and rural locations. Nevertheless, pre-hospital time intervals and distances from scene-to-hospital varied by ambulance service and this was, at least in part, explained by the type of urban versus rural population. Although our results may not be generalisable to all ambulance service settings, they do inform future developments in ambulance-based

For peer review only - http://bmjopen.bmj.com/site/about/guidelines.xhtml

stroke care and provide support to the deliverability of future large-scale multicentre pre-hospital paramedic-delivered ambulance-based acute stroke trials.

For peer review only

For peer review only - http://bmjopen.bmj.com/site/about/guidelines.xhtml

ACKNOWLEDGEMENTS

We thank the patients who participated in this trial and their relatives, the clinical and research teams of the various ambulance services and hospitals, and the paramedics who participated, recruited and treated the patients. We acknowledge support of the English National Institute for Health Research (NIHR) Clinical Research Network, Health and Care Research Wales, and that the coordination between multiple ambulance services and hospitals, and large recruitment would not have been possible without NIHR network support. A complete list of the Rapid Intervention with Glyceryl trinitrate in Hypertensive stroke Trial-2 (RIGHT-2) Investigators is provided in the primary publication.[17] We acknowledge Eivind Berge who contributed to this paper but sadly passed away prior to publication.

SOURCES OF FUNDING

This work was supported by the British Heart Foundation [grant number CS/14/4/30972] and sponsored by the University of Nottingham.

ETHICAL APPROVAL

RIGHT-2 was approved by the UK regulator (Medicines and Healthcare products Regulatory Agency, reference: 03057/0064/001–0001; Eudract 2015–000115–40) and national research ethics committee (IRAS: 167115) and was adopted by the National Institute for Health Research Clinical Research Network.

TRANSPARENCY

The lead and senior authors affirms that this manuscript is an honest, accurate, and transparent account of the study being reported; that no important aspects of the study have been omitted; and that any discrepancies from the study as planned have been explained.

DATA AVAILABILITY STATEMENT

Individual participant data are shared with the Blood pressure in Acute Stroke Collaboration (BASC) and Virtual International Stroke Trials Archive (VISTA). No further data available.

For peer review only - http://bmjopen.bmj.com/site/about/guidelines.xhtml

AUTHORSHIP STATEMENTS

The following are members of RIGHT-2 (Rapid Intervention with Glyceryl trinitrate in Hypertensive stroke Trial-2) trial steering committee and critically revised the manuscript for important intellectual content:

Mark Dixon, paramedic divisional senior clinical leader[1,2], Jason P Appleton, consultant neurologist[1,22], Polly Scutt, medical statistician[1], Lisa J Woodhouse, medical statistician[1], Lee J Haywood, database programmer[1], Harriet Howard, senior trial coordinator[1], Diane Havard, senior trial manager[1], Nikola Sprigg, consultant stroke physician[1,3], Tom Robinson, Professor of stroke medicine[14], Christopher Price, clinical reader in stroke medicine[15], Craig Anderson, Professor of neurology and epidemiology[16], Grant Mair, senior clinical lecturer in neuroradiology[17], Else C Sandset, consultant neurologist[18], Jeffrey Saver, Professor of clinical neurology[19], Christine Roffe, stroke physician[20], Keith Muir, consultant neurologist[21], Kailash Krishnan, consultant neurologist[1,3], Joanna M Wardlaw, Professor of applied neuroimaging[17], Julia Williams, Professor of paramedic science[4], A Niroshan Sirawardena, Professor of primary and prehospital care[2, 5], Philip M Bath, Professor of stroke medicine[1,3]

CONTRIBUTORSHIP STATEMENTS

The following colleagues contributed to trial implementation, data collection and revision of the draft paper: Roderick Johnson, paramedic clinical operations manager[2], Debbie Shaw, clinical audit and research manager[2], Robert Spaight, head of research and audit[2], Larissa Prothero, research paramedic[6], Theresa Foster, research manager[6], Rachael T Fothergill, Research Manager[7], Heather Cole, Research paramedic[7], Joanna Lazarus, Research paramedic[7], Helen Werts, research paramedic[7], Neil Thomson, Medical Director[7,] Helen Pocock, senior research paramedic[8], Kurtis Poole, research paramedic[8], Ed England, research manager[8], Maria Robinson, research manager[9], Katherine Zorab, Quality Improvement Paramedic[9], Adrian South, Clinical Director[9], Catrin Convery, research paramedic[10], Tim Pearce, Paramedic Team Leader[10], Nigel Rees, Head of Research and Development[10], Joshua Miller, research paramedic[11], Imogen Gunson, research paramedic[11], Andrew Rosser, head of research[11], Matthew Ward, consultant paramedic[11], Kelly Hird, research paramedic[12], Jacqui Crossley, assistant clinical director[12].

For peer review only - http://bmjopen.bmj.com/site/about/guidelines.xhtml

For peer review only

Author Affiliations

[1]Stroke Trials Unit, Division of Mental Health and Clinical Neuroscience, University of Nottingham, Queens Medical Centre Campus, Nottingham UK.
[2]East Midlands Ambulance Service NHS Trust, Mellors Way, Nottingham, UK.
[3]Stroke, Nottingham University Hospitals NHS Trust, Nottingham, UK.
[4]Division of Paramedic Science, School of Health and Social Work, University of Hertfordshire, Hatfield, UK.
[5]School of Health and Social Care, University of Lincoln, Lincoln, UK.
[6]East of England Ambulance Service NHS Trust, Whiting Way, Melbourn, UK.
[7]London Ambulance Service NHS Trust, Waterloo Road, London, UK.
[8]South Central Ambulance Service NHS Foundation Trust, Talisman Road, Bicester, UK.
[9]South Western Ambulance Service NHS Foundation Trust, Eagle Way, Exeter, UK.
[10]Welsh Ambulance Service NHS Trust, St Asaph, Wales, UK.
[11]West Midlands Ambulance Service University NHS Foundation Trust, Waterfront Way, West Midlands, UK.
[12]Yorkshire Ambulance Service NHS Trust, Brindley Way, Wakefield, UK.
[14]Department of Cardiovascular Sciences, University of Leicester, Leicester, UK.
[15]Institute of Neuroscience, Newcastle University, Newcastle Upon Tyne, UK.
[16]The George Institute for Global Health, Peking University Health Science Centre, Beijing, China.
[17]Centre for Clinical Brain Sciences, The University of Edinburgh, UK.
[18]Department of Neurology, Oslo University Hospital, Norway.
[19]Neurological Services, UCLA Medical Center, Santa Monica, United States of America
[20]University Hospital of North Midlands NHS Trust, Stoke-on-Trent, UK.
[21]Institute of Neuroscience and Psychology, University of Glasgow, UK.
[22] Stroke, University Hospitals Birmingham NHS Foundation Trust, Birmingham, UK.

The corresponding author attests that all listed authors meet authorship criteria and that no others meeting the criteria have been omitted.

COMPETING INTERESTS

JPA was funded in part by the BHF during the conduct of the study and is supported by the National Institute for Health Research (NIHR) West Midlands Health and Care Research Scholars Programme. CSA reports grants from National Health and Medical Research Council (NHMRC) of Australia, grants from Takeda, and personal fees from Takeda, Amgen, and Boehringer Ingelheim outside of the submitted work. MD was funded by the BHF during the conduct of the study. TJE, and AAM report grants from BHF during the conduct of the study. GM is supported by NHS Lothian Research and Development Office and reports grants from The Stroke Association (TSA), The Royal College of Radiologists. CIP reports grants from Nottingham University and BHF during

For peer review only - http://bmjopen.bmj.com/site/about/guidelines.xhtml

the conduct of the study. TGR is an NIHR Senior Investigator, and reports grants from BHF during the conduct of the study. CR reports grants from NIHR Health Technology Assessment during the conduct of the study; personal fees from Allergan, Air Liquide, Merz, Boehringer, Bayer, Johnson & Johnson, Sanofi and Emtensor; non-financial support from European Stroke Conference and Trident; and other support from Firstkind Medical, Medtronic and Brainomix outside the submitted work. PMR reports grants from Wellcome Trust during the conduct of the study. ECS reports personal fees from Novartis and Bayer outside of the submitted work. JMW reports grants from the BHF during the conduct of the study; and grants from Medical Research Council, Chief Scientist Office, Leducq, EU H2020, TSA, BHF and Alzheimer's Society outside the submitted work. NSp reports grants from BHF and RCUK, during the conduct of the study. PMB is Stroke Association Professor of Stroke Medicine and is a National Institute Health Research (NIHR) Senior Investigator. He reports grants from British Heart Foundation (BHF) during the conduct of the study; personal fees and other fees from Sanofi, Nestlé, DiaMedica, Moleac, Platelet Solutions, Phagenesis, and ReNeuron, outside the submitted work. All other authors declare no competing interests.

COPYRIGHT

The Corresponding Author has the right to grant on behalf of all authors and does grant on behalf of all authors, an exclusive licence (or nonexclusive for government employees) on a worldwide basis to the BMJ Publishing Group Ltd to permit this article (if accepted) to be published in BMJ editions and any other BMJPGL products and sublicenses such use and exploit all subsidiary rights, as set out in our licence.

For peer review only

For peer review only - http://bmjopen.bmj.com/site/about/guidelines.xhtml

References

1. Joint Royal Colleges Ambulance Liaison Committee, Association of Ambulance Chief Executives. *JRCALC Clinical Guidelines*. Bridgwater: Class Professional Publishing, 2019.
2. Royal College of Physicians. Intercollegiate Stroke Working Party. National clinical guidelines for stroke.**5th Edition 2016**.
3. Meretoja A, Strbian D, Mustanoja S, et al. Reducing in-hospital delay to 20 minutes in stroke thrombolysis. *Neurology* 2012;**79**(4):306-13.
4. Koch P, Kunz A, Ebinger M, et al. Influence of Distance to Scene on Time to Thrombolysis in a Specialized Stroke Ambulance. *Stroke* 2016;**47**(8):2136-40.
5. Emberson J, Lees K, Lyden P, et al. Effect of treatment delay, age and stroke severity on the effects of intravenous thrombolysis with alteplase for acute ischaemic stroke: a meta-analysis of individual patient data from randomised trials. *Lancet* 2014;**384**(9958):1929-35.
6. Bourcier R, Goyal M, Liebeskind DS, et al. Association of Time From Stroke Onset to Groin Puncture With Quality of Reperfusion After Mechanical Thrombectomy: A Meta-analysis of Individual Patient Data From 7 Randomized Clinical Trials. *JAMA Neurol* 2019;**76**(4):405-11.
7. Wolters FJ, Paul NL, Li L, et al. Sustained impact of UK FAST-test public education on response to stroke: a population-based time-series study. *Int J Stroke* 2015;**10**(7):1108-14.
8. Ong ME, Cho J, Ma MH, et al. Comparison of emergency medical services systems in the pan-Asian resuscitation outcomes study countries: Report from a literature review and survey. *Emerg Med Australas* 2013;**25**(1):55-63.
9. Simonsen SA, Andresen M, Michelsen L, et al. Evaluation of pre-hospital transport time of stroke patients to thrombolytic treatment. *Scandinavian Journal of Trauma, Resuscitation & Emergency Medicine* 2014;**22**:65.
10. Oostema JA, Konen J, Chassee T, et al. Clinical predictors of accurate prehospital stroke recognition. *Stroke* 2015;**46**(6):1513-7.
11. Ramanujam P, Castillo E, Patel E, et al. Prehospital transport time intervals for acute stroke patients. *J Emerg Med* 2009;**37**(1):40-5.
12. Simonsen SA, Andresen M, Michelsen L, et al. Evaluation of pre-hospital transport time of stroke patients to thrombolytic treatment. *Scand J Trauma Resusc Emerg Med* 2014;**22**:65.
13. Price CI, Shaw L, Islam S, et al. Effect of an Enhanced Paramedic Acute Stroke Treatment Assessment on Thrombolysis Delivery During Emergency Stroke Care: A Cluster Randomized Clinical Trial. *JAMA Neurol* 2020;**77**(7):840-48.
14. Appleton JP, Scutt P, Dixon M, et al. Ambulance-delivered transdermal glyceryl trinitrate versus sham for ultra-acute stroke: Rationale, design and protocol for the Rapid Intervention with Glyceryl trinitrate in Hypertensive stroke Trial-2 (RIGHT-2) trial (ISRCTN26986053). *Int J Stroke* 2017;**0**(0):1-16.
15. Scutt P, Appleton JP, Dixon M, et al. Statistical analysis plan for the 'Rapid Intervention with Glyceryl trinitrate in Hypertensive stroke Trial-2 (RIGHT-2)'. *Eur J Stroke* 2018;**3**(2):193-96.
16. Bath PM, Scutt P, Appleton JP, et al. Baseline characteristics of the 1149 patients recruited into the Rapid Intervention with Glyceryl trinitrate in Hypertensive stroke Trial-2 (RIGHT-2) randomized controlled trial. *Int J Stroke* 2019;**14**(3):298-305.
17. RIGHT-2 Investigators. Prehospital transdermal glyceryl trinitrate in patients with ultra-acute presumed stroke (RIGHT-2): an ambulance-based, randomised, sham-controlled, blinded, phase 3 trial. *Lancet* 2019;**393**(10175):1009-20.

For peer review only - http://bmjopen.bmj.com/site/about/guidelines.xhtml

18. Bath PM, Woodhouse LJ, Krishnan K, et al. Pre-hospital transdermal glyceryl trinitrate for ultra-acute intracerebral haemorrhage- data from the RIGHT-2 trial. *Stroke* 2019;**In press**.
19. Appleton JP, Scutt P, Dixon M, et al. Ambulance-delivered transdermal glyceryl trinitrate versus sham for ultra-acute stroke: Rationale, design and protocol for the Rapid Intervention with Glyceryl trinitrate in Hypertensive stroke Trial-2 (RIGHT-2) trial (ISRCTN26986053). *Int J Stroke* 2017:1747493017724627.
20. Office for National Statistics. 2011 Census Analysis - Comparing Rural and Urban Areas of England and Wales. In: Statistics OfN, ed. London: Office for National Statistics, 2013.
21. Citerio G, Galli D, Pesenti A. Early stroke care in Italy - a steep way ahead: an observational study. *Emerg Med J* 2006;**23**:608-11.
22. Puolakka T, Vayrynen T, Happola O, et al. Sequential analysis of pretreatment delays in stroke thrombolysis. *Acad Emerg Med* 2010;**17**(9):965-9.
23. Mosley I, Nicol M, Donnan G, et al. Stroke symptoms and the decision to call for an ambulance. *Stroke* 2007;**38**(2):361-6.
24. Patel MD, Brice JH, Moss C, et al. An evaluation of emergency medical services stroke protocols and scene times. *Prehosp Emerg Care* 2014;**18**(1):15-21.
25. Drenck N, Viereck S, Bækgaard JS, et al. Pre-hospital management of acute stroke patients eligible for thrombolysis - an evaluation of ambulance on-scene time. *Scand J Trauma Resusc Emerg Med* 2019;**27**(1):10.1186/s13049-018-0580-4.
26. Shaw L, Price C, McLure S, et al. Paramedic Initiated Lisinopril For Acute Stroke Treatment (PIL-FAST): results from the pilot randomised controlled trial. *Emerg Med J* 2014;**31**(12):994-9.
27. Ankolekar S, Fuller M, Cross I, et al. Feasibility of an Ambulance-Based Stroke Trial, and Safety of Glyceryl Trinitrate in Ultra-Acute Stroke The Rapid Intervention With Glyceryl Trinitrate in Hypertensive Stroke Trial (RIGHT, ISRCTN66434824). *Stroke* 2013;**44**(11):3120-28.
28. Saver J, Starkman S, Eckstein M, et al. Prehospital use of magnesium sulfate as neuroprotection in acute stroke. *N Engl J Med* 2015;**372**(6):528-36.
29. Perkins GD, Ji C, Deakin CD, et al. A Randomized Trial of Epinephrine in Out-of-Hospital Cardiac Arrest. *N Engl J Med* 2018;**379**(8):711-21.
30. Benger JR, Kirby K, Black S, et al. Effect of a Strategy of a Supraglottic Airway Device vs Tracheal Intubation During Out-of-Hospital Cardiac Arrest on Functional Outcome: The AIRWAYS-2 Randomized Clinical Trial. *JAMA* 2018;**320**(8):779-91.
31. Price CI, Shaw L, Dodd P, et al. Paramedic Acute Stroke Treatment Assessment (PASTA): study protocol for a randomised controlled trial. *Trials* 2019;**20**(1):121.
32. Muñoz-Venturelli P, Arima H, Lavados P, et al. Head Position in Stroke Trial (HeadPoST)--sitting-up vs lying-flat positioning of patients with acute stroke: study protocol for a cluster randomised controlled trial. *Trials* 2015;**16**:256.
33. Fassbender K, Merzou F, Lesmeister M, et al. Impact of mobile stroke units. *J Neurol Neurosurg Psychiatry* 2021.
34. Pocock H, Deakin CD, Quinn T, et al. Human factors in prehospital research: lessons from the PARAMEDIC trial. *Emergency Medicine Journal* 2016;**33**(8):562-8.
35. Hougaard K, Hjort N, Zeidler D, et al. Remote ischemic perconditioning as an adjunct therapy to thrombolysis in patients with acute ischemic stroke: a randomized trial. *Stroke* 2013;**45**:159-67.

For peer review only - http://bmjopen.bmj.com/site/about/guidelines.xhtml

36. De Luca A, Toni D, Lauria L, et al. An emergency clinical pathway for stroke patients - results of a cluster randomised trial (isrctn41456865). *Bmc Health Services Research* 2009;**9**:10.

For peer review only

For peer review only - http://bmjopen.bmj.com/site/about/guidelines.xhtml

Figure 1. Box Plot of Onset to Randomisation

See separate file

For peer review only

For peer review only - http://bmjopen.bmj.com/site/about/guidelines.xhtml

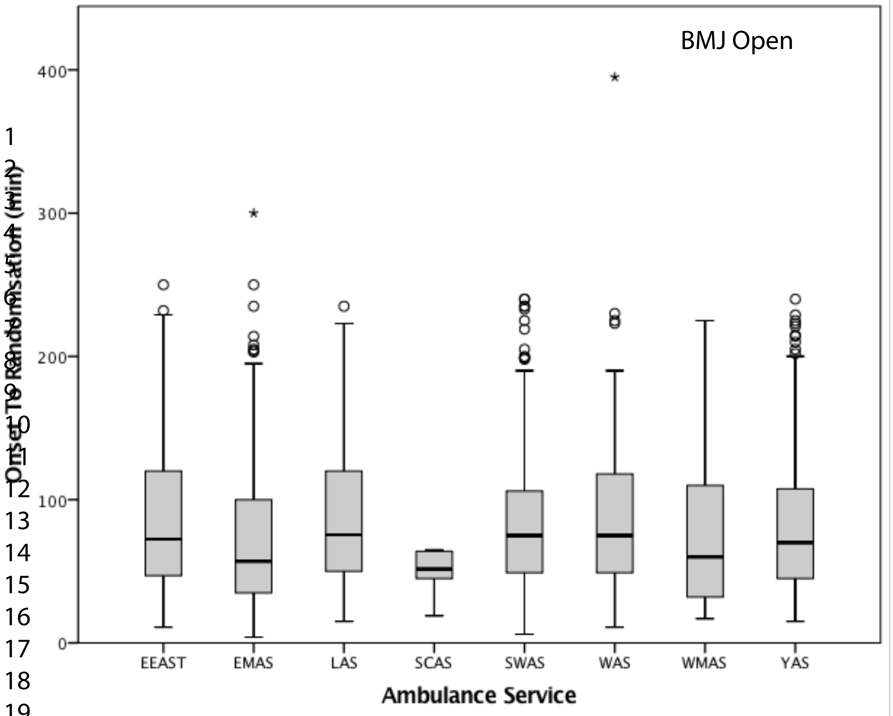
Onset To Randomisation (min)
400
300
200
100
0
EEAST
EMAS
LAS
SCAS
SWAS
WAS
WMAS
YAS
Ambulance Service

SUPPLEMENTAL MATERIAL

Time intervals and distances travelled for pre-hospital ambulance stroke care: data from the Rapid Intervention with Glyceryl trinitrate in Hypertensive stroke Trial-2 (RIGHT-2)

TABLE OF CONTENTS

	Page
Title	1
Supplemental Background	1
Supplemental Methods	1
Supplemental Results	2
List of supplemental Tables and Figures	5
Supplemental Tables (start from)	6
Supplemental Figures (start from)	14
Supplemental References	22

SUPPLEMENTAL BACKGROUND

In the 2011 census of England and Wales, 43.7 million people lived in urban areas (defined as an area with greater than 10,000 inhabitants[1]) with 9.3 million people (17.6%) living in rural areas. Conversely, rural areas cover 85% of the land area of England and Wales. The proportion of urban and rural populations covered varied between ambulance service with rural ranging from 0.2% (LAS) to 32.8% (WAS).

SUPPLEMENTAL METHODS

RIGHT-2 trial

Patients were excluded if they had any of the following: resided in a nursing home, hypoglycaemia (<2.5mmol/l), evidence of seizure at presentation, known to have a terminal illness, taken sildenafil (or equivalent) within 24 hours, or previously been enrolled into RIGHT-2.

For peer review only

Patients were randomised (1:1) to receive either GTN patches or sham patches. A randomisation sequence was generated by the trial programmer at the Nottingham Stroke Trials Unit using random-permuted fixed-size blocks stratified by ambulance station. Identical looking numbered treatment packs were sent in blocks (four treatment packs per block) to each ambulance station. Trial-trained paramedics only carry one treatment pack at any time.

Participants were taken to hospitals with an acute stroke service - these joined the trial depending on capacity and feasibility to receive enrolled patients and deliver the trial protocol.[2] Information on participants was entered into the main trial database by hospital-based research staff, including the data collected in the ambulance (baseline, during treatment) and in hospital (admission, day 2, discharge or death, final diagnosis).

The trial was funded by the British Heart Foundation, sponsored by the University of Nottingham, had Health Research Authority ethics committee approval, and was eligible for National Institute of Health Research Clinical Research Network support.

Data sharing

Individual participant data will be shared with the Blood pressure in Acute Stroke Collaboration (BASC) and Virtual International Stroke Trials Archive (VISTA). From 1 Jan 2022, the Chief Investigator (with approval from the Trial Steering Committee as necessary) will consider other requests to share individual participant data via email at: right-2@nottingham.ac.uk. We will require a protocol detailing hypothesis, aims, analyses, and intended tables and figures. Where possible, we will perform the analyses; alternatively, de-identified data and a data dictionary will be supplied for the necessary variables for remote analysis. Any sharing will be subject to a signed data access agreement. Ultimately, the entire trial dataset will be published.

SUPPLEMENTAL RESULTS

Although all 10 stroke-receiving hospitals participated in the trial in the EMAS area, not all hospitals in the other seven ambulance services took part in the trial (range 1-13; Table 1).

For peer review only - http://bmjopen.bmj.com/site/about/guidelines.xhtml

Time Intervals

The extended time from 999 call to dispatch of 5 minutes for SWAS was due, in part, to a service-wide evaluation of a new ambulance dispatch model (Ambulance Response Programme). The extended time from dispatch to arrival at scene of 12 minutes for WAS is likely to reflect, in part, the high proportion of rural-based patients in Wales and the WAS Clinical Response Model which was introduced during the trial and differs to England.

The time on scene in EMAS was comparable to a cohort of non-RIGHT-2 stroke patients despite the addition of consent, randomisation and treatment activities (34 [26, 44] vs 32 [23, 41] minutes, p=0.12) (Supplemental Table VI).

RIGHT-2 treatment was administered to 910 (80%) patients prior to departing scene and 239 (20%) patients en-route to hospital highlighting the speed and simplicity of the intervention.

Multiple comparison testing for Supplemental Table IV:

Significant differences existed between ambulance service with regards to timings:

- 999 call to dispatch: LAS differs from EEAS p<0.001, EMAS p<0.001, SCAS p=0.011, SWAS p=0.008, WAS p<0.001 WMAS p=0.015 and YAS p=0.002. SWAS differs from EEAS p<0.001, EMAS p<0.001, SCAS p=0.001, WAS p<0.001 and YAS p<0.001
- Dispatch to arrival at scene: LAS differs from EEAS p=0.022, YAS p<0.001, SWAS p<0.001 and WAS p<0.001. EMAS differs from YAS p=0.024, SWAS p<0.001 and WAS p=0.003. EEAS differs from WAS p=0.036
- Dispatch to arrival of RIGHT-2 Paramedic: LAS differs from SWAS p=0.012, EEAS P=0.019, YAS p=0.002 and WAS p=0.003. EMAS differs from YAS p=0.037 and WAS p=0.030
- Time of arrival on scene to randomisation: WMAS differs from EEAS p=0.011, WAS p=0.015 and LAS p=0.001. EMAS differs from SWAS p<0.001, EEAS p<0.001, WAS p<0.001 and LAS p<0.001. YAS differs from EEAS p=0.003, WAS p=0.013 and LAS p<0.001
- Onset to randomisation: EMAS differs from SWAS p=0.021 and LAS p<0.001

For peer review only

- Consent to randomisation: LAS differs from WMAS p=0.030, SWAS p<0.001, EMAS p<0.001, YAS p<0.001, WAS p<0.001 and EEAS p<0.001. SWAS differs from EEAS p=0.003
- Time on scene: EEAS differs from LAS p<0.001, YAS p=0.006, EMAS p=0.046. LAS differs from WAS p=0.001
- Depart scene to arrival at hospital: WMAS differs from EMAS p=0.018, EEAS p<0.001, WAS p<0.001 and SWAS p<0.001. YAS differs from EEAS p=0.003, WAS p=0.002 and SWAS p<0.001. SWAS differs from LAS p<0.001 and EMAS p<0.001
- Onset to hospital: EMAS differs from SWAS p=0.014 and WAS p=0.043. LAS differs from EEAS p<0.001, EMAS p<0.001, SWAS p<0.001, WAS p<0.001, WMAS p<0.001 and YAS p<0.001
- Randomisation to treatment: LAS differs from EEAS p<0.001, EMAS p<0.001, SWAS p<0.001, SCAS p<0.001, WAS p<0.001, WMAS p<0.001 and YAS p<0.001
- Randomisation to hospital: SWAS differs from YAS p<0.001 and WMAS p=0.023

Distances

Although it was not possible to collect information on where the ambulance was when dispatched to the scene for seven ambulance services, this information was available for EMAS; the median linear distance from point of dispatch to the stroke scene was 7.3 [3.5, 12.0] km.

For peer review only

For peer review only - http://bmjopen.bmj.com/site/about/guidelines.xhtml

LIST OF SUPPLEMENTAL TABLES AND FIGURES

Supplemental Tables	
I	Comparison of timings and conveyance distance by urban versus rural geography
II	Demographic and baseline characteristics of patients enrolled into RIGHT-2
III	Univariate correlations between baseline characteristics for age, sex, heart rate, systolic blood pressure and time from onset of symptoms to randomisation
IV	Timings (in minutes) by ambulance service
V	Timings: Onset of symptoms to 999 call
VI	Comparison of EMAS stroke patients in RIGHT-2 versus non-RIGHT-2
VII	Timings: Symptom onset to arrival at hospital
VIII	Conveyance distances

Supplemental Figures	
I	Distribution of Randomisation by Ambulance Service
A	EMAS
B	EEAS
C	SCAS
D	SWAS
E	WAS
F	WMAS
G	YAS

For peer review only

SUPPLEMENTAL TABLE I. Comparison of timings and conveyance distance by urban versus rural geography. Data are median [25, 75 centile]; comparison by Mann-Whitney U test with difference (95% confidence intervals)

	Urban	Rural	Difference (95% CI)	2p
% rural	13.3	30.0	-	-
Timing (minutes)				
Onset to 999 call	23 [8, 70]	17 [5, 58]	19 (17, 23)	0.003
999 call to dispatch	3 [1, 7]	3 [1, 8]	3 (3, 3)	0.17
Onset to R2 scene	47 [24, 95]	43 [22, 84]	45 (42, 48)	0.06
Onset to randomisation	73 [47, 121]	70 [45, 114]	71 (68, 75)	0.10
Arrive to depart scene	29 [22, 35]	32 [24, 41]	31 (30, 31)	<0.001
Depart scene to hospital	12 [9, 18]	17 [10, 25]	15 (14, 16)	<0.001
Distance (km)				
Conveyance	5.7 [3.3, 10.6]	11.7 [5.0, 20.4]	9.5 (8.6, 10.3)	<0.001

Urban: LAS, WMAS, YAS, SCAS; rural: EMAS, EEAS, SWAS, WAS

For peer review only

SUPPLEMENTAL TABLE II. Demographic and baseline characteristics of patients enrolled into RIGHT-2

Data are number (%), median [interquartile range] or mean (standard deviation). Comparisons between ambulance services using Chi-square test, Kruskal-Wallis or one-way ANOVA.

Characteristic	E&W	EEAS	EMAS	LAS	SCAS	SWAS	WAS	WMAS	YAS	2p
Patients (%)	1149	178 (15.5)	218 (19.0)	202 (17.6)	7 (0.6)	265 (23.1)	89 (7.7)	37 (3.2)	153 (13.3)	-
Consent from (%)										0.58
Patient	603 (52.5)	96 (52.2)	111 (50.9)	117 (57.9)	4 (57.1)	131 (49.4)	48 (53.9)	18 (48.6)	81 (52.9)	
Relative/Friend	431 (37.5)	73 (41.0)	90 (41.3)	70 (34.7)	1 (14.3)	102 (38.5)	23 (25.8)	15 (40.5)	57 (37.3)	
Paramedic	115 (10.0)	12 (6.7)	17 (7.8)	15 (7.4)	2 (28.6)	32 (12.1)	18 (20.2)	4 (10.8)	15 (9.8)	
Age (years)	72.5 (14.6)	73.4 (13.4)	71.6 (13.9)	70.4 (16.3)	76.6 (15.4)	74.4 (14.7)	74.7 (14.8)	70.2 (17.2)	71.1 (13.7)	0.34
<80 (%)	714 (62.1)	114 (64.0)	144 (66.1)	131 (64.9)	3 (42.9)	150 (56.6)	48 (53.9)	22 (59.5)	102 (66.7)	
>=80 (%)	435 (37.9)	64 (36.0)	74 (33.9)	71 (35.1)	4 (57.1)	115 (43.4)	41 (46.1)	15 (40.2)	51 (33.3)	
Female (%)	555 (48.3)	83 (46.6)	98 (45.0)	106 (52.5)	1 (14.3)	134 (50.6)	44 (49.4)	16 (43.2)	73 (47.7)	
FAST (/3)	2.6 (0.51)	2.5 (0.53)	2.6 (0.50)	2.5 (0.50)	2.1 (0.38)	2.6 (0.51)	2.6 (0.21)	2.6 (0.55)	2.7 (0.50)	0.31
3 N(%)	692 (60.1)	98 (55.1)	137 (62.8)	107 (53.0)	1 (14.3)	166 (62.9)	56 (63.6)	22 (59.5)	103 (67.3)	
2 N(%)	446 (8.8)	77 (43.3)	80 (36.7)	95 (47.0)	9 (85.7)	95 (36.0)	31 (35.2)	14 (37.8)	48 (31.14)	
1 N(%) †	11 (1.0)	3 (1.7)	1 (0.5)	0 (0.0)	0 (0.0)	3 (1.1)	1 (1.1)	1 (2.7)	2 (1.3)	
GCS (/15)	13.9 (1.7)	13.6 (1.8)	14.0 (1.7)	13.9 (1.7)	13.7 (1.8)	14.0 (1.6)	13.9 (1.7)	13.9 (1.7)	13.9 (1.7)	0.50
<14	302 (26)	57 (32.0)	52 (23.9)	49 (24.3)	2 (28.6)	66 (24.9)	26 (29.2)	11 (29.7)	39 (25.5)	0.65
Haemodynamics										
SBP (mmHg)	162.1 (25.1)	159.38 (25.4)	164.4 (27.4)	163.9 (26.2)	166.4 (27.7)	161.3 (25.0)	162.8 (26.3)	167.7 (28.4)	162.2 (26.8)	0.49
DBP (mmHg)	91.6 (17.9)	90. (21.1)	93.7 (19.4)	92.1 (20.4)	93.0 (12.4)	91.2 (17.9)	92.5 (17.7)	90.5 (24.4)	91.6 (18.6)	0.68
HR (bpm)	82.2 (18.6)	81.8 (18.3)	83.3 (18.9)	81.9 (20.1)	79.1 (18.0)	82.6 (22.2)	82.4 (18.3)	91.9 (26.7)	82.2 (18.1)	0.21
Temperature	36.4 (0.6)	36.6 (0.6)	36.5 (0.6)	36.5 (0.6)	36.5 (0.6)	36.6 (0.6)	36.5 (0.7)	36.6 (0.6)	36.3 (0.7)	0.012
Glucose (mmol/l)	7.5 (3.3)	7.5 (3.0)	7.1 (2.6)	7.8 (4.2)	5.8 (1.0)	8.1 (3.3)	7.0 (2.3)	6.9 (3.0)	7.7 (4.0)	0.18
Diagnosis (%)										0.05
ICH	145 (12.6)	23 (12.9)	25 (11.5)	19 (9.4)	1 (14.3)	39 (14.7)	9 (10.1)	4 (10.8)	25 (16.3)	
Ischaemic	597 (52.0)	89 (50.0)	122 (56.0)	89 (44.1)	5 (71.4)	141 (53.2)	50 (56.2)	20 (54.1)	81 (52.9)	
TIA	109 (9.5)	14 (7.9)	21 (9.6)	21 (10.4)	0 (0.0)	28 (10.6)	12 (13.5)	2 (5.4)	11 (7.2)	
Mimic	298 (25.9)	52 (29.2)	50 (22.9)	73 (36.1)	1 (14.3)	57 (21.5)	18 (20.2)	11 (29.7)	36 (23.5)	

† Protocol violation

SBP: systolic blood pressure; DBP: diastolic blood pressure; HR: heart rate; bpm: beats per minute; E&W: England & Wales; EEAS – East of England Ambulance Service NHS Trust; EMAS – East Midlands Ambulance Service NHS Trust; FAST: Face arm speech test; GCS: Glasgow coma scale; ICH: intracerebral haemorrhage; LAS – London Ambulance Service; mRS: modified Rankin Scale (premorbid); OTR: onset to randomisation; SCAS – South Central Ambulance Service NHS Foundation Trust; SWAS – South Western Ambulance Service NHS Foundation Trust; TIA: transient ischaemic attack; WAS – Welsh Ambulance Service NHS Trust; YAS – Yorkshire Ambulance Service NHS Trust.

Multiple comparison procedure: Temperature - YAS differs from SWAS (p=0.002) and EEAS (p=0.002)

For peer review only

For peer review only - http://bmjopen.bmj.com/site/about/guidelines.xhtml

SUPPLEMENTAL TABLE III. Univariate correlations between baseline characteristics for age, sex, heart rate, systolic blood pressure and time from onset of symptoms to randomisation. Data are Spearman or point-biserial correlation coefficient (p-value).

	Sex	HR	SBP	Glucose	OTR	FAST	GCS
Age	-0.123 (<0.001)	-0.111 (<0.001)	0.076 (0.010)	0.140 (<0.001)	0.072 (0.15)	-0.004 (0.90)	-0.158 (<0.001)
Sex		-0.094 (0.001)	-0.069 (0.019)	-0.009 (0.76)	0.037 (0.21)	0.002 (0.95)	0.009 (0.76)
HR			0.119 (<0.001)	0.069 (0.019)	-0.012 (0.67)	0.007 (0.80)	0.006 (0.85)
SBP				0.26 (1.00)	0.005 (0.86)	-0.075 (0.011)	0.044 (0.14)
Glucose					0.023 (0.44)	0.059 (0.45)	-0.77 (0.009)
OTR						-0.137 (<0.001)	0.102 (0.001)
FAST							-0.157 (<0.001)

HR: heart rate; OTR: onset to randomisation; SBP: systolic blood pressure

For peer review only

For peer review only - http://bmjopen.bmj.com/site/about/guidelines.xhtml

SUPPLEMENTAL TABLE IV. Timings (in minutes) by ambulance service

Data are median [interquartile range] (minimum-maximum). Comparisons by Kruskal-Wallis and multiple comparison procedures (between groups) using Dunn's test with Bonferroni correction.

		E&W	EEAS	EMAS	LAS	SCAS	SWAS	WAS	WMAS	YAS	2p
Patients (N)		1149	178	218	202	7	265	89	37	153	-
Onset †	999 Call	19 [5, 64] (-216, 920)	18 [6, 69] (-58, 183)	15 [4, 57] (-89, 661)	25 [7, 76] (7, 76)	24 [11, 48] (1, 776)	18 [4, 55] (-216, 835)	14 [4, 46] (-45, 199)	27 [7, 75] (-25, 206)	20 [8, 65] (-10, 514)	0.36
999 Call	Dispatch	3 [1, 7] (0, 158)	1 [0, 4] (0, 115)	2 [1, 4] (0, 73)	4 [2, 8] (0, 78)	0 [0, 1] (0, 3)	5 [3, 14] (0, 131)	1 [0, 11] (0, 158)	2 [1, 3] (0,17)	2 [1, 7] (0, 55)	<0.001
Onset †	Arrive Scene (R1)	40 [21, 84] (-124, 928)	39 [19, 85] (-40, 205)	30 [15, 74] (-75, 691)	45 [19, 95] (-21, 928)	35 [33, 56] (11, 792)	45 [25, 79] (-124, 838)	28 [25, 85] (-19, 207)	39 [20, 95] (-14, 214)	40 [24, 84] (5, 523)	0.04
Onset †	Arrive Scene (R2)	44 [23, 86] (-124, 928)	43 [21, 88] (-40, 206)	35 [19, 80] (-75, 691)	50 [22, 97] (-75, 928)	35 [33, 56] (11, 792)	58 [25, 83] (-124, 838)	40 [25, 87] (-17, 207)	39 [20, 95] (-14, 214)	42 [25, 92] (5, 423)	0.23
Dispatch	Arrive Scene (R1)	8 [5, 13] (-31, 61)	8 [4, 14] (-26, 61)	7 [4, 12] (-31, 36)	6 [4, 9] (0, 28)	11 [7, 15] (6, 22)	10 [5, 17] (-18, 48)	12 [6, 17] (-9, 40)	9 [5, 13] (0, 39)	9 [6, 23] (-31, 39)	<0.001
Dispatch	RIGHT-2 paramedic	10 [6, 16] (0, 75)	10 [6, 19] (1, 61)	8 [5, 15] (0, 53)	8 [5, 13] (0, 65)	11 [7, 15] (6, 22)	11 [6, 18] (0, 75)	12 [7, 18] (0, 72)	9 [6, 14] (0, 39)	9 [6, 13] (-31, 39)	<0.001
Onset	Randomisation	71 [45, 116] (4, 942)	73 [47, 120] (11, 250)	59 [35, 100] (4, 720)	77 [51, 124] (15, 942)	53 [45, 65] (19, 811)	75 [49, 107] (6, 850)	75 [48, 123] (11, 395)	60 [32, 115] (17, 225)	70 [45, 118] (15, 535)	<0.001
Onset	Treatment	72 [48, 117] (4, 942)	73 [49, 73] (11, 251)	60 [37, 104] (4, 720)	77 [52, 124] (15, 942)	59 [50, 75] (19, 816)	78 [50, 109] (6, 874)	77 [48, 120] (11, 230)	65 [35, 118] (22, 229)	71 [48, 119] (22, 535)	0.005
RIGHT-2 Paramedic arrival †	Consent	19 [12, 29] (-17, 128)	20 [15, 28] (-1, 91)	15 [9, 22] (-5, 76)	25 [18, 33] (1, 73)	8 [5, 18] (2, 29)	20 [13, 30] (-10, 128)	23 [14, 32] (0, 87)	16 [9, 21] (-9, 46)	17 [10, 25] (-17, 65)	<0.001
RIGHT-2 Paramedic arrival †	Randomisation	22 [15, 31] (-34, 130)	23 [18, 32] (1, 95)	17 [11, 27] (-34, 80)	26 [19, 33] (1, 73)	11 [8, 19] (5, 30)	22 [16, 32] (-10, 130)	25 [18, 35] (0, 87)	17 [12, 24] (-8, 46)	19 [12, 27] (-13, 71)	<0.001
Consent	Randomisation	1 [0, 4] (0, 30)	2 [0, 4] (0, 30)	1 [0, 4] (0, 24)	0 [0, 0] (0, 18)	1 [0, 5] (0, 9)	0 [0, 4] (0, 20)	2 [0, 5] (0, 29)	1 [0, 3] (0, 10)	1 [0, 4] (0, 23)	<0.001
Arrive Scene	Depart scene	33 [26, 46] (0, 224)	38 [29, 49] (15, 224)	33 [25, 44] (10, 94)	31 [25, 38] (8, 72)	29 [23, 40] (10, 41)	43 [34, 87] (0, 162)	38 [28, 51] (15, 114)	31 [25, 38] (16, 46)	32 [24, 43] (0, 84)	<0.001
Onset	Hospital	97 [71, 141] (26, 953)	103 [73, 149] (29, 295)	87 [65, 129] (26, 748)	95 (68, 144] (32, 953)	90 [81, 115] (55, 841)	106 [77, 141] (32, 889)	109 [78, 153] (39, 430)	86 [58, 150] (31, 256)	92 [70, 131] (38, 545)	0.008
Randomisation	Treatment	0 [0, 2] (0, 57)	0 [0, 3] (0, 20)	0 [0, 2] (0, 57)	0 [0, 0] (0, 9)	5 [0, 8] (0, 9)	1 [0,4] (0, 27)	0 [0, 2] (0, 19)	1 [0, 4] (0, 24)	0 [0, 2] (0, 22)	<0.001

For peer review only - http://bmjopen.bmj.com/site/about/guidelines.xhtml

Randomisation	Hospital	24 [16, 34] (-13, 229)	25 [18, 33] (2, 229)	26 [17, 35] (-13, 92)	14 [10, 21] (0, 52)	35 [30, 36] (25, 62)	29 [21, 39] (4, 85)	30 [19, 42] (-1, 92)	22 [19, 28] (6, 48)	23 [16, 29 (-3, 86)]	<0.001
Depart scene	Hospital	15 [10, 23] (0, 98)	17 [10, 23] (1, 47)	14 [9, 23] (0, 50)	13 [10, 19] (2, 49)	24 [14, 25] (4, 33)	20 [12, 29] (0, 98)	16 [10, 28] (0, 56)	9 [7, 13] (-3, 27)	12 [8, 17] (0, 45)	<0.001

† Negative times: paramedic already at scene

E&W: England & Wales; EEAS – East of England Ambulance Service NHS Trust; EMAS – East Midlands Ambulance Service NHS Trust; LAS – London Ambulance Service; R1: First resource; R2: RIGHT-2 trained paramedic (if not on first resource) – two ambulance services permitted single responder paramedics to participate, otherwise R2 trained paramedics arrive on double-crewed ambulances; SCAS – South Central Ambulance Service NHS Foundation Trust; SWAS – South Western Ambulance Service NHS Foundation Trust; WAS – Welsh Ambulance Service NHS Trust; YAS – Yorkshire Ambulance Service NHS Trust.

Results of multiple comparison testing are given in the Supplemental material.

For peer review only

SUPPLEMENTAL TABLE V. Timings: Onset of symptoms to 999 call. Data are N (%), median [25, 75 centile]; comparison by Kruskal-Wallis test.

	E&W	EEAS	EMAS	LAS	SCAS	SWAS	WAS	WMAS	YAS	2p
Minutes<br>N (%)<br>Median [25, 75 centile]	1149<br>19 [5, 64]	178 (15.5)<br>18 [6, 69]	218 (19.0)<br>15 [4, 57]	202 (17.6)<br>25 [7, 76]	7 (0.6)<br>24 [11, 48]	265 (23.1)<br>18 [4, 55]	89 (7.7)<br>14 [4, 46]	37 (3.2)<br>27 [7, 75]	153 (13.3)<br>20 [8, 65]	0.36
N (%)										0.012
<10	430 (37.4)	67 (37.6)	94 (43.1)	68 (33.7)	1 (14.3)	102 (38.5)	40 (44.9)	12 (32.4)	46 (30.1)	
11-20	169 (14.7)	26 (14.6)	29 (13.3)	27 (13.4)	1 (14.3)	39)14.7)	11 912.4)	4 (10.8)	32 (20.9)	
21-30	84 (7.3)	9 (5.1)	18 (8.3)	14 (6.9)	2 (28.6)	23 (8.7)	3 (3.4)	3 (8.1)	12 (7.8)	
31-60	164 (14.3)	23 (12.9)	25 (11.5)	29 (14.4)	2 (28.6)	43 (16.2)	16 (18.0)	5 (13.5)	21 (13.7)	
61-240	290 (25.2)	53 (29.8)	50 (22.9)	58 (28.7)	0 (0.0)	57 (21.5)	18 (20.2)	13 (35.1)	41 (26.8)	
>240 †	11 (1.0)	0 (0.0)	2 (0.9)	6 (3.0)	1 (14.3)	1 (0.4)	88 (98.9)	0 (0.0)	1 (0.7)	

† >240 minutes is protocol violation, typically due to wake-up stroke or uncertainty of onset time.

For peer review only

SUPPLEMENTAL TABLE VI. Comparison of EMAS stroke patients in RIGHT-2 versus non-RIGHT-2. Data are median of time (min) [25, 75 centile] or distance km [25, 75 centile]

	RIGHT-2	Non-RIGHT-2	2p
Patients (N)	218	49	
Time			
Symptom onset – 999 call	15 [5, 57]	†	
999 call – dispatch	2 [1, 5]	2 [1, 3]	0.48
999 call – scene arrival	10 [7, 16]	12 [8, 18]	0.17
Time on scene	34 [26, 44]	32 [23, 41]	0.12
Scene arrival - hospital			
Scene departure – hospital	14 [9, 22]	17 [12, 25]	0.18
Symptom onset – hospital	86 [65, 128]	†	
999 call - hospital	63 [48, 76]	62 [49, 82]	0.80
Distance			
Dispatch - Scene	7.3 [3.5, 12.0]	9.6 [3.6, 16.7]	0.23
Scene - hospital	10.0 [0.4, 64.7]	15.9 [7.6, 24]	0.011

† Symptom onset time not available

For peer review only

SUPPLEMENTAL TABLE VII. Timings: Symptom onset to arrival at hospital (minutes). Data are N (%), median [25, 75 centile]; comparison by Kruskal-Wallis test.

Minutes	E&W	EEAS	EMAS	LAS	SCAS	SWAS	WAS	WMAS	YAS	2p
N (%)	1149	178	218	202	7	265	89	37	153	
Median [25, 75 centile]	97 [71, 141]	103 [73, 149]	87 [65, 129]	95 (68, 144]	90 [81, 115]	106 [77, 141]	109 [78, 153]	86 [58, 150]	92 [70, 131]	0.008
N(%)										0.040
<30	6 (0.5)	1 (0.6)	5 (2.3)	0 (0.0)	0 (0.0)	0 (0.0)	0 (0.0)	0 (0.0)	0 (0.0)	
31-60	157 (13.7)	15 (8.4)	39 (17.9)	37 (18.3)	1 (14.3)	24 (9.1)	9 (10.1)	10 (27.0)	22 (14.4)	
61-90	350 (30.5)	61 (34.3)	70 (32.1)	54 (26.7)	3 (42.9)	75 (28.3)	23 (25.8)	10 (27.0)	54 (35.3)	
91-120	227 (19.8)	29 (16.3)	40 (18.3)	39 (19.3)	2 (28.6)	62 (23.4)	20 (22.5)	5 (13.5)	30 (19.6)	
121-240	371 (32.3)	68 (38.2)	58 (26.6)	64 (31.7)	0 (0.0)	96 (36.2)	34 (38.2)	11 (29.7)	40 (26.1)	
>240	38 (3.3)	4 (2.2)	6 (2.8)	8 (4.0)	1 (14.3)	8 (3.0)	3 (3.4)	1 (2.7)	7 (4.6)	

For peer review only

For peer review only - http://bmjopen.bmj.com/site/about/guidelines.xhtml

SUPPLEMENTAL TABLE VIII. Conveyance distances (kilometres). Data are median of distance (minimum-maximum). Comparison by Kruskal-Wallis test and multiple comparison procedure. One ambulance service was unable to provide location data.

	E&W	EEAS	EMAS	SCAS	SWAS	WAS	WMAS	YAS	2p
N (%)	936	178	213	7	263	87	37	152	
Median	10.0	12.3	9.4	19.9	13.6	12.1	4.1	6.4	<0.001
(min, max)	[4.4, 18.4]	[4.5, 20.9]	[4.4, 19.1]	[2.7, 19.8]	[6.2, 20.1]	[4.7, 23.8]	[3.4, 10.2]	[3.3, 10.8]	
	(0.4, 64.7)	(0.6, 34.0)	(1.1, 59.9)	(1.9, 22.4)	(0.6, 51.3)	(0.6, 64.7)	(0.9, 28.8)	(0.4, 44.3)	
N (%)									<0.001
<5 Km	273 (29.2)	49 (27.7)	66 (31.0)	2 (28.6)	49 (18.6)	20 (23.2)	23 (62.2)	64 (42.1)	
5-10 km	193 (20.6)	25 (14.1)	42 (19.7)	0 (0.0)	61 (23.2)	18 (20.7)	4 (10.8)	43 (28.3)	
10.1-15 km	142 (15.2)	27 (15.3)	31 (14.6)	1 (14.3)	41 (15.6)	13 (14.9)	5 (13.5)	24 (15.8)	
15.1-20 km	112 (12.0)	22 (12.4)	26 (12.2)	1 (14.2)	44 (16.7)	10 (11.5)	3 (8.1)	6 (3.9)	
20.1-25 km	107 (11.4)	34 (19.2)	25 (11.7)	3 (42.9)	28 (10.6)	10 (11.5)	1 (2.7)	6 (3.9)	
>=25 km	109 (11.6)	20 (11.3)	23 (10.8)	0 (0.0)	40 (15.2)	16 (18.4)	1 (2.7)	9 (5.9)	

Multiple Comparison testing:
WMAS differs from EMAS p=0.016, EEAS p=0.001, SWAS p<0.001 and WAS p=0.001
YAS differs from EMAS p=0.002, EEAST p<0.001, SWAS p<0.001 and WAS p<0.001

For peer review only

SUPPLEMENTAL FIGURE I. Distribution of participants by Ambulance Service

A) East Midlands Ambulance Service NHS Trust

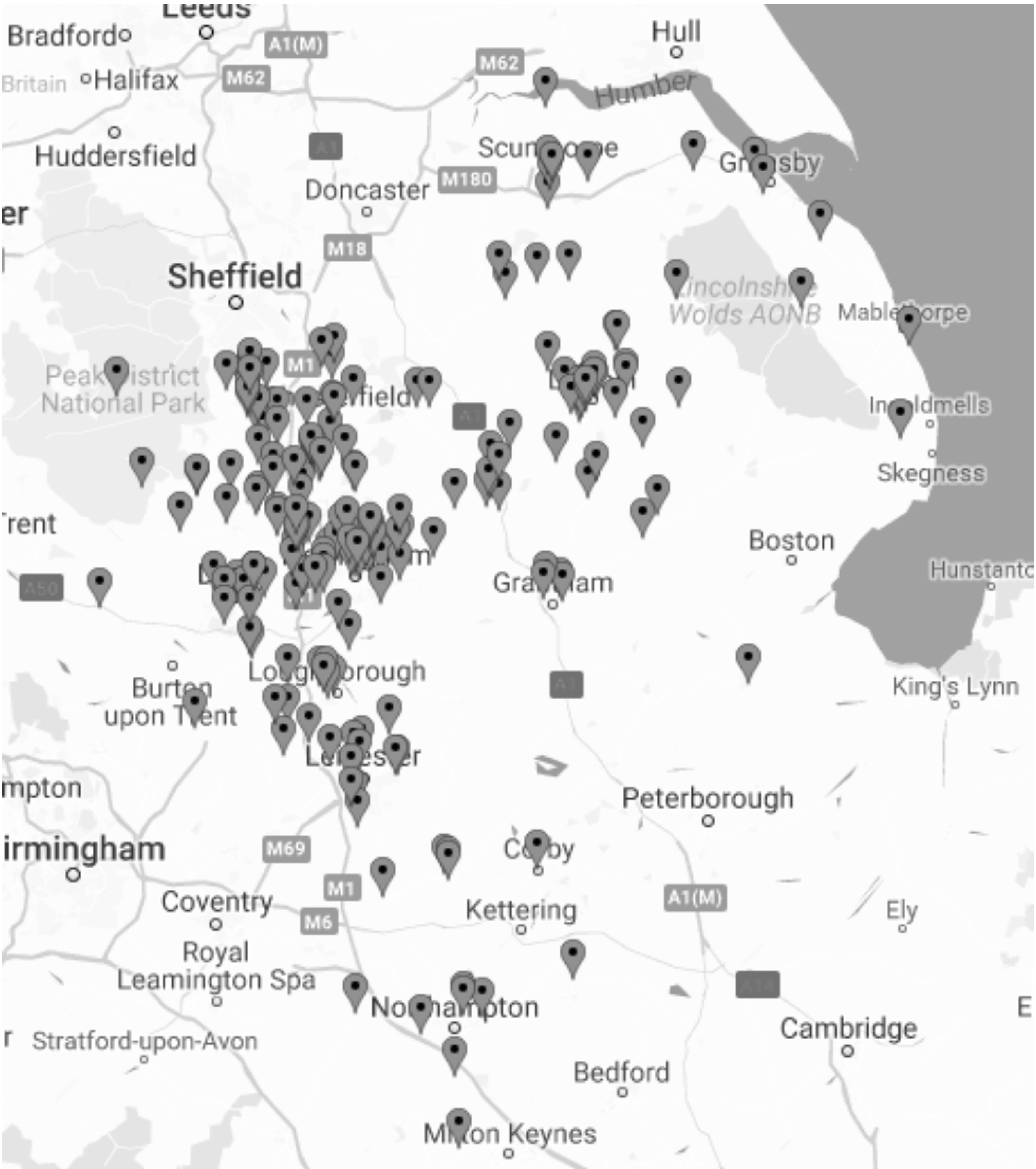

B) East of England Ambulance Service NHS Trust

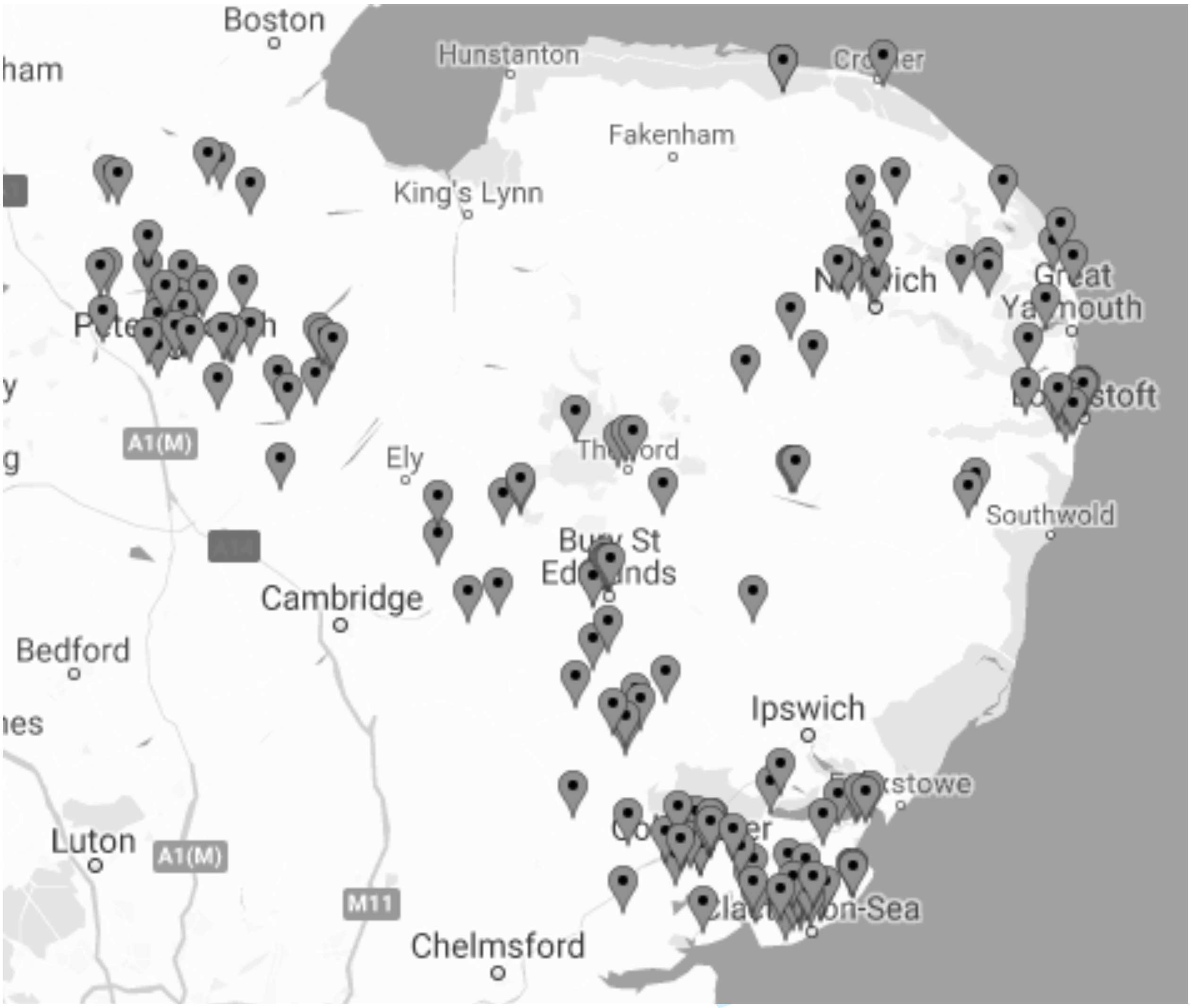

only

For peer review only - http://bmjopen.bmj.com/site/about/guidelines.xhtml

C) South Central Ambulance Service NHS Foundation Trust

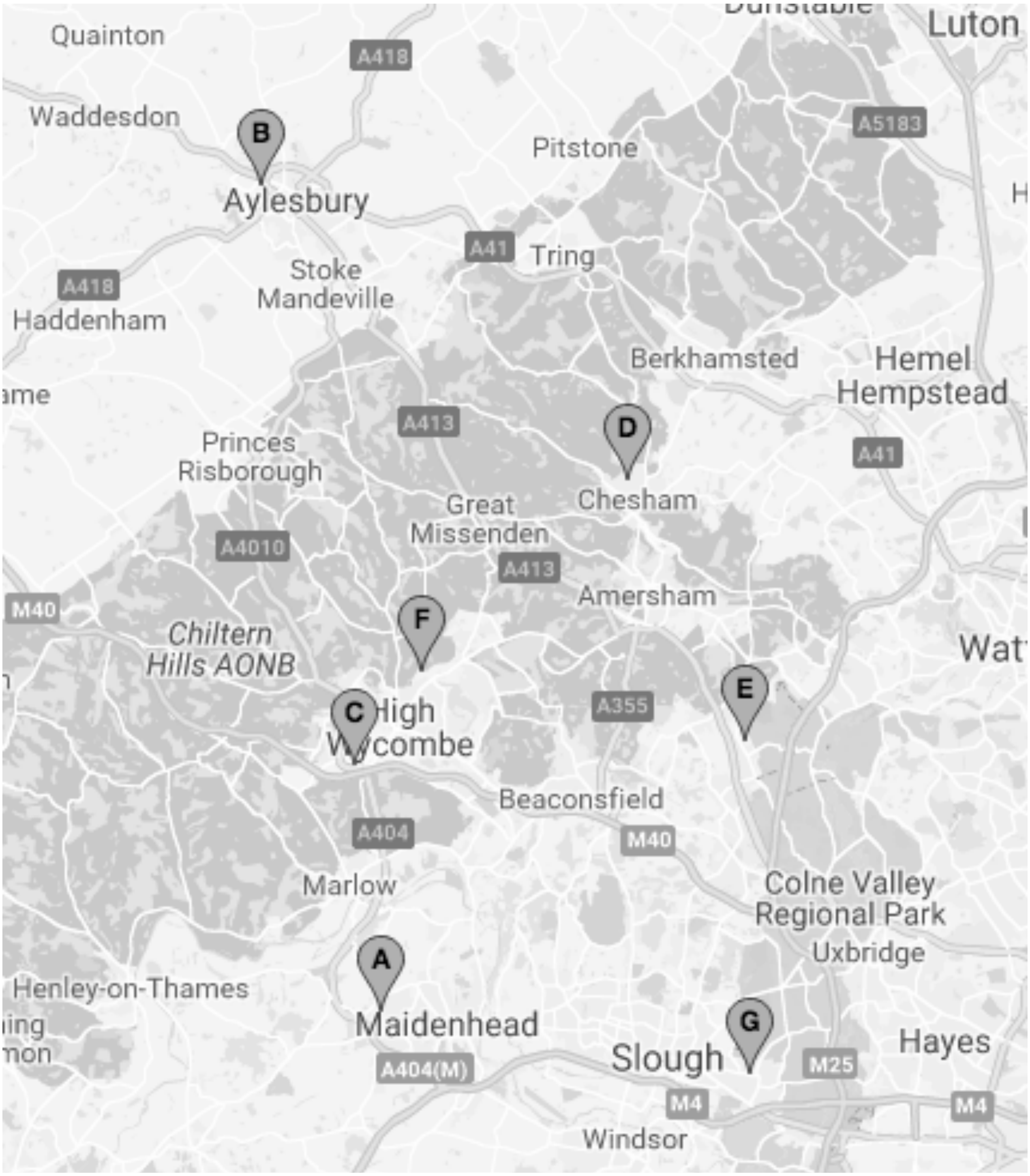

D) South Western Ambulance Service NHS Foundation Trust

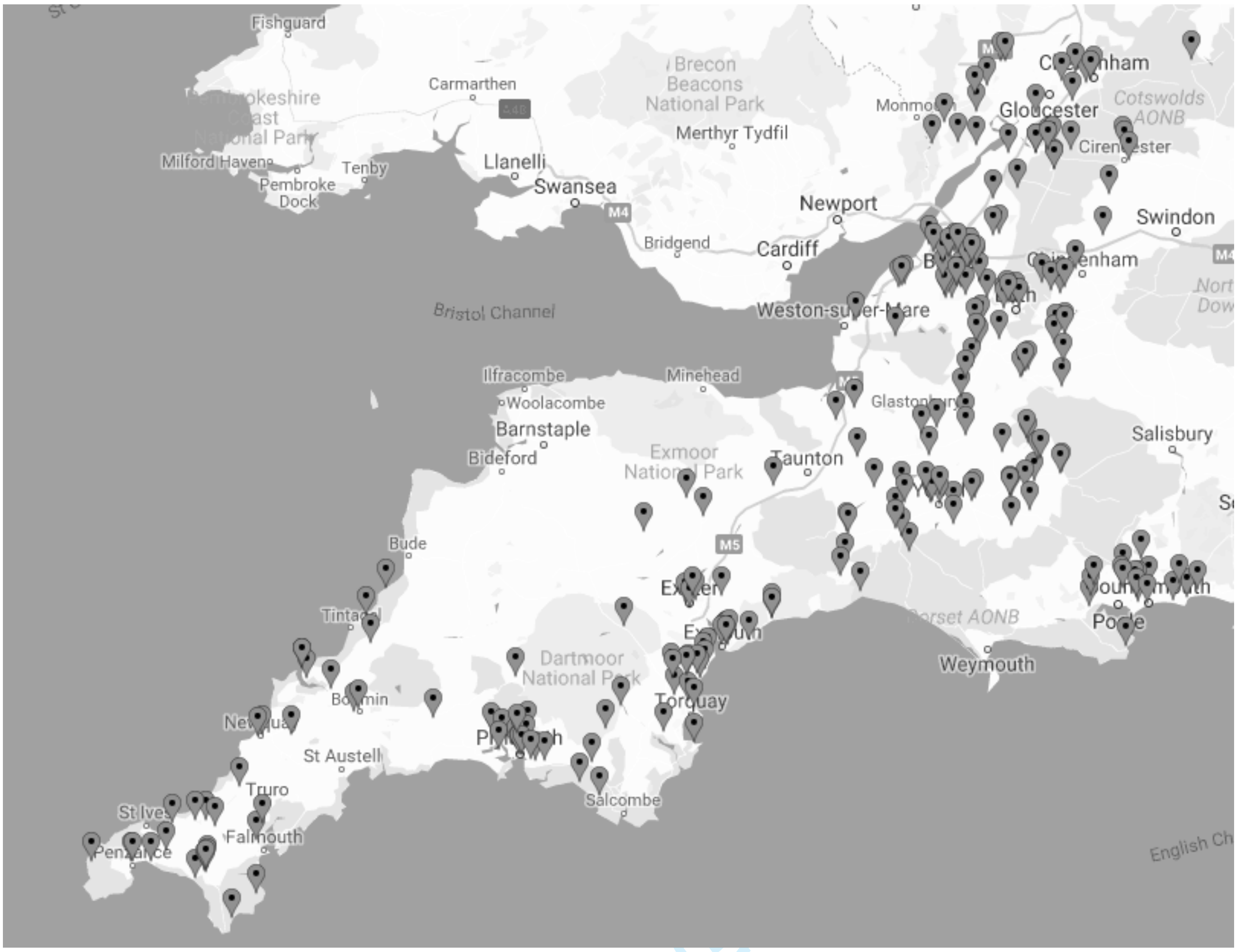

E) Welsh Ambulance Service NHS Trust

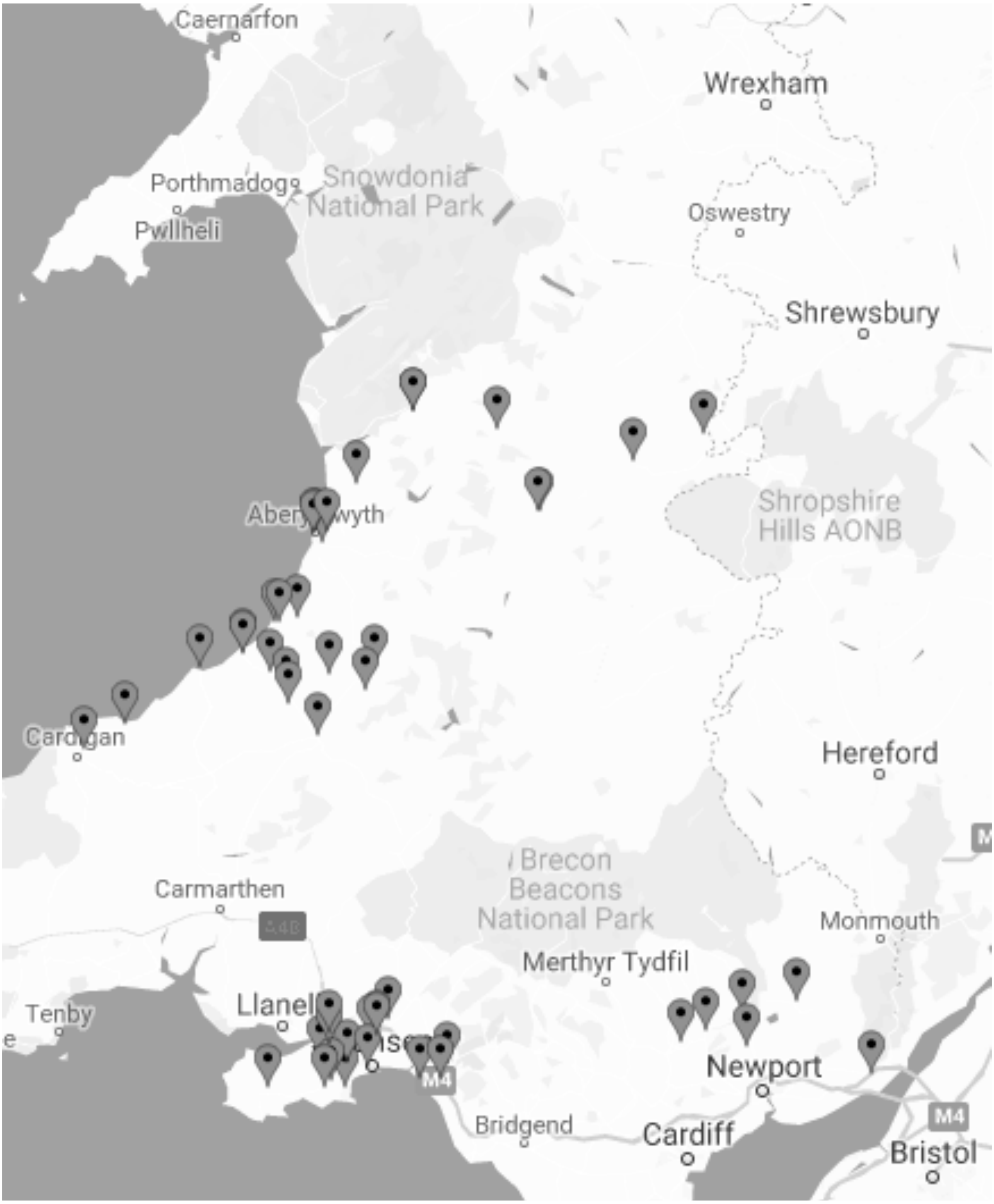

F) West Midlands Ambulance Service University NHS Foundation Trust

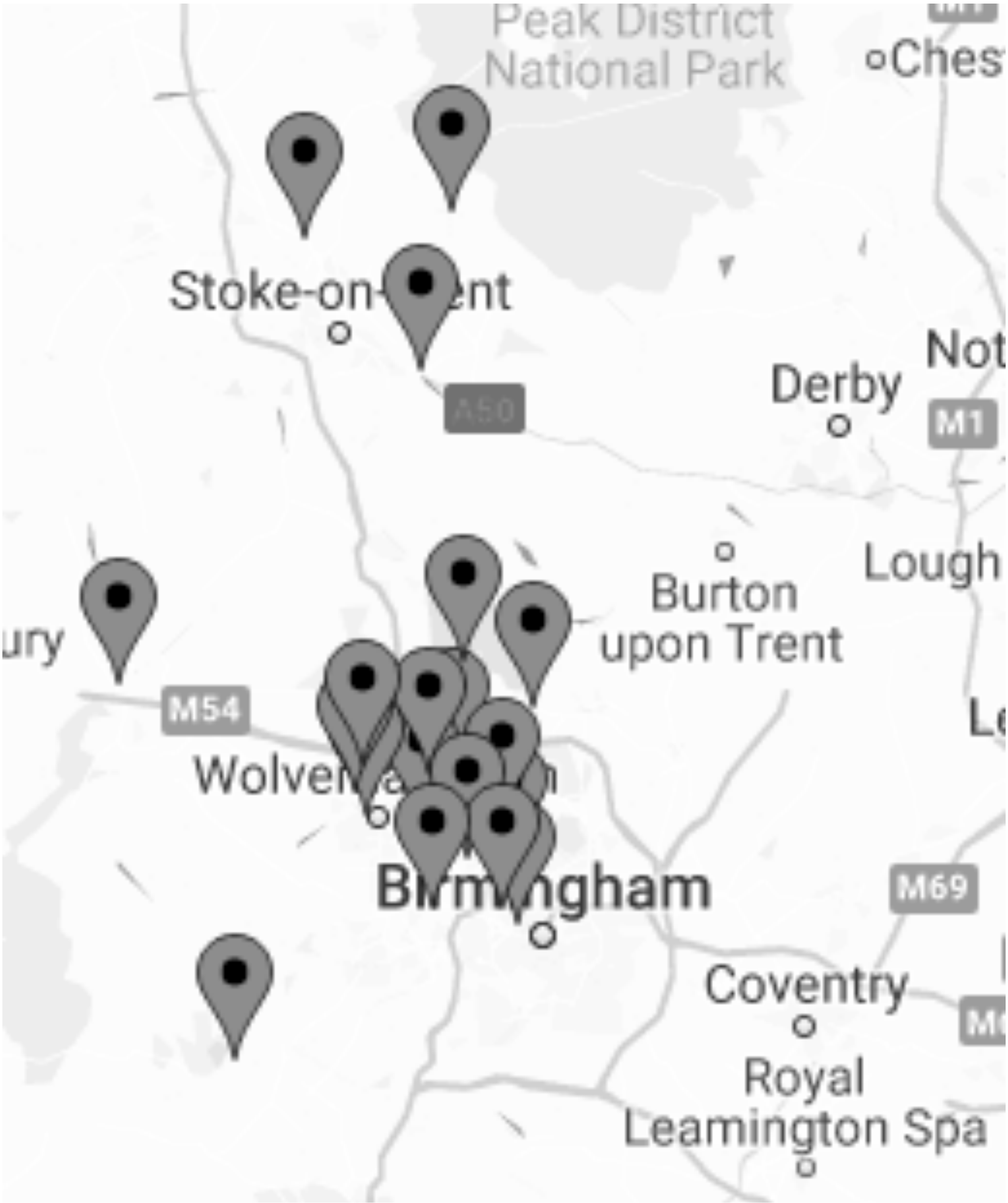

G) Yorkshire Ambulance Service NHS Trust

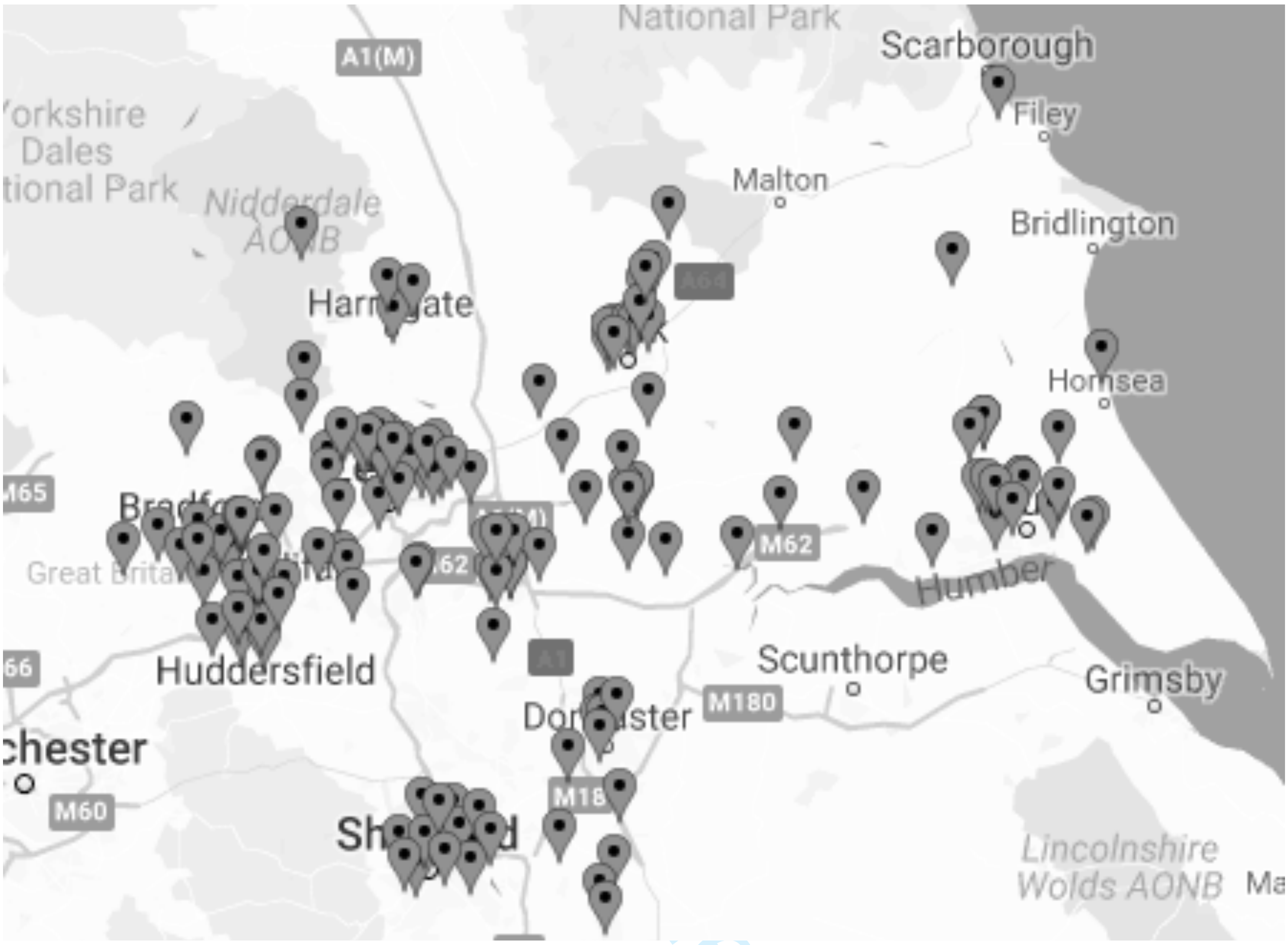

For peer review only - http://bmjopen.bmj.com/site/about/guidelines.xhtml

SUPPLEMENTAL REFERENCES

1. Office for National Statistics. 2011 Census Analysis - Comparing Rural and Urban Areas of England and Wales: Office for National Statistics, 2013.
2. Appleton JP, Scutt P, Dixon M, et al. Ambulance-delivered transdermal glyceryl trinitrate versus sham for ultra-acute stroke: Rationale, design and protocol for the Rapid Intervention with Glyceryl trinitrate in Hypertensive stroke Trial-2 (RIGHT-2) trial (ISRCTN26986053). *Int J Stroke* 2017;0(0):1-16. doi: 10.1177/1747493017724627

For peer review only

CONSORT 2010 checklist of information to include when reporting a randomised trial*

Section/Topic	Item No	Checklist item	Reported on page No
**Title and abstract**			
	1a	Identification as a randomised trial in the title	1
	1b	Structured summary of trial design, methods, results, and conclusions (for specific guidance see CONSORT for abstracts)	5
**Introduction**			
Background and objectives	2a	Scientific background and explanation of rationale	4
	2b	Specific objectives or hypotheses	4
**Methods**			
Trial design	3a	Description of trial design (such as parallel, factorial) including allocation ratio	5
	3b	Important changes to methods after trial commencement (such as eligibility criteria), with reasons	
Participants	4a	Eligibility criteria for participants	5
	4b	Settings and locations where the data were collected	5
Interventions	5	The interventions for each group with sufficient details to allow replication, including how and when they were actually administered	5
Outcomes	6a	Completely defined pre-specified primary and secondary outcome measures, including how and when they were assessed	5/6
	6b	Any changes to trial outcomes after the trial commenced, with reasons	n/a
Sample size	7a	How sample size was determined	5 (refers to previous protocol publication)
	7b	When applicable, explanation of any interim analyses and stopping guidelines	5 (refers to previous protocol publication)
Randomisation:			
Sequence generation	8a	Method used to generate the random allocation sequence	5 (refers to previous

For peer review only - http://bmjopen.bmj.com/site/about/guidelines.xhtml

			protocol publication)
	8b	Type of randomisation; details of any restriction (such as blocking and block size)	5 (refers to previous protocol publication)
Allocation concealment mechanism	9	Mechanism used to implement the random allocation sequence (such as sequentially numbered containers), describing any steps taken to conceal the sequence until interventions were assigned	5 (refers to previous protocol publication)
Implementation	10	Who generated the random allocation sequence, who enrolled participants, and who assigned participants to interventions	5 (refers to previous protocol publication)
Blinding	11a	If done, who was blinded after assignment to interventions (for example, participants, care providers, those assessing outcomes) and how	5 (refers to previous protocol publication)
	11b	If relevant, description of the similarity of interventions	5 (refers to previous protocol publication)
Statistical methods	12a	Statistical methods used to compare groups for primary and secondary outcomes	5 / 6
	12b	Methods for additional analyses, such as subgroup analyses and adjusted analyses	6
**Results**			
Participant flow (a diagram is strongly recommended)	13a	For each group, the numbers of participants who were randomly assigned, received intended treatment, and were analysed for the primary outcome	8
	13b	For each group, losses and exclusions after randomisation, together with reasons	n/a
Recruitment	14a	Dates defining the periods of recruitment and follow-up	8
	14b	Why the trial ended or was stopped	Available in main RIGHT-2 results paper

For peer review only

For peer review only - http://bmjopen.bmj.com/site/about/guidelines.xhtml

			(Lancet, 2019)
Baseline data	15	A table showing baseline demographic and clinical characteristics for each group	22
Numbers analysed	16	For each group, number of participants (denominator) included in each analysis and whether the analysis was by original assigned groups	Available in main RIGHT-2 results paper (Lancet, 2019)
Outcomes and estimation	17a	For each primary and secondary outcome, results for each group, and the estimated effect size and its precision (such as 95% confidence interval)	Available in main RIGHT-2 results paper (Lancet, 2019)
	17b	For binary outcomes, presentation of both absolute and relative effect sizes is recommended	Available in main RIGHT-2 results paper (Lancet, 2019)
Ancillary analyses	18	Results of any other analyses performed, including subgroup analyses and adjusted analyses, distinguishing pre-specified from exploratory	8 / 9
Harms	19	All important harms or unintended effects in each group (for specific guidance see CONSORT for harms)	Available in main RIGHT-2 results paper (Lancet, 2019)
**Discussion**			
Limitations	20	Trial limitations, addressing sources of potential bias, imprecision, and, if relevant, multiplicity of analyses	11
Generalisability	21	Generalisability (external validity, applicability) of the trial findings	12
Interpretation	22	Interpretation consistent with results, balancing benefits and harms, and considering other relevant evidence	10 / 11

For peer review only - http://bmjopen.bmj.com/site/about/guidelines.xhtml

**Other information**			
Registration	23	Registration number and name of trial registry	3
Protocol	24	Where the full trial protocol can be accessed, if available	5
Funding	25	Sources of funding and other support (such as supply of drugs), role of funders	3 / 13

*We strongly recommend reading this statement in conjunction with the CONSORT 2010 Explanation and Elaboration for important clarifications on all the items. If relevant, we also recommend reading CONSORT extensions for cluster randomised trials, non-inferiority and equivalence trials, non-pharmacological treatments, herbal interventions, and pragmatic trials. Additional extensions are forthcoming: for those and for up to date references relevant to this checklist, see www.consort-statement.org.

For peer review only

BMJ Open

Time intervals and distances travelled for pre-hospital ambulance stroke care: data from the randomised-controlled ambulance-based Rapid Intervention with Glyceryl trinitrate in Hypertensive stroke Trial-2 (RIGHT-2)

Journal:	*BMJ Open*
Manuscript ID	bmjopen-2021-060211.R1
Article Type:	Original research
Date Submitted by the Author:	18-Aug-2022

Complete List of Authors:	Dixon, Mark; University of Nottingham Faculty of Medicine and Health Sciences, Division of Mental Health and Clinical Neuroscience; East Midlands Ambulance Service NHS Trust,<br>Appleton, Jason; University Hospitals Birmingham NHS Foundation Trust<br>Scutt, Polly; University of Nottingham Faculty of Medicine and Health Sciences, Division of Mental Health and Clinical Neuroscience<br>Woodhouse, Lisa; University of Nottingham Faculty of Medicine and Health Sciences, Division of Mental Health and Clinical Neuroscience<br>Haywood, Lee; University of Nottingham Faculty of Medicine and Health Sciences, Division of Mental Health and Clinical Neuroscience<br>Havard, Diane; University of Nottingham Faculty of Medicine and Health Sciences, Division of Mental Health and Clinical Neuroscience<br>Williams, Julia; University of Hertfordshire, Division of Paramedic Science, School of Health and Social Work<br>Siriwardena, Aloysius; University of Lincoln, School of Health and Social Care<br>Bath, Philip; University of Nottingham Faculty of Medicine and Health Sciences, Division of Mental Health and Clinical Neuroscience<br>RIGHT-2 Investigators, ,; University of Nottingham Faculty of Medicine and Health Sciences, Division of Mental Health and Clinical Neuroscience

**Primary Subject Heading**:	Neurology
Secondary Subject Heading:	Emergency medicine
Keywords:	Stroke < NEUROLOGY, ACCIDENT & EMERGENCY MEDICINE, Stroke medicine < INTERNAL MEDICINE

SCHOLARONE™ Manuscripts

I, the Submitting Author has the right to grant and does grant on behalf of all authors of the Work (as defined in the below author licence), an exclusive licence and/or a non-exclusive licence for contributions from authors who are: i) UK Crown employees; ii) where BMJ has agreed a CC-BY licence shall apply, and/or iii) in accordance with the terms applicable for US Federal Government officers or employees acting as part of their official duties; on a worldwide, perpetual, irrevocable, royalty-free basis to BMJ Publishing Group Ltd ("BMJ") its licensees and where the relevant Journal is co-owned by BMJ to the co-owners of the Journal, to publish the Work in this journal and any other BMJ products and to exploit all rights, as set out in our licence.

The Submitting Author accepts and understands that any supply made under these terms is made by BMJ to the Submitting Author unless you are acting as an employee on behalf of your employer or a postgraduate student of an affiliated institution which is paying any applicable article publishing charge ("APC") for Open Access articles. Where the Submitting Author wishes to make the Work available on an Open Access basis (and intends to pay the relevant APC), the terms of reuse of such Open Access shall be governed by a Creative Commons licence – details of these licences and which Creative Commons licence will apply to this Work are set out in our licence referred to above.

Other than as permitted in any relevant BMJ Author's Self Archiving Policies, I confirm this Work has not been accepted for publication elsewhere, is not being considered for publication elsewhere and does not duplicate material already published. I confirm all authors consent to publication of this Work and authorise the granting of this licence.

For peer review only

Time intervals and distances travelled for pre-hospital ambulance stroke care: data from the randomised-controlled ambulance-based Rapid Intervention with Glyceryl trinitrate in Hypertensive stroke Trial-2 (RIGHT-2)

Mark Dixon, paramedic divisional senior manager (quality);[1,2] Jason P Appleton, consultant neurologist;[1,3] Polly Scutt, medical statistician;[1] Lisa J Woodhouse, medical statistician;[1] Lee J Haywood, database programmer;[1] Diane Havard, senior trial manager;[1] Julia Williams, Professor of paramedic science;[4] A Niroshan Siriwardena, Professor of primary and prehospital care;[5] Philip M Bath, Professor of stroke medicine;[1] on behalf of the *RIGHT-2 Investigators.*

[1]Stroke Trials Unit, Division of Mental Health and Clinical Neuroscience, University of Nottingham, Nottingham, UK.
[2]East Midlands Ambulance Service NHS Trust, Nottingham, UK.
[3]Stroke, University Hospitals Birmingham NHS Foundation Trust, Birmingham, UK.
[4]Division of Paramedic Science, School of Health and Social Work, University of Hertfordshire, Hatfield, UK.
[5]School of Health and Social Care, University of Lincoln, Lincoln, UK.

Corresponding author
Philip M Bath, Stroke Trials Unit, Division of Mental Health and Clinical Neuroscience, University of Nottingham, Queen's Medical Centre campus, Nottingham, NG7 2UH UK.
Email: philip.bath@nottingham.ac.uk
Tel: 0115 823 1765
Fax: 0115 823 1767

Word Count
3,359 (Excluding title page, abstract, references, figures and tables, authorship and contributorship statements)

References
42

For peer review only

ABSTRACT

Objectives

Ambulances offer the first opportunity to evaluate hyperacute stroke treatments. We investigated the conduct of a hyperacute stroke study in the ambulance-based setting with a particular focus on timings and logistics of trial delivery.

Design

Multicentre prospective, single-blind, parallel group randomised controlled trial.

Setting

Eight NHS ambulance services in England and Wales; 54 acute stroke centres.

Participants

Paramedics enrolled 1,149 patients with likely stroke, face, arm speech (2 or 3), within four hours of symptom onset and systolic BP>120mmHg.

Interventions

Paramedics administered randomly assigned active transdermal glyceryl trinitrate or sham.

Primary and Secondary Outcomes

Modified Rankin scale at day-90. This paper focuses on response time intervals, distances travelled and baseline characteristics of patients, compared between ambulance services.

Results

Paramedics enrolled 1,149 patients between September 2015 and May 2018. Final diagnosis: intracerebral haemorrhage 13%, ischaemic stroke 52%, TIA 9%, mimic 26%. Timings (minutes) were (median [25, 75 centile]): onset to emergency call 19 [5, 64]; onset to randomisation 71 [45, 116]; total time at scene 33 [26, 46]; depart scene to hospital 15 [10, 23]; randomisation to hospital 24 [16, 34] and onset to hospital 97 [71, 141]. Ambulances travelled (km) 10 [4, 19] from scene to hospital. Timings and distances differed between ambulance service, e.g. onset to randomisation (fastest 53, slowest 77 minutes; p<0.001), distance from scene to hospital (least 4, most 20 km; p<0.001).

For peer review only - http://bmjopen.bmj.com/site/about/guidelines.xhtml

For peer review only

Conclusion

We completed a large pre-hospital stroke trial involving a simple-to-administer intervention across multiple ambulance services. The time from onset to randomisation and modest distances travelled support the applicability of future large-scale paramedic-delivered ambulance-based stroke trials in urban and rural locations.

Registration

ISRCTN26986053

Abstract word count

269

Keywords

Ambulance, paramedic, stroke, glyceryl trinitrate, logistics

For peer review only

For peer review only - http://bmjopen.bmj.com/site/about/guidelines.xhtml

STRENGTHS AND LIMITATIONS

- The first multicentre paramedic-delivered ambulance-based randomised-controlled trial in stroke in the United Kingdom.
- Ambulance response time intervals and distances are collated and reported for 1,149 patients assessed as likely stroke.
- The time-interval between arrival at hospital and the ambulance becoming available for the next emergency call (hospital turnaround) is not captured, but worth considering for future trials
- Timing and logistic data may not be fully representative of all urban and rural locations due to non-participation of some hospitals and ambulance stations within ambulance service regional areas.

For peer review only

For peer review only - http://bmjopen.bmj.com/site/about/guidelines.xhtml

INTRODUCTION

Routine pre-hospital management of suspected acute stroke involves rapid identification of suspected stroke using a validated stroke screening tool, prompt transport, pre-arrival notification and primary stabilisation to the nearest appropriate receiving stroke centre.[1] The mainstays for hyperacute management of stroke in hospital include urgent neuroimaging, stroke unit care, reperfusion therapy for ischaemic stroke, and blood pressure (BP) lowering for intracerebral haemorrhage.[2] For reperfusion therapies, shortening the time from symptom onset to treatment improves functional outcome and this has become the aim of pre-hospital and in-hospital acute stroke services.[3-6] Thus, ambulance services play a crucial role in assessing, identifying and conveying patients with suspected stroke to primary and comprehensive stroke centres, which may include bypassing local emergency departments.

Timely pre-hospital care for stroke is dependent upon several factors that include rapid recognition of potential stroke and calling for help,[7] ambulance response times encompassing symptom onset to arrival at hospital,[8] distance from scene to hospital,[9] and the accuracy of identifying patients with true stroke or transient ischaemic attack (TIA) from those with a stroke mimic.[10] There are a small, but growing number of studies that explore randomised paramedic-initiated interventions commencing in the ambulance for acute stroke. However, few studies have systematically analysed these parameters and the factors that influence them in acute pre-hospital stroke practice.[4 11-13]

Here, we report the logistics underlying patient recruitment to the Rapid Intervention with Glyceryl trinitrate (GTN) in Hypertensive stroke Trial-2 (RIGHT-2), a large ambulance-based stroke trial in the UK that investigated the efficacy of transdermal glyceryl trinitrate as a paramedic-delivered intervention in suspected acute stroke. Specifically, ambulance response times and distance travelled across multiple organisations in this setting are assessed.

For peer review only - http://bmjopen.bmj.com/site/about/guidelines.xhtml

METHODS

RIGHT-2 trial

RIGHT-2 (registration ISRCTN26986053) commenced recruitment in September 2015 with the first participant recruited on 22 October 2015.

RIGHT-2 was a multicentre prospective, single-blind, parallel group randomised controlled trial; the protocol, statistical analysis plan, baseline data, main results and subgroup results in participants with a final diagnosis of intracerebral haemorrhage (ICH) are published.[14-18] Briefly, adult patients with suspected stroke presenting to the emergency service via an emergency call were recruited if they: were FAST-positive (facial weakness, arm weakness, speech abnormality; with test score 2 or 3), had systolic BP of >120mmHg, were within 4 hours of symptom onset, presented to trial-trained paramedics from eight UK ambulance services, and were to be taken to a trial-participating hospital. Patients were randomised to receive transdermal glyceryl trinitrate (GTN) or sham patch in the ambulance and this was continued for three further days during hospital admission.[19] The study was undertaken across eight UK ambulance services (AS): East of England AS (EEAS), East Midlands AS (EMAS), London AS (LAS), South-Central AS (SCAS), South-West AS (SWAS), Welsh AS (WAS), West-Midlands AS (WMAS) and Yorkshire AS (YAS). All participating ambulance services used FAST identification and protocols consistent with national guidelines.

For each eligible patient, the enrolling paramedic assessed capacity and obtained patient or proxy consent (from a relative on the scene, or from the paramedic witnessed by a colleague), completed a written case report form to capture in-ambulance baseline and on-treatment data, and applied the transdermal patch of GTN or sham dressing.[14] Ambulance-related data not recorded at source were confirmed by research paramedics from participating ambulance services after review of control room timing logs or patient care records, and then entered into the trial database.

Timings and distances

Timings were obtained from each ambulance service (time of emergency call, resource dispatch, scene arrival and departure, hospital arrival) and from paramedic records (consent for trial enrolment, randomisation, application of study treatment). Paramedic-documented history provided the time of symptom onset or, where

For peer review only - http://bmjopen.bmj.com/site/about/guidelines.xhtml

unclear, the last known well time.

Distance measurements were calculated from the address or postcode of the emergency location, where available, to the expected stopping point for the ambulance at the destination hospital (accident and emergency or stroke unit entrance) to the nearest 10 metres using Google Maps; one ambulance service was unable to provide postcode information due to time constraints. One ambulance service was able to provide the linear distance from the location of the ambulance at the point of dispatch to the scene of the emergency.

A comparison of urban versus rural ambulance services arbitrarily divided ASs by <25% rural versus >25% rural (as defined in Table 1; Supplemental Table I).

For peer review only

For peer review only - http://bmjopen.bmj.com/site/about/guidelines.xhtml

Table 1. Characteristics of participating ambulance service as of 31st May 2018. Data are numbers (%)

	E&W	EEAS	EMAS	LAS	SCAS	SWAS	WAS	WMAS	YAS
Time in trial (months)	32	27	32	14	4	27	22	14	29
Patients	1149	178	218	202	7	265	89	37	153
Participating hospitals	54	5	10	3	1	13	4	5	13
Area (km^2)	122,065	19,424	16,710	1,605	9,204	25,899	20,735	12,949	15,539
Population									
Overall (x1000)	53,000	5,800	4,800	8,600	7,000	5,300	2,900	5,600	5,338
Living in rural areas† (%)	17.6	28.9	26.7	0.2	20.4	31.6	32.8	15.1	17.5
Strokes (/yr) ††	90,781	9,145	9,246	13,118	7,763	10,442	7,400	8,701	7,931
Adjusted ratio /1000	1.71	1.58	1.92	1.52	1.11	1.97	2.55	1.55	1.49
Call volume (/day)	24,661	2,800	2,500	5,193	1,479	3,077	1,331	3,000	2,336
Participating Ambulance Stations	270	24	50	23	3	73	34	17	63
Paramedics employed	22,000	2,000	1,111	2,864	1,780	1,788	1,310	1,300	1,592
Trained in RIGHT-2	1,492	145	193	325	63	313	165	124	142
Paramedics who recruited	516	58	75	120	6	112	47	23	75
Patients/paramedic	2.22	3.06	2.90	1.68	1.16	2.37	1.89	1.60	2.04

† 2011 Census[20] †† Number of suspected stroke patients assessed face-to-face 2015/6

E&W: England & Wales; EEAS – East of England Ambulance Service NHS Trust; EMAS – East Midlands Ambulance Service NHS Trust; LAS – London Ambulance Service; SCAS – South Central Ambulance Service NHS Foundation Trust; SWAS – South Western Ambulance Service NHS Foundation Trust; WAS – Welsh Ambulance Service NHS Trust; YAS – Yorkshire Ambulance Service NHS Trust.

For peer review only - http://bmjopen.bmj.com/site/about/guidelines.xhtml

Comparison of trial and non-trial patients

One ambulance service provided response time interval and distance data for a cohort (n=49) of confirmed stroke patients who were not enrolled into RIGHT-2 (attended by non-trial trained paramedics) but were transported to the same specialist stroke centres participating in the trial.

Statistical Analysis

Time intervals (in minutes), distances (kilometres, km) and baseline characteristics were compared between ambulance services using chi-square and Kruskal-Wallis (one-way ANOVA on ranks) tests. Multiple comparison procedures (Dunn's with Bonferroni correction) were used to assess which ambulance service differed from the others. Spearman and point-biserial correlations were performed to identify the relationship between baseline variables, times and distances. Data are number (%), median [interquartile range] or mean (standard deviation). Statistical significance was defined overall at $p<0.05$, and at $p<0.001$ for correlation matrices and multiple comparisons. Statistical analyses were conducted with SPSS version 24 (IBM, New York).

Patient and Public Involvement

This study was supported by public members of the trial steering committee who were involved throughout, including in trial design, development, conduct, periodic review and dissemination of results.

For peer review only - http://bmjopen.bmj.com/site/about/guidelines.xhtml

RESULTS

RIGHT-2 recruited 1,149 patients between September 2015 and May 2018. Table 1 outlines patient recruitment across the various participating ambulance services, which collectively covered an area of 122,065 km^2 in England and Wales (i.e. 42% of the land area of these countries). Ambulance services varied considerably in size (1,605 km^2 vs 25,899 km^2), population served per service (2.9M vs 8.6M)[20] and annual stroke events (7,400 vs 13,118) (Table 1). Altogether 1,492 paramedics volunteered to be trained in the trial, of whom 516 (36%) recruited at least one patient. Where two or more trial paramedics were present at scene, the paramedic initiating randomisation was credited. On average, 2.2 patients were recruited by each paramedic who enrolled at least one patient although this varied between ambulance services (1.1 vs 3.1).

Patient characteristics

Of the 1,149 patients recruited, average age was 73 (15) years, female 48%, BP 162 (25)/92 (18) mmHg, Glasgow coma scale (GCS) 13.9 (1.7), and FAST score of three 60% (Supplemental Table II). The final diagnosis varied between ambulance services, with the rate of conditions mimicking acute neurovascular disease ranging from 14.3% to 36.1%. This is consistent with other pre-hospital trials without physician presence or mobile stroke unit care, and the rate of stroke mimic reported here is explored elsewhere.[21] Baseline temperature also varied. Otherwise, baseline characteristics did not differ between ambulance service. As age increased, BP and glucose were higher, and heart rate, FAST and GCS lower (Supplemental Table III). 603 (53%) patients gave informed consent, 431 (38%) by relative and 115 (10%) by paramedic witnessed by a colleague on scene.

Time Intervals

The time intervals for various stages in the journey from stroke scene to hospital are shown in Supplemental Table IV. Overall, the median time from symptom onset to emergency call was 19 [interquartile range 5, 64] minutes and this did not differ between ambulance services (Supplemental Tables IV and V). The median time from emergency call to ambulance dispatch was 3 [1, 7] minutes and varied between ambulance service (1 minute vs 5 minutes). An ambulance resource arrived at scene within 8 [5, 13] minutes from being dispatched (and 10 [6, 16] minutes if only including RIGHT-2 trained paramedics) with this varying between ambulance service

For peer review only - http://bmjopen.bmj.com/site/about/guidelines.xhtml

(8 minutes vs 12 minutes).

The median time from onset of symptoms to randomisation was 71 [45, 116] minutes (Table 2, Figure 1) and this varied between ambulance service (53 minutes vs 77 minutes). Significantly, randomisation occurred within 30 and 60 minutes of symptom onset in 104 (9.1%) and 491 (42.9%) participants respectively (Table 2). Ambulance resources spent a median of 33 [26, 46] minutes on scene, though this varied between ambulance services (29 minutes vs 43 minutes) (Supplemental Table IV). Importantly, time on scene did not differ significantly when comparing RIGHT-2 patients versus non-RIGHT-2 patients 34 [26, 44] and 32 [23, 41] minutes respectively (Supplemental Table VI). Transfer time from scene to hospital was a median of 15 [10, 23] minutes, but varied between ambulance service (9 minutes vs 24 minutes) (Supplemental Table IV). The overall time from symptom onset to arrival at hospital was 97 [71, 141] minutes and also varied between ambulance services (86 minutes vs 109 minutes) (Supplemental Table VII). Time at scene was strongly positively correlated with time from scene to hospital (Table 3).

For peer review only

For peer review only - http://bmjopen.bmj.com/site/about/guidelines.xhtml

Table 2. Timings: Symptom onset to randomisation (OTR) (minutes). Data are N (%), median [25, 75 centile]; comparison by Kruskal-Wallis test.

Minutes	E&W	EEAS	EMAS	LAS	SCAS	SWAS	WAS	WMAS	YAS	2p
OTR										
N (%)	1149	178 (15.5)	218 (19.0)	202 (17.6)	7 (0.6)	265 (23.1)	89 (7.7)	37 (3.2)	153 (13.3)	
Median [25, 75 centile]	71 [45, 116]	73 [47, 120]	59 [35, 100]	77 [51, 124]	53 [45, 65]	75 [49, 107]	75 [48, 123]	60 [32, 115]	70 [45, 118]	0.001
N (%)										<0.001
≤30	104 (9.1)	15 (8.4)	38 (17.4)	11 (5.4)	1 (14.3)	16 (6.0)	7 (7.9)	6 (16.2)	10 (6.5)	
31-60	387 (33.8)	63 (35.4)	82 (37.6)	61 (30.2)	3 (42.9)	82 (30.9)	28 (31.5)	13 (35.1)	56 (36.6)	
61-90	258 (22.5)	32 (18.0)	34 (15.6)	51(25.2)	0 (0.0)	77 (29.1)	19 (21.3)	5 (13.5)	38 (24.8)	
91-120	136 (15.1)	25 (14.0)	19 (8.7)	25 (12.4)	0 (0.0)	36 (13.6)	13 (14.6)	5 (13.5)	13 (8.5)	
121-180	173 (15.1)	30 (16.9)	33 (15.1)	28 (13.9)	0 (0.0)	40 (15.1)	16 (18.0)	6 (16.2)	20 (13.1)	
181-240	76 (6.6)	12 (6.7)	9 (4.1)	20 (9.9)	0 (0.0)	13 (4.9)	5 (5.6)	2 (5.4)	15 (9.8)	
>240	15 (1.2)	1 (0.5)	3 (1.4)	6 (3.0)	1 (14.3)	1 (0.4)	1 (1.1)	0 (0.0)	2 (0.7)	

For peer review only - http://bmjopen.bmj.com/site/about/guidelines.xhtml

Table 3. Univariate Correlation between severity of symptoms, timings and distance from scene to hospital. Data are Spearman's coefficient (p-value).

	OTR	FAST	GCS	Scene	OTH	STH	Km
OTC	0.836 (<0.001)	-0.130 (<0.001)	0.086 (0.003)	0.05 (0.088)	0.802 (<0.001)	0.007 (0.80)	-0.070 (0.033)
OTR		-0.135 (<0.001)	0.102 (0.001)	0.263 (<0.001)	0.941 (<0.001)	0.244 (<0.001)	-0.61 (0.61)
FAST			-0.157 (<0.001)	-0.066 (0.026)	-0.133 (<0.001)	-0.046 (0.12)	0.803 (0.93)
GCS				-0.008 (0.80)	0.115 (<0.001)	0.084 (0.004)	0.076 (0.017)
Scene					0.326 (<0.001)	0.791 (<0.001)	0.104 (0.002)
OTH						0.403 (<0.001)	0.216 (<0.001)
STH							0.554 (<0.001)

OTC: onset to emergency call; OTR: onset to randomisation; FAST: face, arm, speech, time score. Scene: Total time spent at scene; STH: Time from scene to reach hospital; Km: distance (kilometres) from scene to hospital.

For peer review only

For peer review only - http://bmjopen.bmj.com/site/about/guidelines.xhtml

Distances

The median distance travelled from the postcode of the suspected stroke scene to the receiving hospital was 10.0 [4.4, 18.4] km, with considerable variation between ambulance services (4.1 km vs 19.9 km) (Supplemental Table VIII). Time from scene to hospital was moderately positively correlated with distance from scene to hospital. (Supplemental Figures I:A-G present geographical distribution of randomisation by ambulance service)

Urban versus rural services

When comparing urban and rural ambulance services (Supplemental Table I), there was no difference in receipt of the emergency call to dispatching a resource to scene, nor a difference in onset of symptoms to randomisation. The time spent at scene was marginally longer in rural locations and, as anticipated, both conveyance time and distance to the stroke centre was statistically different.

Comparison of trial and non-trial patients

In the ambulance service with times available for patients not enrolled in the trial, on scene to hospital arrival differed among patients enrolled and not enrolled in RIGHT-2, 10 [0.4, 64.7] versus 16 [7.6, 24.0] minutes (Supplemental Table VIII). The median distance from dispatch location to scene in the ambulance service with this available (EMAS) was 7.3 km [3.5, 12.0].

For peer review only - http://bmjopen.bmj.com/site/about/guidelines.xhtml

DISCUSSION

In this large national prehospital trial, 516 paramedics from eight ambulance services across England and Wales successfully recruited 1,149 participants and transported them to 54 hospitals. Paramedics assessed and diagnosed suspected stroke, consented patients and initiated randomised treatment. Key timings were: onset to emergency call 19 minutes, onset to scene 40 minutes, onset to randomisation 71 minutes, time at scene 33 minutes, randomisation to hospital 24 minutes, and depart scene to hospital arrival 15 minutes; all but the first two differed between ambulance services. The average distance travelled by one ambulance service from dispatch location to scene was 7.3 km and 10.0 km from scene to hospital for all participating ambulance services.

Pre-hospital time intervals in acute stroke have been described previously,[12 22-26] but rarely in randomised trials.[4 11 13] The symptom onset to randomisation time of 71 minutes in RIGHT-2 is consistent with two previous UK ambulance-based stroke trials (RIGHT 55 minutes; Paramedic Initiated Lisinopril For Acute Stroke Treatment (PIL-FAST) 70 minutes)[27 28] although these were small single centre pilot studies undertaken largely in urban settings. The large US Field Administration of Stroke Therapy - Magnesium trial[29] (FAST-MAG) reported a median of 45 minutes from symptom onset to receipt of study drug. Nevertheless, these times are all longer than UK multi-centre ambulance-based trials outside of stroke, notably the AIRWAYS-2 and PARAMEDIC-2 trials in cardiac arrest.[30 31] In PARAMEDIC-2, the onset of symptoms to initiation of treatment in the intervention group was just 21.5 minutes. The most important driver of this difference is most likely shorter onset to call times for cardiac arrest patients than for stroke, and suspected stroke may require more complex assessment both by call handlers and by paramedics on scene. Additional contributors are that cardiac arrest is allocated the highest dispatch priority, an immediate response and patients receive immediate trial treatment with emergency waiver of consent.

The explanation for differences in timings is probably multifactorial but the degree of urban versus rural population is one likely explanation. This was apparent for time spent at scene and both time and distance to hospital. As expected, there were no differences for receipt of call to dispatch, arrival of RIGHT-2 trained paramedic at scene nor onset of symptoms to randomisation.

For peer review only - http://bmjopen.bmj.com/site/about/guidelines.xhtml

There are several strengths of this study. First, RIGHT-2 involved eight of 11 ambulance services in England and Wales. Of those not participating, two were unable to join because they were involved in another ambulance-based stroke trial[32] and the other involved hospitals that were concerned about adversely impacting on recruitment to commercial trials. 516 paramedics (of 1,492 trained in RIGHT-2 procedures) consented and randomised a large number of participants, adhered to the protocol and completed specific data recording. It is noted that there are marked differences in recruit numbers between ambulance services. This, in part, is accounted for due to low recruitment during the initial recruitment phase requiring broadening of ambulance services from five to eight and stroke centres from 30-54. Furthermore, recruitment hours initially limited to typical working hours for research staff availability were extended to encompass 24/7 recruitment reflective of real-world ambulance care to not limit participation and maximise inclusion. Conflictingly, a small number of stroke centres closed recruitment to ambulances once target numbers of participants had been received and before the end of the recruitment phase highlighting the challenging reliance on dual centres when dealing with research in pre-hospital stroke.

Second, the consent model applied in RIGHT-2 is unlike any other large-scale ambulance-based studies worldwide to date and builds upon previous UK based pre-hospital stroke pilots.[27 33] Other pre-hospital trials in stroke have relied upon models of either informed consent,[34] deferred consent[35] or consent by doctor (present or remote).[29 36] Stroke is complex due to the varying nature of severity of presentations where patients' ability to consent in an informed manner to participate in a clinical study should not be overlooked preserving patient autonomy in accordance with the declaration of Helsinki.[37 38] Notwithstanding the complexities of emergency presentations that could impact upon decision-making, mental capacity or short intervention windows and the impact these situations bring to truly informed patient consent, the combined consent approach in RIGHT-2 acknowledges patient autonomy without precluding participation from those who are unable to voice their opinion or who lack presence of a proxy to consent on their behalf.[38] Mechanisms to safeguard consent were built into the protocol through reconfirmation of consent once in hospital for the both the pre-hospital and in-hospital elements respectively, and patient and public representatives were fully embedded within protocol development and steering group oversight of the trial.[14]

For peer review only - http://bmjopen.bmj.com/site/about/guidelines.xhtml

Third, the protocol required flexibility and adaptation to align with individual operational processes specific to each ambulance service to ensure successful delivery of the trial. Fourth, detailed logistic information on timing and distances travelled were collected. Last, the results highlight the successful delivery of a simple, ambulance-based intervention with 43% of patients receiving the intervention within two-hours of symptom onset without compromising time on scene required to complete additional research activity.

There are also several study limitations. Firstly, it is recognised that not every receiving stroke unit within each ambulance service region could participate in RIGHT-2 due to capacity and competing research,[32 39] (this included concurrent commercial and post-arrival trials). Therefore, it must be considered that the timing and logistic data of participating hospitals may not be fully representative of all urban and rural locations. However, the intention was not to assess the differences between urban and rural settings, but to shed light on the conduct and deliverability of a pre-hospital intervention in stroke where time and distance may impede access to specialist stroke services. Furthermore, stroke unit hours of operation varied across the 54 centres with a small number of sites not accepting patients outside working hours which impacted paramedics' decisions to randomise. This reduces the reflection of real-world emergency stroke care. The duration of recruitment varied between regions due to complexities in setting up multi-centre research

Additionally, it is acknowledged that the recruitment criteria were broad which resulted in a higher than anticipated proportion of stroke mimics. To mitigate this, mobile stroke unit care is an emerging field where imaging and definitive care delivery at the scene reduces time delays in stroke[40] and could offer improved confidence and precision of diagnosis for prehospital trial enrolment.

Recognising that 516 of 1,492 RIGHT-2 trained paramedics (36%) identified and randomised eligible patients, this is consistent with other trials in pre-hospital stroke.[27] This, in part, is due to the voluntary participation of paramedics in research where records suggest that only one third of the paramedic workforce participate.[41] Further, in a UK system where response time is one benchmark of the quality of ambulance service provision, ambulance dispatchers are not able to assign specific research-trained personnel to specific emergency calls, instead allocating the nearest

For peer review only - http://bmjopen.bmj.com/site/about/guidelines.xhtml

available resource to attend. Low recruitment must be considered during the development of ambulance-based trials and this factor alone has previously resulted in extended recruitment phases, retraining of researchers and extensive study drug availability to achieve pre-planned sample sizes.[29 35 42]

Finally, this paper does not capture the time-interval between arrival at hospital and handover to the hospital team, nor the time of the ambulance becoming available for the next emergency call (hospital turnaround). During the hospital turnaround period, ambulance staff hand-over the patient to hospital staff, complete relevant documentation and prepare the vehicle for the next assignment. A rapid hospital turnaround is important for making the vehicle available for waiting emergency calls. Whilst the addition of research activity at scene may not delay enrolled patient treatment, it is possible that delay required to complete additional research activity steps after patient handover may prolong the turnaround phase.

In summary, we completed a large pre-hospital stroke trial involving a simple-to-administer intervention across multiple ambulance services. The time from onset to randomisation and modest distances travelled support the applicability of future large-scale paramedic-delivered ambulance-based stroke trials in urban and rural locations. Nevertheless, pre-hospital time intervals and distances from scene-to-hospital varied by ambulance service and this was, at least in part, explained by the type of urban versus rural population. Although our results may not be generalisable to all ambulance service settings, they do inform future developments in ambulance-based stroke care and provide support to the deliverability of future large-scale multicentre pre-hospital paramedic-delivered ambulance-based acute stroke trials.

For peer review only

For peer review only - http://bmjopen.bmj.com/site/about/guidelines.xhtml

ACKNOWLEDGEMENTS

We thank the patients who participated in this trial and their relatives, the clinical and research teams of the various ambulance services and hospitals, and the paramedics who participated, recruited and treated the patients.

The following ambulance service colleagues are acknowledged who contributed to trial implementation, data collection and revision of the draft paper: Roderick Johnson, paramedic clinical operations manager (EMAS), Debbie Shaw, clinical audit and research manager (EMAS), Robert Spaight, head of research and audit (EMAS), Larissa Prothero, research paramedic EEAS), Theresa Foster, research manager (EEAS), Rachael T Fothergill, Research Manager (LAS), Heather Cole, Research paramedic (LAS), Joanna Lazarus, Research paramedic (LAS), Helen Werts, research paramedic (LAS), Neil Thomson, Medical Director (LAS), Helen Pocock, senior research paramedic (SCAS), Kurtis Poole, research paramedic (SCAS), Ed England, research manager (SCAS), Maria Robinson, research manager (SWAS), Katherine Zorab, Quality Improvement Paramedic (SWAS), Adrian South, Clinical Director (SWAS), Catrin Convery, research paramedic (WAS), Tim Pearce, Paramedic Team Leader (WAS), Nigel Rees, Head of Research and Development (WAS), Joshua Miller, research paramedic (WMAS), Imogen Gunson, research paramedic (WMAS), Andrew Rosser, head of research (WMAS), Matthew Ward, consultant paramedic (WMAS), Kelly Hird, research paramedic (YAS), Jacqui Crossley, assistant clinical director (YAS).

We acknowledge support of the English National Institute for Health Research (NIHR) Clinical Research Network, Health and Care Research Wales, and that the coordination between multiple ambulance services and hospitals, and large recruitment would not have been possible without NIHR network support. A complete list of the Rapid Intervention with Glyceryl trinitrate in Hypertensive stroke Trial-2 (RIGHT-2) Investigators is provided in the primary publication.[17]

We acknowledge Eivind Berge who contributed to this paper but sadly passed away prior to publication.

For peer review only

For peer review only - http://bmjopen.bmj.com/site/about/guidelines.xhtml

SOURCES OF FUNDING

This work was supported by the British Heart Foundation [grant number CS/14/4/30972].

ETHICAL APPROVAL

RIGHT-2 was approved by the UK regulator (Medicines and Healthcare products Regulatory Agency, reference: 03057/0064/001–0001; Eudract 2015–000115–40) and national research ethics committee (IRAS: 167115) and was adopted by the National Institute for Health Research Clinical Research Network.

TRANSPARENCY

The lead and senior authors affirms that this manuscript is an honest, accurate, and transparent account of the study being reported; that no important aspects of the study have been omitted; and that any discrepancies from the study as planned have been explained.

DATA AVAILABILITY STATEMENT

Individual participant data are shared with the Blood pressure in Acute Stroke Collaboration (BASC) and Virtual International Stroke Trials Archive (VISTA). No further data available.

CONTRIBUTORSHIP STATEMENT

PMB, also principal investigator, and MD conceived the study. All authors contributed to the planning, design and conduct. LJH was responsible for data curation. PS and LJW supported with statistical analysis. All authors contributed to the reporting, analysis and interpretation of the results. MD and PMB led the writing of the manuscript with critical revision from JPA, PS, LJW, LJH, DH, JW and ANS.

COMPETING INTERESTS

JPA was funded in part by the BHF during the conduct of the study and is supported by the National Institute for Health Research (NIHR) West Midlands Health and Care Research Scholars Programme. CSA reports grants from National Health and Medical Research Council (NHMRC) of Australia, grants from Takeda, and personal fees from Takeda, Amgen, and Boehringer Ingelheim outside of the submitted work. MD was

For peer review only

For peer review only - http://bmjopen.bmj.com/site/about/guidelines.xhtml

funded by the BHF during the conduct of the study. TJE, and AAM report grants from BHF during the conduct of the study. GM is supported by NHS Lothian Research and Development Office and reports grants from The Stroke Association (TSA), The Royal College of Radiologists. CIP reports grants from Nottingham University and BHF during the conduct of the study. TGR is an NIHR Senior Investigator, and reports grants from BHF during the conduct of the study. CR reports grants from NIHR Health Technology Assessment during the conduct of the study; personal fees from Allergan, Air Liquide, Merz, Boehringer, Bayer, Johnson & Johnson, Sanofi and Emtensor; non-financial support from European Stroke Conference and Trident; and other support from Firstkind Medical, Medtronic and Brainomix outside the submitted work. PMR reports grants from Wellcome Trust during the conduct of the study. ECS reports personal fees from Novartis and Bayer outside of the submitted work. JMW reports grants from the BHF during the conduct of the study; and grants from Medical Research Council, Chief Scientist Office, Leducq, EU H2020, TSA, BHF and Alzheimer's Society outside the submitted work. NSp reports grants from BHF and RCUK, during the conduct of the study. PMB is Stroke Association Professor of Stroke Medicine and is a National Institute Health Research (NIHR) Senior Investigator. He reports grants from British Heart Foundation (BHF) during the conduct of the study; personal fees and other fees from Sanofi, Nestlé, DiaMedica, Moleac, Platelet Solutions, Phagenesis, and ReNeuron, outside the submitted work. All other authors declare no competing interests.

COPYRIGHT

The Corresponding Author has the right to grant on behalf of all authors and does grant on behalf of all authors, an exclusive licence (or nonexclusive for government employees) on a worldwide basis to the BMJ Publishing Group Ltd to permit this article (if accepted) to be published in BMJ editions and any other BMJPGL products and sublicenses such use and exploit all subsidiary rights, as set out in our licence.

For peer review only - http://bmjopen.bmj.com/site/about/guidelines.xhtml

References

1. Joint Royal Colleges Ambulance Liaison Committee, Association of Ambulance Chief Executives. *JRCALC Clinical Guidelines*. Bridgwater: Class Professional Publishing, 2019.
2. Royal College of Physicians. Intercollegiate Stroke Working Party. National clinical guidelines for stroke.**5th Edition 2016**.
3. Meretoja A, Strbian D, Mustanoja S, et al. Reducing in-hospital delay to 20 minutes in stroke thrombolysis. *Neurology* 2012;**79**(4):306-13.
4. Koch P, Kunz A, Ebinger M, et al. Influence of Distance to Scene on Time to Thrombolysis in a Specialized Stroke Ambulance. *Stroke* 2016;**47**(8):2136-40.
5. Emberson J, Lees K, Lyden P, et al. Effect of treatment delay, age and stroke severity on the effects of intravenous thrombolysis with alteplase for acute ischaemic stroke: a meta-analysis of individual patient data from randomised trials. *Lancet* 2014;**384**(9958):1929-35.
6. Bourcier R, Goyal M, Liebeskind DS, et al. Association of Time From Stroke Onset to Groin Puncture With Quality of Reperfusion After Mechanical Thrombectomy: A Meta-analysis of Individual Patient Data From 7 Randomized Clinical Trials. *JAMA Neurol* 2019;**76**(4):405-11.
7. Wolters FJ, Paul NL, Li L, et al. Sustained impact of UK FAST-test public education on response to stroke: a population-based time-series study. *Int J Stroke* 2015;**10**(7):1108-14.
8. Ong ME, Cho J, Ma MH, et al. Comparison of emergency medical services systems in the pan-Asian resuscitation outcomes study countries: Report from a literature review and survey. *Emerg Med Australas* 2013;**25**(1):55-63.
9. Simonsen SA, Andresen M, Michelsen L, et al. Evaluation of pre-hospital transport time of stroke patients to thrombolytic treatment. *Scandinavian Journal of Trauma, Resuscitation & Emergency Medicine* 2014;**22**:65.
10. Oostema JA, Konen J, Chassee T, et al. Clinical predictors of accurate prehospital stroke recognition. *Stroke* 2015;**46**(6):1513-7.
11. Ramanujam P, Castillo E, Patel E, et al. Prehospital transport time intervals for acute stroke patients. *J Emerg Med* 2009;**37**(1):40-5.
12. Simonsen SA, Andresen M, Michelsen L, et al. Evaluation of pre-hospital transport time of stroke patients to thrombolytic treatment. *Scand J Trauma Resusc Emerg Med* 2014;**22**:65.
13. Price CI, Shaw L, Islam S, et al. Effect of an Enhanced Paramedic Acute Stroke Treatment Assessment on Thrombolysis Delivery During Emergency Stroke Care: A Cluster Randomized Clinical Trial. *JAMA Neurol* 2020;**77**(7):840-48.
14. Appleton JP, Scutt P, Dixon M, et al. Ambulance-delivered transdermal glyceryl trinitrate versus sham for ultra-acute stroke: Rationale, design and protocol for the Rapid Intervention with Glyceryl trinitrate in Hypertensive stroke Trial-2 (RIGHT-2) trial (ISRCTN26986053). *Int J Stroke* 2017;**0**(0):1-16.
15. Scutt P, Appleton JP, Dixon M, et al. Statistical analysis plan for the 'Rapid Intervention with Glyceryl trinitrate in Hypertensive stroke Trial-2 (RIGHT-2)'. *Eur J Stroke* 2018;**3**(2):193-96.
16. Bath PM, Scutt P, Appleton JP, et al. Baseline characteristics of the 1149 patients recruited into the Rapid Intervention with Glyceryl trinitrate in Hypertensive stroke Trial-2 (RIGHT-2) randomized controlled trial. *Int J Stroke* 2019;**14**(3):298-305.
17. RIGHT-2 Investigators. Prehospital transdermal glyceryl trinitrate in patients with ultra-acute presumed stroke (RIGHT-2): an ambulance-based, randomised, sham-controlled, blinded, phase 3 trial. *Lancet* 2019;**393**(10175):1009-20.

For peer review only - http://bmjopen.bmj.com/site/about/guidelines.xhtml

18. Bath PM, Woodhouse LJ, Krishnan K, et al. Pre-hospital transdermal glyceryl trinitrate for ultra-acute intracerebral haemorrhage- data from the RIGHT-2 trial. *Stroke* 2019;**In press**.
19. Appleton JP, Scutt P, Dixon M, et al. Ambulance-delivered transdermal glyceryl trinitrate versus sham for ultra-acute stroke: Rationale, design and protocol for the Rapid Intervention with Glyceryl trinitrate in Hypertensive stroke Trial-2 (RIGHT-2) trial (ISRCTN26986053). *Int J Stroke* 2017:1747493017724627.
20. Office for National Statistics. 2011 Census Analysis - Comparing Rural and Urban Areas of England and Wales. In: Statistics OfN, ed. London: Office for National Statistics, 2013.
21. Tunnage B, Woodhouse LJ, Dixon M, et al. Pre-hospital transdermal glyceryl trinitrate in patients with stroke mimics: data from the RIGHT-2 randomised-controlled ambulance trial. *BMC Emerg Med* 2022;**22**(1):2.
22. Citerio G, Galli D, Pesenti A. Early stroke care in Italy - a steep way ahead: an observational study. *Emerg Med J* 2006;**23**:608-11.
23. Puolakka T, Vayrynen T, Happola O, et al. Sequential analysis of pretreatment delays in stroke thrombolysis. *Acad Emerg Med* 2010;**17**(9):965-9.
24. Mosley I, Nicol M, Donnan G, et al. Stroke symptoms and the decision to call for an ambulance. *Stroke* 2007;**38**(2):361-6.
25. Patel MD, Brice JH, Moss C, et al. An evaluation of emergency medical services stroke protocols and scene times. *Prehosp Emerg Care* 2014;**18**(1):15-21.
26. Drenck N, Viereck S, Bækgaard JS, et al. Pre-hospital management of acute stroke patients eligible for thrombolysis - an evaluation of ambulance on-scene time. *Scand J Trauma Resusc Emerg Med* 2019;**27**(1):10.1186/s13049-018-0580-4.
27. Shaw L, Price C, McLure S, et al. Paramedic Initiated Lisinopril For Acute Stroke Treatment (PIL-FAST): results from the pilot randomised controlled trial. *Emerg Med J* 2014;**31**(12):994-9.
28. Ankolekar S, Fuller M, Cross I, et al. Feasibility of an Ambulance-Based Stroke Trial, and Safety of Glyceryl Trinitrate in Ultra-Acute Stroke The Rapid Intervention With Glyceryl Trinitrate in Hypertensive Stroke Trial (RIGHT, ISRCTN66434824). *Stroke* 2013;**44**(11):3120-28.
29. Saver J, Starkman S, Eckstein M, et al. Prehospital use of magnesium sulfate as neuroprotection in acute stroke. *N Engl J Med* 2015;**372**(6):528-36.
30. Perkins GD, Ji C, Deakin CD, et al. A Randomized Trial of Epinephrine in Out-of-Hospital Cardiac Arrest. *N Engl J Med* 2018;**379**(8):711-21.
31. Benger JR, Kirby K, Black S, et al. Effect of a Strategy of a Supraglottic Airway Device vs Tracheal Intubation During Out-of-Hospital Cardiac Arrest on Functional Outcome: The AIRWAYS-2 Randomized Clinical Trial. *JAMA* 2018;**320**(8):779-91.
32. Price CI, Shaw L, Dodd P, et al. Paramedic Acute Stroke Treatment Assessment (PASTA): study protocol for a randomised controlled trial. *Trials* 2019;**20**(1):121.
33. Ankolekar S, Fuller M, Sprigg N, et al. Rapid Intervention with Glyceryl trinitrate (GTN) in Hypertensive stroke Trial (RIGHT): safety of GTN and potential of ambulance trials in ultra-acute stroke. *International Journal of Stroke* 2012;**7**:7-7.
34. Nurmi J, Lindsberg PJ, Happola O, et al. Strict glucose control after acute stroke can be provided in the prehospital setting. *Acad Emerg Med* 2011;**18**(4):436-9.
35. Hougaard K, Hjort N, Zeidler D, et al. Remote ischemic perconditioning as an adjunct therapy to thrombolysis in patients with acute ischemic stroke: a randomized trial. *Stroke* 2013;**45**:159-67.

For peer review only - http://bmjopen.bmj.com/site/about/guidelines.xhtml

36. Larsson M, Castrén M, Lindström V, et al. Prehospital exenatide in hyperglycemic stroke-A randomized trial. *Acta Neurol Scand* 2019;**140**(6):443-48.
37. Rickham PP. Human experimentation. Code of ethics of the World Medical Association. Declaration of Helsinki. *Br Med J* 1964;**2**(5402):177.
38. Goyal M, Ospel JM, Ganesh A, et al. Rethinking Consent for Stroke Trials in Time-Sensitive Situations: Insights From the COVID-19 Pandemic. *Stroke* 2021;**52**(4):1527-31.
39. Muñoz-Venturelli P, Arima H, Lavados P, et al. Head Position in Stroke Trial (HeadPoST)--sitting-up vs lying-flat positioning of patients with acute stroke: study protocol for a cluster randomised controlled trial. *Trials* 2015;**16**:256.
40. Fassbender K, Merzou F, Lesmeister M, et al. Impact of mobile stroke units. *J Neurol Neurosurg Psychiatry* 2021.
41. Pocock H, Deakin CD, Quinn T, et al. Human factors in prehospital research: lessons from the PARAMEDIC trial. *Emergency Medicine Journal* 2016;**33**(8):562-8.
42. De Luca A, Toni D, Lauria L, et al. An emergency clinical pathway for stroke patients - results of a cluster randomised trial (isrctn41456865). *Bmc Health Services Research* 2009;**9**:10.

For peer review only

For peer review only - http://bmjopen.bmj.com/site/about/guidelines.xhtml

Figure 1. Box Plot of Onset to Randomisation

See separate file

For peer review only

For peer review only - http://bmjopen.bmj.com/site/about/guidelines.xhtml

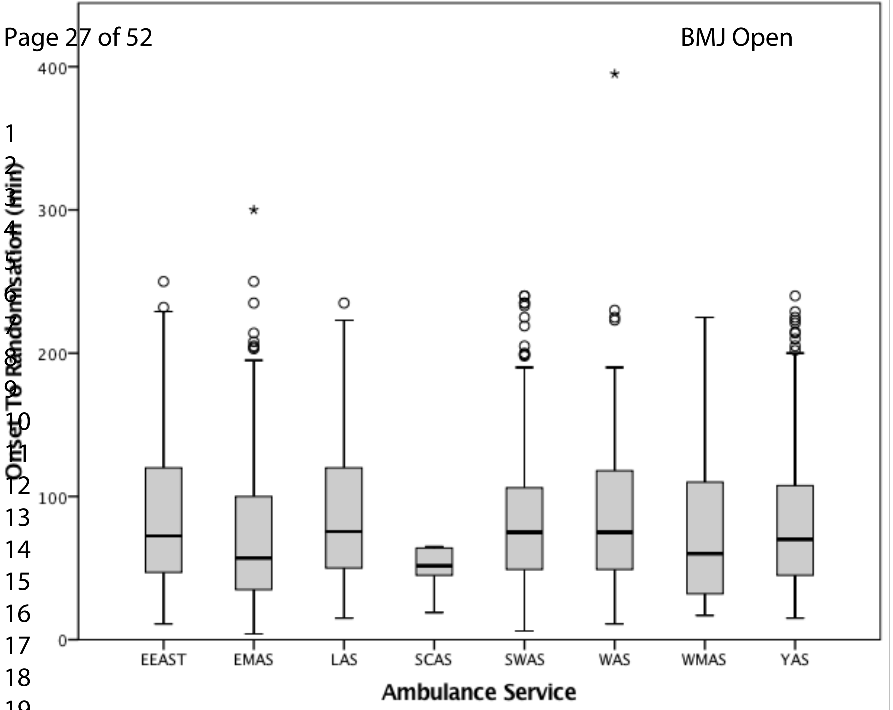
Onset To Randomisation (min)
400
300
200
100
0
EEAST
EMAS
LAS
SCAS
SWAS
WAS
WMAS
YAS
Ambulance Service

SUPPLEMENTAL MATERIAL

Time intervals and distances travelled for pre-hospital ambulance stroke care: data from the Rapid Intervention with Glyceryl trinitrate in Hypertensive stroke Trial-2 (RIGHT-2)

TABLE OF CONTENTS

	Page
Title	1
Supplemental Background	1
Supplemental Methods	1
Supplemental Results	2
List of supplemental Tables and Figures	5
Supplemental Tables (start from)	6
Supplemental Figures (start from)	14
Supplemental References	22

SUPPLEMENTAL BACKGROUND

In the 2011 census of England and Wales, 43.7 million people lived in urban areas (defined as an area with greater than 10,000 inhabitants[1]) with 9.3 million people (17.6%) living in rural areas. Conversely, rural areas cover 85% of the land area of England and Wales. The proportion of urban and rural populations covered varied between ambulance service with rural ranging from 0.2% (LAS) to 32.8% (WAS).

SUPPLEMENTAL METHODS

RIGHT-2 trial

Patients were excluded if they had any of the following: resided in a nursing home, hypoglycaemia (<2.5mmol/l), evidence of seizure at presentation, known to have a terminal illness, taken sildenafil (or equivalent) within 24 hours, or previously been enrolled into RIGHT-2.

For peer review only

Patients were randomised (1:1) to receive either GTN patches or sham patches. A randomisation sequence was generated by the trial programmer at the Nottingham Stroke Trials Unit using random-permuted fixed-size blocks stratified by ambulance station. Identical looking numbered treatment packs were sent in blocks (four treatment packs per block) to each ambulance station. Trial-trained paramedics only carry one treatment pack at any time.

Participants were taken to hospitals with an acute stroke service - these joined the trial depending on capacity and feasibility to receive enrolled patients and deliver the trial protocol.[2] Information on participants was entered into the main trial database by hospital-based research staff, including the data collected in the ambulance (baseline, during treatment) and in hospital (admission, day 2, discharge or death, final diagnosis).

The trial was funded by the British Heart Foundation, sponsored by the University of Nottingham, had Health Research Authority ethics committee approval, and was eligible for National Institute of Health Research Clinical Research Network support.

Data sharing

Individual participant data will be shared with the Blood pressure in Acute Stroke Collaboration (BASC) and Virtual International Stroke Trials Archive (VISTA). From 1 Jan 2022, the Chief Investigator (with approval from the Trial Steering Committee as necessary) will consider other requests to share individual participant data via email at: right-2@nottingham.ac.uk. We will require a protocol detailing hypothesis, aims, analyses, and intended tables and figures. Where possible, we will perform the analyses; alternatively, de-identified data and a data dictionary will be supplied for the necessary variables for remote analysis. Any sharing will be subject to a signed data access agreement. Ultimately, the entire trial dataset will be published.

SUPPLEMENTAL RESULTS

Although all 10 stroke-receiving hospitals participated in the trial in the EMAS area, not all hospitals in the other seven ambulance services took part in the trial (range 1-13; Table 1).

For peer review only - http://bmjopen.bmj.com/site/about/guidelines.xhtml

Time Intervals

The extended time from the emergency call to dispatch of 5 minutes for SWAS was due, in part, to a service-wide evaluation of a new ambulance dispatch model (Ambulance Response Programme). The extended time from dispatch to arrival at scene of 12 minutes for WAS is likely to reflect, in part, the high proportion of rural-based patients in Wales and the WAS Clinical Response Model which was introduced during the trial and differs to England.

The time on scene in EMAS was comparable to a cohort of non-RIGHT-2 stroke patients despite the addition of consent, randomisation and treatment activities (34 [26, 44] vs 32 [23, 41] minutes, p=0.12) (Supplemental Table VI).

RIGHT-2 treatment was administered to 910 (80%) patients prior to departing scene and 239 (20%) patients en-route to hospital highlighting the speed and simplicity of the intervention.

Multiple comparison testing for Supplemental Table IV:

Significant differences existed between ambulance service with regards to timings:

- Emergemcy call to dispatch: LAS differs from EEAS p<0.001, EMAS p<0.001, SCAS p=0.011, SWAS p=0.008, WAS p<0.001 WMAS p=0.015 and YAS p=0.002. SWAS differs from EEAS p<0.001, EMAS p<0.001, SCAS p=0.001, WAS p<0.001 and YAS p<0.001
- Dispatch to arrival at scene: LAS differs from EEAS p=0.022, YAS p<0.001, SWAS p<0.001 and WAS p<0.001. EMAS differs from YAS p=0.024, SWAS p<0.001 and WAS p=0.003. EEAS differs from WAS p=0.036
- Dispatch to arrival of RIGHT-2 Paramedic: LAS differs from SWAS p=0.012, EEAS P=0.019, YAS p=0.002 and WAS p=0.003. EMAS differs from YAS p=0.037 and WAS p=0.030
- Time of arrival on scene to randomisation: WMAS differs from EEAS p=0.011, WAS p=0.015 and LAS p=0.001. EMAS differs from SWAS p<0.001, EEAS p<0.001, WAS p<0.001 and LAS p<0.001. YAS differs from EEAS p=0.003, WAS p=0.013 and LAS p<0.001
- Onset to randomisation: EMAS differs from SWAS p=0.021 and LAS p<0.001

For peer review only - http://bmjopen.bmj.com/site/about/guidelines.xhtml

- Consent to randomisation: LAS differs from WMAS p=0.030, SWAS p<0.001, EMAS p<0.001, YAS p<0.001, WAS p<0.001 and EEAS p<0.001. SWAS differs from EEAS p=0.003
- Time on scene: EEAS differs from LAS p<0.001, YAS p=0.006, EMAS p=0.046. LAS differs from WAS p=0.001
- Depart scene to arrival at hospital: WMAS differs from EMAS p=0.018, EEAS p<0.001, WAS p<0.001 and SWAS p<0.001. YAS differs from EEAS p=0.003, WAS p=0.002 and SWAS p<0.001. SWAS differs from LAS p<0.001 and EMAS p<0.001
- Onset to hospital: EMAS differs from SWAS p=0.014 and WAS p=0.043. LAS differs from EEAS p<0.001, EMAS p<0.001, SWAS p<0.001, WAS p<0.001, WMAS p<0.001 and YAS p<0.001
- Randomisation to treatment: LAS differs from EEAS p<0.001, EMAS p<0.001, SWAS p<0.001, SCAS p<0.001, WAS p<0.001, WMAS p<0.001 and YAS p<0.001
- Randomisation to hospital: SWAS differs from YAS p<0.001 and WMAS p=0.023

Distances

Although it was not possible to collect information on where the ambulance was when dispatched to the scene for seven ambulance services, this information was available for EMAS; the median linear distance from point of dispatch to the stroke scene was 7.3 [3.5, 12.0] km.

For peer review only

LIST OF SUPPLEMENTAL TABLES AND FIGURES

Supplemental Tables	
I	Comparison of timings and conveyance distance by urban versus rural geography
II	Demographic and baseline characteristics of patients enrolled into RIGHT-2
III	Univariate correlations between baseline characteristics for age, sex, heart rate, systolic blood pressure and time from onset of symptoms to randomisation
IV	Timings (in minutes) by ambulance service
V	Timings: Onset of symptoms to emergency call
VI	Comparison of EMAS stroke patients in RIGHT-2 versus non-RIGHT-2
VII	Timings: Symptom onset to arrival at hospital
VIII	Conveyance distances

Supplemental Figures	
I	Distribution of Randomisation by Ambulance Service
A	EMAS
B	EEAS
C	SCAS
D	SWAS
E	WAS
F	WMAS
G	YAS

For peer review only

SUPPLEMENTAL TABLE I. Comparison of timings and conveyance distance by urban versus rural geography. Data are median [25, 75 centile]; comparison by Mann-Whitney U test with difference (95% confidence intervals)

	Urban	Rural	Difference (95% CI)	2p
% rural	13.3	30.0	-	-
Timing (minutes)				
Onset to emergency call	23 [8, 70]	17 [5, 58]	19 (17, 23)	0.003
emergency call to dispatch	3 [1, 7]	3 [1, 8]	3 (3, 3)	0.17
Onset to R2 scene	47 [24, 95]	43 [22, 84]	45 (42, 48)	0.06
Onset to randomisation	73 [47, 121]	70 [45, 114]	71 (68, 75)	0.10
Arrive to depart scene	29 [22, 35]	32 [24, 41]	31 (30, 31)	<0.001
Depart scene to hospital	12 [9, 18]	17 [10, 25]	15 (14, 16)	<0.001
Distance (km)				
Conveyance	5.7 [3.3, 10.6]	11.7 [5.0, 20.4]	9.5 (8.6, 10.3)	<0.001

Urban: LAS, WMAS, YAS, SCAS; rural: EMAS, EEAS, SWAS, WAS

For peer review only

For peer review only - http://bmjopen.bmj.com/site/about/guidelines.xhtml

SUPPLEMENTAL TABLE II. Demographic and baseline characteristics of patients enrolled into RIGHT-2

Data are number (%), median [interquartile range] or mean (standard deviation). Comparisons between ambulance services using Chi-square test, Kruskal-Wallis or one-way ANOVA.

Characteristic	E&W	EEAS	EMAS	LAS	SCAS	SWAS	WAS	WMAS	YAS	2p
Patients (%)	1149	178 (15.5)	218 (19.0)	202 (17.6)	7 (0.6)	265 (23.1)	89 (7.7)	37 (3.2)	153 (13.3)	-
Consent from (%)										0.58
Patient	603 (52.5)	96 (52.2)	111 (50.9)	117 (57.9)	4 (57.1)	131 (49.4)	48 (53.9)	18 (48.6)	81 (52.9)	
Relative/Friend	431 (37.5)	73 (41.0)	90 (41.3)	70 (34.7)	1 (14.3)	102 (38.5)	23 (25.8)	15 (40.5)	57 (37.3)	
Paramedic	115 (10.0)	12 (6.7)	17 (7.8)	15 (7.4)	2 (28.6)	32 (12.1)	18 (20.2)	4 (10.8)	15 (9.8)	
Age (years)	72.5 (14.6)	73.4 (13.4)	71.6 (13.9)	70.4 (16.3)	76.6 (15.4)	74.4 (14.7)	74.7 (14.8)	70.2 (17.2)	71.1 (13.7)	0.34
<80 (%)	714 (62.1)	114 (64.0)	144 (66.1)	131 (64.9)	3 (42.9)	150 (56.6)	48 (53.9)	22 (59.5)	102 (66.7)	
>=80 (%)	435 (37.9)	64 (36.0)	74 (33.9)	71 (35.1)	4 (57.1)	115 (43.4)	41 (46.1)	15 (40.2)	51 (33.3)	
Female (%)	555 (48.3)	83 (46.6)	98 (45.0)	106 (52.5)	1 (14.3)	134 (50.6)	44 (49.4)	16 (43.2)	73 (47.7)	
FAST (/3)	2.6 (0.51)	2.5 (0.53)	2.6 (0.50)	2.5 (0.50)	2.1 (0.38)	2.6 (0.51)	2.6 (0.21)	2.6 (0.55)	2.7 (0.50)	0.31
3 N(%)	692 (60.1)	98 (55.1)	137 (62.8)	107 (53.0)	1 (14.3)	166 (62.9)	56 (63.6)	22 (59.5)	103 (67.3)	
2 N(%)	446 (8.8)	77 (43.3)	80 (36.7)	95 (47.0)	9 (85.7)	95 (36.0)	31 (35.2)	14 (37.8)	48 (31.14)	
1 N(%) †	11 (1.0)	3 (1.7)	1 (0.5)	0 (0.0)	0 (0.0)	3 (1.1)	1 (1.1)	1 (2.7)	2 (1.3)	
GCS (/15)	13.9 (1.7)	13.6 (1.8)	14.0 (1.7)	13.9 (1.7)	13.7 (1.8)	14.0 (1.6)	13.9 (1.7)	13.9 (1.7)	13.9 (1.7)	0.50
<14	302 (26)	57 (32.0)	52 (23.9)	49 (24.3)	2 (28.6)	66 (24.9)	26 (29.2)	11 (29.7)	39 (25.5)	0.65
Haemodynamics										
SBP (mmHg)	162.1 (25.1)	159.38 (25.4)	164.4 (27.4)	163.9 (26.2)	166.4 (27.7)	161.3 (25.0)	162.8 (26.3)	167.7 (28.4)	162.2 (26.8)	0.49
DBP (mmHg)	91.6 (17.9)	90. (21.1)	93.7 (19.4)	92.1 (20.4)	93.0 (12.4)	91.2 (17.9)	92.5 (17.7)	90.5 (24.4)	91.6 (18.6)	0.68
HR (bpm)	82.2 (18.6)	81.8 (18.3)	83.3 (18.9)	81.9 (20.1)	79.1 (18.0)	82.6 (22.2)	82.4 (18.3)	91.9 (26.7)	82.2 (18.1)	0.21
Temperature	36.4 (0.6)	36.6 (0.6)	36.5 (0.6)	36.5 (0.6)	36.5 (0.6)	36.6 (0.6)	36.5 (0.7)	36.6 (0.6)	36.3 (0.7)	0.012
Glucose (mmol/l)	7.5 (3.3)	7.5 (3.0)	7.1 (2.6)	7.8 (4.2)	5.8 (1.0)	8.1 (3.3)	7.0 (2.3)	6.9 (3.0)	7.7 (4.0)	0.18
Diagnosis (%)										0.05
ICH	145 (12.6)	23 (12.9)	25 (11.5)	19 (9.4)	1 (14.3)	39 (14.7)	9 (10.1)	4 (10.8)	25 (16.3)	
Ischaemic	597 (52.0)	89 (50.0)	122 (56.0)	89 (44.1)	5 (71.4)	141 (53.2)	50 (56.2)	20 (54.1)	81 (52.9)	
TIA	109 (9.5)	14 (7.9)	21 (9.6)	21 (10.4)	0 (0.0)	28 (10.6)	12 (13.5)	2 (5.4)	11 (7.2)	
Mimic	298 (25.9)	52 (29.2)	50 (22.9)	73 (36.1)	1 (14.3)	57 (21.5)	18 (20.2)	11 (29.7)	36 (23.5)	

† Protocol violation

SBP: systolic blood pressure; DBP: diastolic blood pressure; HR: heart rate; bpm: beats per minute; E&W: England & Wales; EEAS – East of England Ambulance Service NHS Trust; EMAS – East Midlands Ambulance Service NHS Trust; FAST: Face arm speech test; GCS: Glasgow coma scale; ICH: intracerebral haemorrhage; LAS – London Ambulance Service; mRS: modified Rankin Scale (premorbid); OTR: onset to randomisation; SCAS – South Central Ambulance Service NHS Foundation Trust; SWAS – South Western Ambulance Service NHS Foundation Trust; TIA: transient ischaemic attack; WAS – Welsh Ambulance Service NHS Trust; YAS – Yorkshire Ambulance Service NHS Trust.

Multiple comparison procedure: Temperature - YAS differs from SWAS (p=0.002) and EEAS (p=0.002)

For peer review only - http://bmjopen.bmj.com/site/about/guidelines.xhtml

SUPPLEMENTAL TABLE III. Univariate correlations between baseline characteristics for age, sex, heart rate, systolic blood pressure and time from onset of symptoms to randomisation. Data are Spearman or point-biserial correlation coefficient (p-value).

	Sex	HR	SBP	Glucose	OTR	FAST	GCS
Age	-0.123 (<0.001)	-0.111 (<0.001)	0.076 (0.010)	0.140 (<0.001)	0.072 (0.15)	-0.004 (0.90)	-0.158 (<0.001)
Sex		-0.094 (0.001)	-0.069 (0.019)	-0.009 (0.76)	0.037 (0.21)	0.002 (0.95)	0.009 (0.76)
HR			0.119 (<0.001)	0.069 (0.019)	-0.012 (0.67)	0.007 (0.80)	0.006 (0.85)
SBP				0.26 (1.00)	0.005 (0.86)	-0.075 (0.011)	0.044 (0.14)
Glucose					0.023 (0.44)	0.059 (0.45)	-0.77 (0.009)
OTR						-0.137 (<0.001)	0.102 (0.001)
FAST							-0.157 (<0.001)

HR: heart rate; OTR: onset to randomisation; SBP: systolic blood pressure

For peer review only

SUPPLEMENTAL TABLE IV. Timings (in minutes) by ambulance service

Data are median [interquartile range] (minimum-maximum). Comparisons by Kruskal-Wallis and multiple comparison procedures (between groups) using Dunn's test with Bonferroni correction.

		E&W	EEAS	EMAS	LAS	SCAS	SWAS	WAS	WMAS	YAS	2p
Patients (N)		1149	178	218	202	7	265	89	37	153	-
Onset †	emergency Call	19 [5, 64] (-216, 920)	18 [6, 69] (-58, 183)	15 [4, 57] (-89, 661)	25 [7, 76] (7, 76)	24 [11, 48] (1, 776)	18 [4, 55] (-216, 835)	14 [4, 46] (-45, 199)	27 [7, 75] (-25, 206)	20 [8, 65] (-10, 514)	0.36
emergency Call	Dispatch	3 [1, 7] (0, 158)	1 [0, 4] (0, 115)	2 [1, 4] (0, 73)	4 [2, 8] (0, 78)	0 [0, 1] (0, 3)	5 [3, 14] (0, 131)	1 [0, 11] (0, 158)	2 [1, 3] (0,17)	2 [1, 7] (0, 55)	<0.001
Onset †	Arrive Scene (R1)	40 [21, 84] (-124, 928)	39 [19, 85] (-40, 205)	30 [15, 74] (-75, 691)	45 [19, 95] (-21, 928)	35 [33, 56] (11, 792)	45 [25, 79] (-124, 838)	28 [25, 85] (-19, 207)	39 [20, 95] (-14, 214)	40 [24, 84] (5, 523)	0.04
Onset †	Arrive Scene (R2)	44 [23, 86] (-124, 928)	43 [21, 88] (-40, 206)	35 [19, 80] (-75, 691)	50 [22, 97] (-75, 928)	35 [33, 56] (11, 792)	58 [25, 83] (-124, 838)	40 [25, 87] (-17, 207)	39 [20, 95] (-14, 214)	42 [25, 92] (5, 423)	0.23
Dispatch	Arrive Scene (R1)	8 [5, 13] (-31, 61)	8 [4, 14] (-26, 61)	7 [4, 12] (-31, 36)	6 [4, 9] (0, 28)	11 [7, 15] (6, 22)	10 [5, 17] (-18, 48)	12 [6, 17] (-9, 40)	9 [5, 13] (0, 39)	9 [6, 23] (-31, 39)	<0.001
Dispatch	RIGHT-2 paramedic	10 [6, 16] (0, 75)	10 [6, 19] (1, 61)	8 [5, 15] (0, 53)	8 [5, 13] (0, 65)	11 [7, 15] (6, 22)	11 [6, 18] (0, 75)	12 [7, 18] (0, 72)	9 [6, 14] (0, 39)	9 [6, 13] (-31, 39)	<0.001
Onset	Randomisation	71 [45, 116] (4, 942)	73 [47, 120] (11, 250)	59 [35, 100] (4, 720)	77 [51, 124] (15, 942)	53 [45, 65] (19, 811)	75 [49, 107] (6, 850)	75 [48, 123] (11, 395)	60 [32, 115] (17, 225)	70 [45, 118] (15, 535)	<0.001
Onset	Treatment	72 [48, 117] (4, 942)	73 [49, 73] (11, 251)	60 [37, 104] (4, 720)	77 [52, 124] (15, 942)	59 [50, 75] (19, 816)	78 [50, 109] (6, 874)	77 [48, 120] (11, 230)	65 [35, 118] (22, 229)	71 [48, 119] (22, 535)	0.005
RIGHT-2 Paramedic arrival †	Consent	19 [12, 29] (-17, 128)	20 [15, 28] (-1, 91)	15 [9, 22] (-5, 76)	25 [18, 33] (1, 73)	8 [5, 18] (2, 29)	20 [13, 30] (-10, 128)	23 [14, 32] (0, 87)	16 [9, 21] (-9, 46)	17 [10, 25] (-17, 65)	<0.001
RIGHT-2 Paramedic arrival †	Randomisation	22 [15, 31] (-34, 130)	23 [18, 32] (1, 95)	17 [11, 27] (-34, 80)	26 [19, 33] (1, 73)	11 [8, 19] (5, 30)	22 [16, 32] (-10, 130)	25 [18, 35] (0, 87)	17 [12, 24] (-8, 46)	19 [12, 27] (-13, 71)	<0.001
Consent	Randomisation	1 [0, 4] (0, 30)	2 [0, 4] (0, 30)	1 [0, 4] (0, 24)	0 [0, 0] (0, 18)	1 [0, 5] (0, 9)	0 [0, 4] (0, 20)	2 [0, 5] (0, 29)	1 [0, 3] (0, 10)	1 [0, 4] (0, 23)	<0.001
Arrive Scene	Depart scene	33 [26, 46] (0, 224)	38 [29, 49] (15, 224)	33 [25, 44] (10, 94)	31 [25, 38] (8, 72)	29 [23, 40] (10, 41)	43 [34, 87] (0, 162)	38 [28, 51] (15, 114)	31 [25, 38] (16, 46)	32 [24, 43] (0, 84)	<0.001
Onset	Hospital	97 [71, 141] (26, 953)	103 [73, 149] (29, 295)	87 [65, 129] (26, 748)	95 (68, 144] (32, 953)	90 [81, 115] (55, 841)	106 [77, 141] (32, 889)	109 [78, 153] (39, 430)	86 [58, 150] (31, 256)	92 [70, 131] (38, 545)	0.008
Randomisation	Treatment	0 [0, 2] (0, 57)	0 [0, 3] (0, 20)	0 [0, 2] (0, 57)	0 [0, 0] (0, 9)	5 [0, 8] (0, 9)	1 [0,4] (0, 27)	0 [0, 2] (0, 19)	1 [0, 4] (0, 24)	0 [0, 2] (0, 22)	<0.001

For peer review only

Randomisation	Hospital	24 [16, 34] (-13, 229)	25 [18, 33] (2, 229)	26 [17, 35] (-13, 92)	14 [10, 21] (0, 52)	35 [30, 36] (25, 62)	29 [21, 39] (4, 85)	30 [19, 42] (-1, 92)	22 [19, 28] (6, 48)	23 [16, 29 (-3, 86)]	<0.001
Depart scene	Hospital	15 [10, 23] (0, 98)	17 [10, 23] (1, 47)	14 [9, 23] (0, 50)	13 [10, 19] (2, 49)	24 [14, 25] (4, 33)	20 [12, 29] (0, 98)	16 [10, 28] (0, 56)	9 [7, 13] (-3, 27)	12 [8, 17] (0, 45)	<0.001

† Negative times: paramedic already at scene

E&W: England & Wales; EEAS – East of England Ambulance Service NHS Trust; EMAS – East Midlands Ambulance Service NHS Trust; LAS – London Ambulance Service; R1: First resource; R2: RIGHT-2 trained paramedic (if not on first resource) – two ambulance services permitted single responder paramedics to participate, otherwise R2 trained paramedics arrive on double-crewed ambulances; SCAS – South Central Ambulance Service NHS Foundation Trust; SWAS – South Western Ambulance Service NHS Foundation Trust; WAS – Welsh Ambulance Service NHS Trust; YAS – Yorkshire Ambulance Service NHS Trust.

Results of multiple comparison testing are given in the Supplemental material.

For peer review only - http://bmjopen.bmj.com/site/about/guidelines.xhtml

SUPPLEMENTAL TABLE V. Timings: Onset of symptoms to emergency call. Data are N (%), median [25, 75 centile]; comparison by Kruskal-Wallis test.

	E&W	EEAS	EMAS	LAS	SCAS	SWAS	WAS	WMAS	YAS	2p
Minutes<br>N (%)<br>Median [25, 75 centile]	1149<br>19 [5, 64]	178 (15.5)<br>18 [6, 69]	218 (19.0)<br>15 [4, 57]	202 (17.6)<br>25 [7, 76]	7 (0.6)<br>24 [11, 48]	265 (23.1)<br>18 [4, 55]	89 (7.7)<br>14 [4, 46]	37 (3.2)<br>27 [7, 75]	153 (13.3)<br>20 [8, 65]	0.36
N (%)										0.012
<10	430 (37.4)	67 (37.6)	94 (43.1)	68 (33.7)	1 (14.3)	102 (38.5)	40 (44.9)	12 (32.4)	46 (30.1)	
11-20	169 (14.7)	26 (14.6)	29 (13.3)	27 (13.4)	1 (14.3)	39)14.7)	11 912.4)	4 (10.8)	32 (20.9)	
21-30	84 (7.3)	9 (5.1)	18 (8.3)	14 (6.9)	2 (28.6)	23 (8.7)	3 (3.4)	3 (8.1)	12 (7.8)	
31-60	164 (14.3)	23 (12.9)	25 (11.5)	29 (14.4)	2 (28.6)	43 (16.2)	16 (18.0)	5 (13.5)	21 (13.7)	
61-240	290 (25.2)	53 (29.8)	50 (22.9)	58 (28.7)	0 (0.0)	57 (21.5)	18 (20.2)	13 (35.1)	41 (26.8)	
>240 †	11 (1.0)	0 (0.0)	2 (0.9)	6 (3.0)	1 (14.3)	1 (0.4)	88 (98.9)	0 (0.0)	1 (0.7)	

† >240 minutes is protocol violation, typically due to wake-up stroke or uncertainty of onset time.

For peer review only

SUPPLEMENTAL TABLE VI. Comparison of EMAS stroke patients in RIGHT-2 versus non-RIGHT-2. Data are median of time (min) [25, 75 centile] or distance km [25, 75 centile]

	RIGHT-2	Non-RIGHT-2	2p
Patients (N)	218	49	
Time			
Symptom onset – emergency call	15 [5, 57]	†	
emergency call – dispatch	2 [1, 5]	2 [1, 3]	0.48
emergency call – scene arrival	10 [7, 16]	12 [8, 18]	0.17
Time on scene	34 [26, 44]	32 [23, 41]	0.12
Scene arrival - hospital			
Scene departure – hospital	14 [9, 22]	17 [12, 25]	0.18
Symptom onset – hospital	86 [65, 128]	†	
emergency call - hospital	63 [48, 76]	62 [49, 82]	0.80
Distance			
Dispatch - Scene	7.3 [3.5, 12.0]	9.6 [3.6, 16.7]	0.23
Scene - hospital	10.0 [0.4, 64.7]	15.9 [7.6, 24]	0.011

† Symptom onset time not available

For peer review only

SUPPLEMENTAL TABLE VII. Timings: Symptom onset to arrival at hospital (minutes). Data are N (%), median [25, 75 centile]; comparison by Kruskal-Wallis test.

Minutes	E&W	EEAS	EMAS	LAS	SCAS	SWAS	WAS	WMAS	YAS	2p
N (%)	1149	178	218	202	7	265	89	37	153	
Median [25, 75 centile]	97 [71, 141]	103 [73, 149]	87 [65, 129]	95 (68, 144]	90 [81, 115]	106 [77, 141]	109 [78, 153]	86 [58, 150]	92 [70, 131]	0.008
N(%)										0.040
<30	6 (0.5)	1 (0.6)	5 (2.3)	0 (0.0)	0 (0.0)	0 (0.0)	0 (0.0)	0 (0.0)	0 (0.0)	
31-60	157 (13.7)	15 (8.4)	39 (17.9)	37 (18.3)	1 (14.3)	24 (9.1)	9 (10.1)	10 (27.0)	22 (14.4)	
61-90	350 (30.5)	61 (34.3)	70 (32.1)	54 (26.7)	3 (42.9)	75 (28.3)	23 (25.8)	10 (27.0)	54 (35.3)	
91-120	227 (19.8)	29 (16.3)	40 (18.3)	39 (19.3)	2 (28.6)	62 (23.4)	20 (22.5)	5 (13.5)	30 (19.6)	
121-240	371 (32.3)	68 (38.2)	58 (26.6)	64 (31.7)	0 (0.0)	96 (36.2)	34 (38.2)	11 (29.7)	40 (26.1)	
>240	38 (3.3)	4 (2.2)	6 (2.8)	8 (4.0)	1 (14.3)	8 (3.0)	3 (3.4)	1 (2.7)	7 (4.6)	

For peer review only

SUPPLEMENTAL TABLE VIII. Conveyance distances (kilometres). Data are median of distance (minimum-maximum). Comparison by Kruskal-Wallis test and multiple comparison procedure. One ambulance service was unable to provide location data.

	E&W	EEAS	EMAS	SCAS	SWAS	WAS	WMAS	YAS	2p
N (%)	936	178	213	7	263	87	37	152	
Median	10.0	12.3	9.4	19.9	13.6	12.1	4.1	6.4	<0.001
(min, max)	[4.4, 18.4]	[4.5, 20.9]	[4.4, 19.1]	[2.7, 19.8]	[6.2, 20.1]	[4.7, 23.8]	[3.4, 10.2]	[3.3, 10.8]	
	(0.4, 64.7)	(0.6, 34.0)	(1.1, 59.9)	(1.9, 22.4)	(0.6, 51.3)	(0.6, 64.7)	(0.9, 28.8)	(0.4, 44.3)	
N (%)									<0.001
<5 Km	273 (29.2)	49 (27.7)	66 (31.0)	2 (28.6)	49 (18.6)	20 (23.2)	23 (62.2)	64 (42.1)	
5-10 km	193 (20.6)	25 (14.1)	42 (19.7)	0 (0.0)	61 (23.2)	18 (20.7)	4 (10.8)	43 (28.3)	
10.1-15 km	142 (15.2)	27 (15.3)	31 (14.6)	1 (14.3)	41 (15.6)	13 (14.9)	5 (13.5)	24 (15.8)	
15.1-20 km	112 (12.0)	22 (12.4)	26 (12.2)	1 (14.2)	44 (16.7)	10 (11.5)	3 (8.1)	6 (3.9)	
20.1-25 km	107 (11.4)	34 (19.2)	25 (11.7)	3 (42.9)	28 (10.6)	10 (11.5)	1 (2.7)	6 (3.9)	
>=25 km	109 (11.6)	20 (11.3)	23 (10.8)	0 (0.0)	40 (15.2)	16 (18.4)	1 (2.7)	9 (5.9)	

Multiple Comparison testing:
WMAS differs from EMAS p=0.016, EEAS p=0.001, SWAS p<0.001 and WAS p=0.001
YAS differs from EMAS p=0.002, EEAST p<0.001, SWAS p<0.001 and WAS p<0.001

For peer review only

SUPPLEMENTAL FIGURE I. Distribution of participants by Ambulance Service

Map pins indicate location of participants recruited

A) East Midlands Ambulance Service NHS Trust

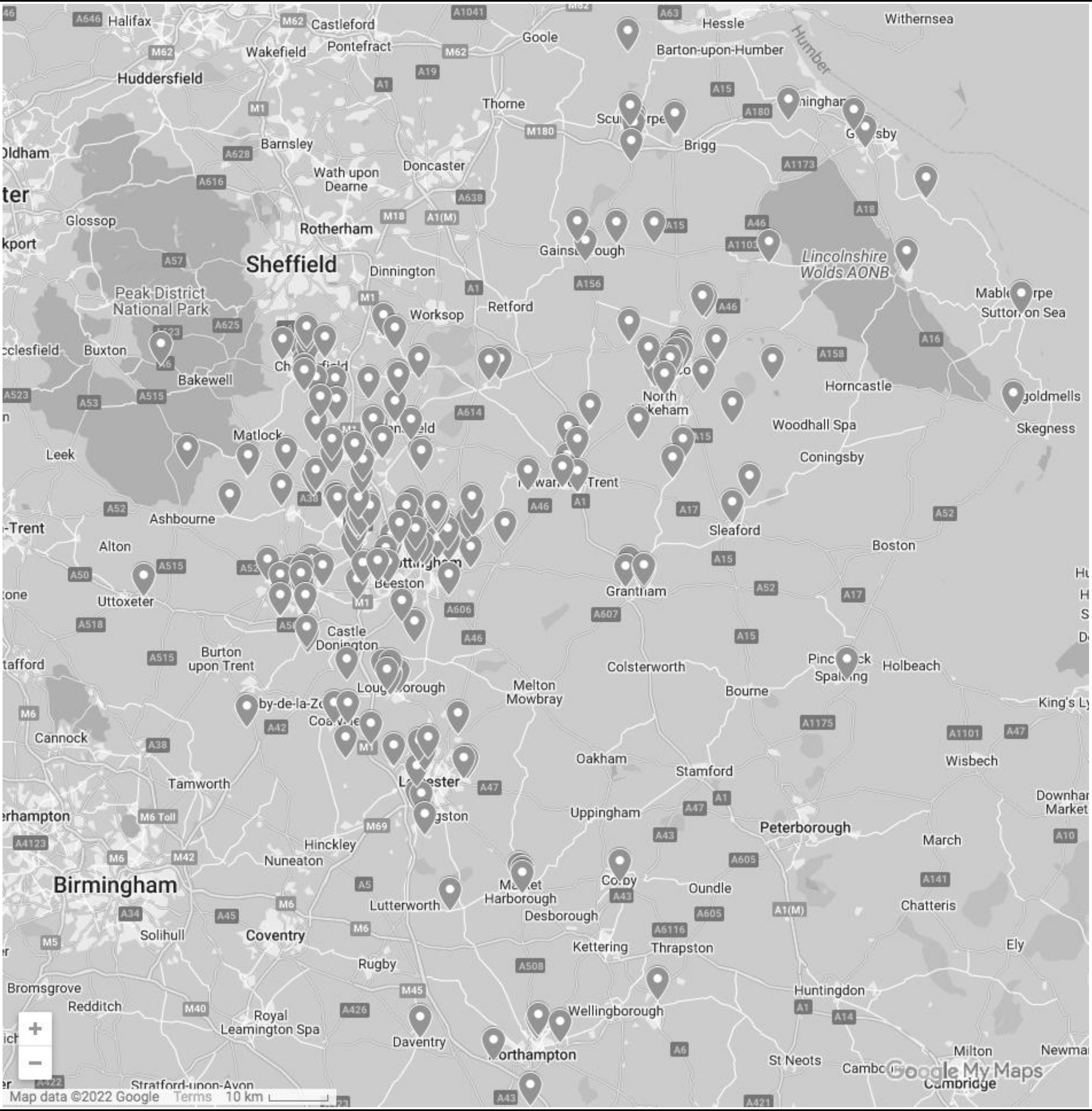

B) East of England Ambulance Service NHS Trust

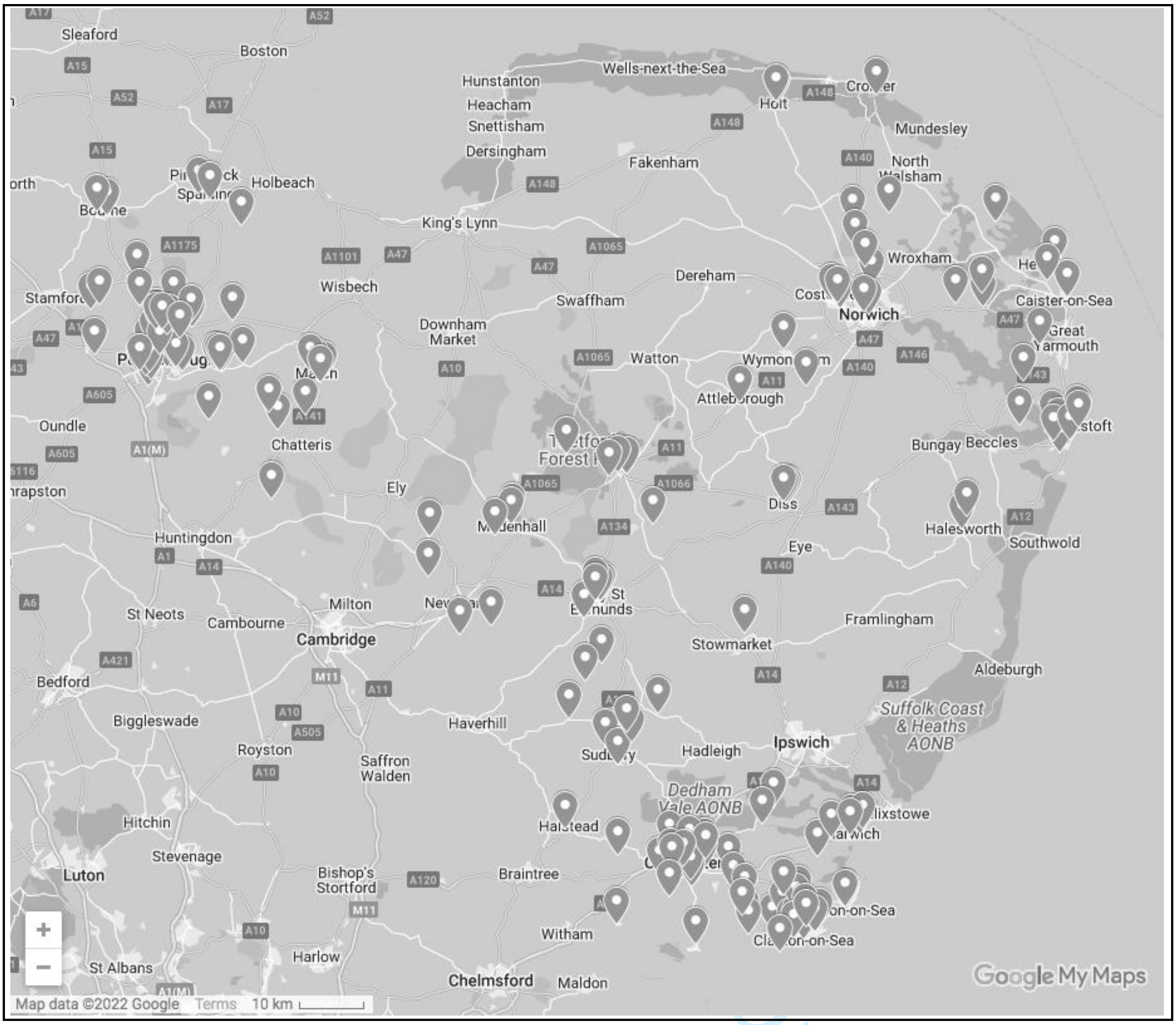

For peer review only - http://bmjopen.bmj.com/site/about/guidelines.xhtml

C) South Central Ambulance Service NHS Foundation Trust

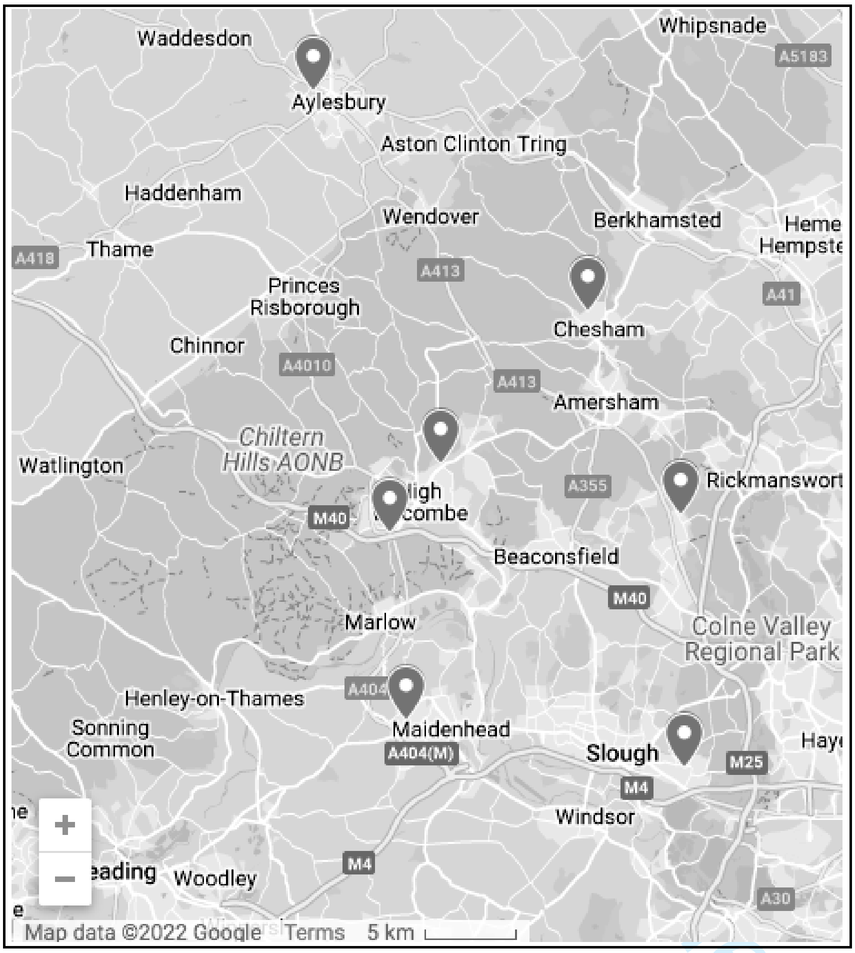

D) South Western Ambulance Service NHS Foundation Trust

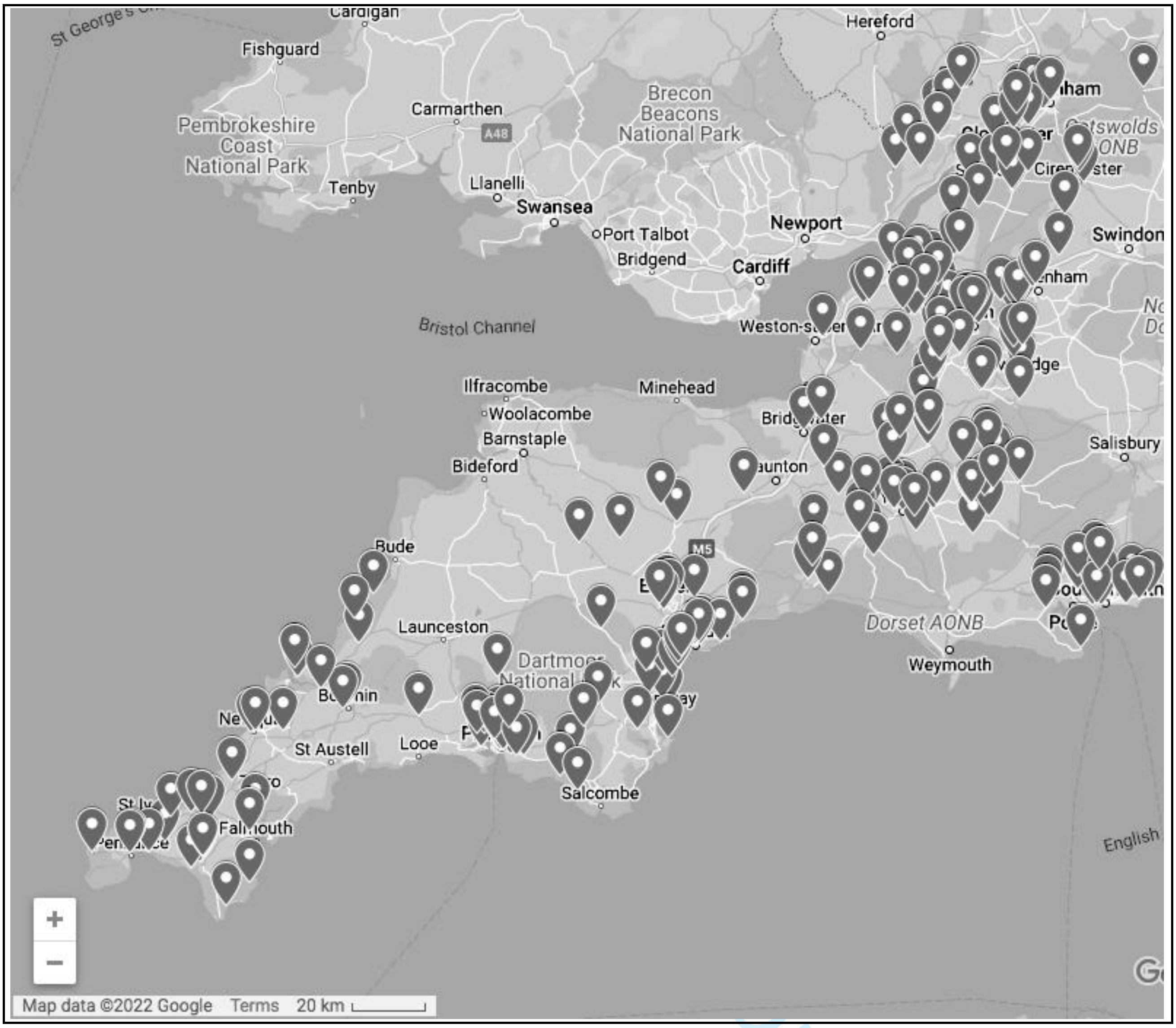

E) Welsh Ambulance Service NHS Trust

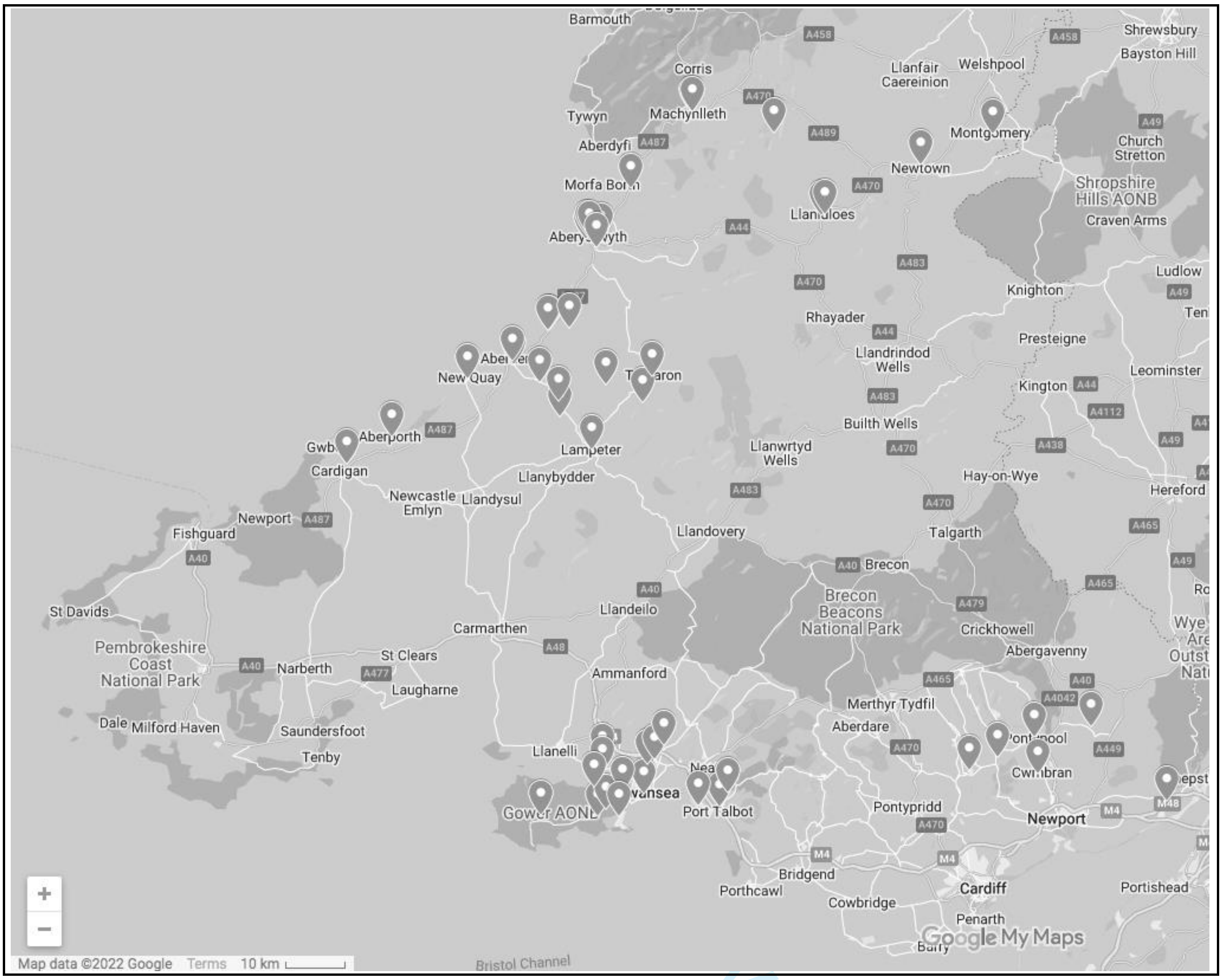

F) West Midlands Ambulance Service University NHS Foundation Trust

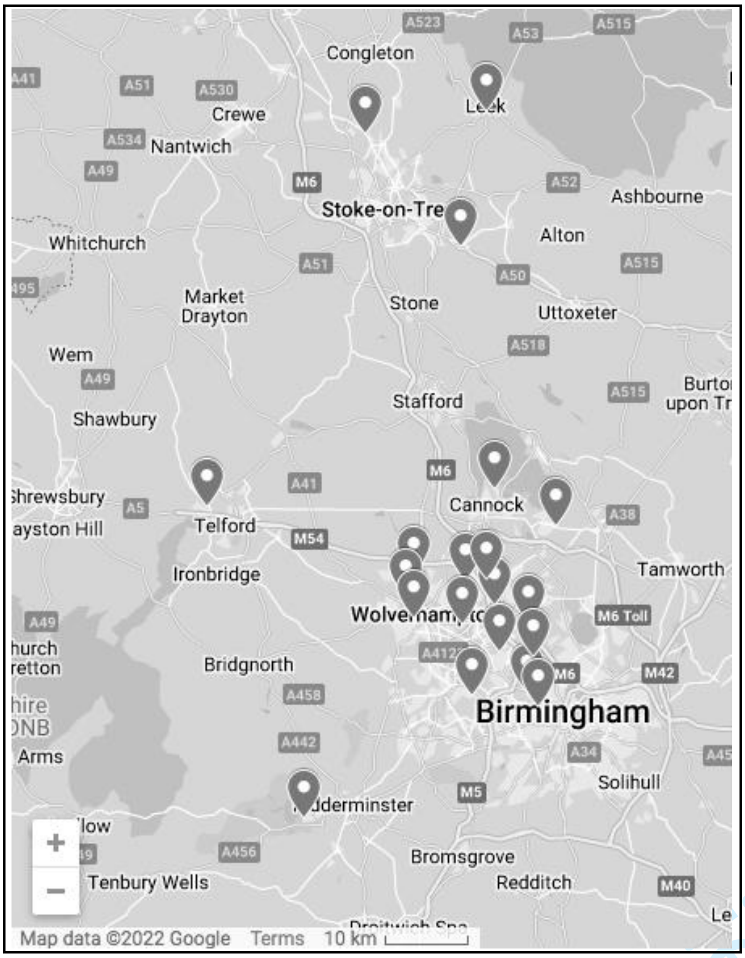

For peer review only

G) Yorkshire Ambulance Service NHS Trust

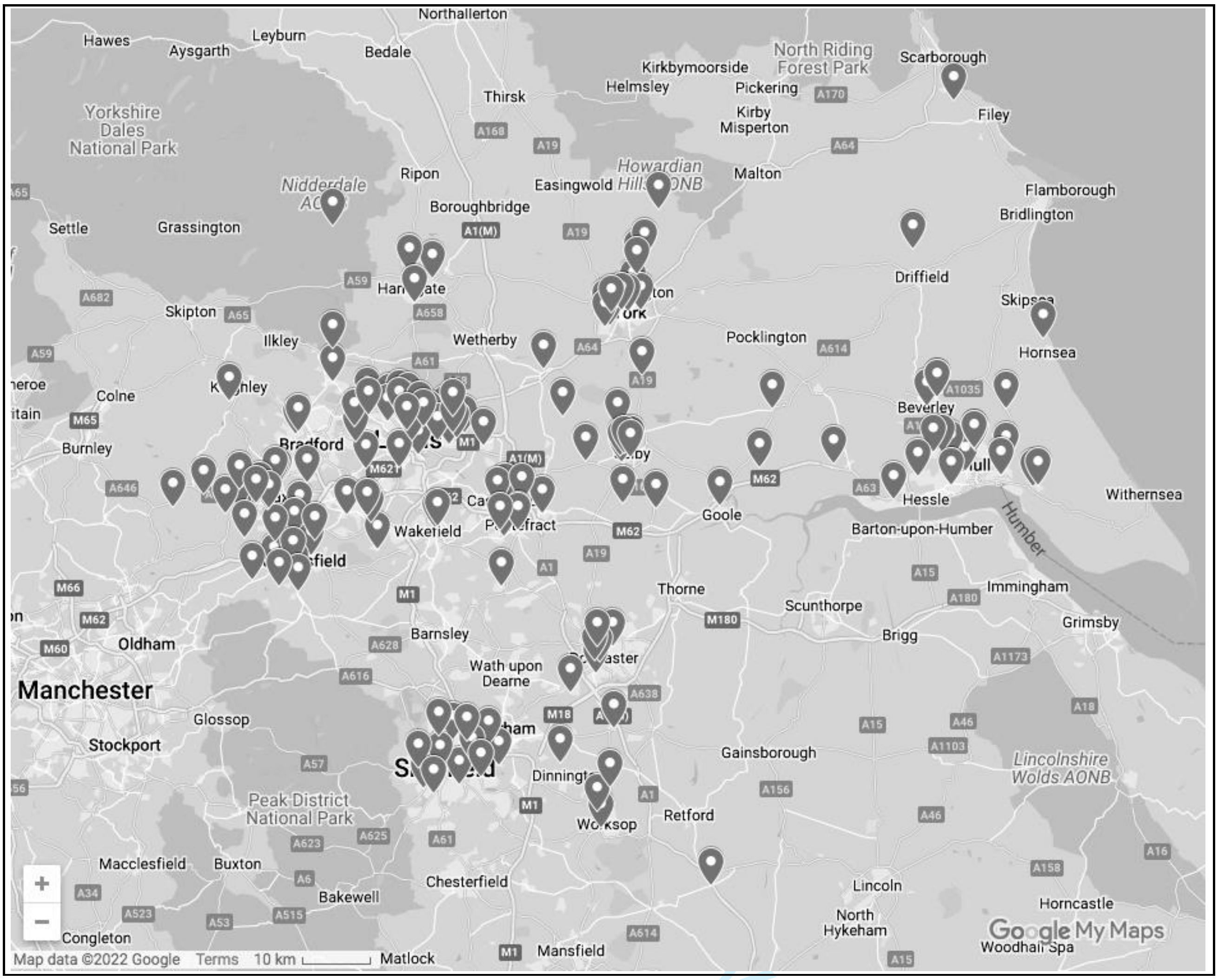

For peer review only - http://bmjopen.bmj.com/site/about/guidelines.xhtml

SUPPLEMENTAL REFERENCES

1. Office for National Statistics. 2011 Census Analysis - Comparing Rural and Urban Areas of England and Wales: Office for National Statistics, 2013.
2. Appleton JP, Scutt P, Dixon M, et al. Ambulance-delivered transdermal glyceryl trinitrate versus sham for ultra-acute stroke: Rationale, design and protocol for the Rapid Intervention with Glyceryl trinitrate in Hypertensive stroke Trial-2 (RIGHT-2) trial (ISRCTN26986053). *Int J Stroke* 2017;0(0):1-16. doi: 10.1177/1747493017724627

For peer review only

For peer review only - http://bmjopen.bmj.com/site/about/guidelines.xhtml

CONSORT 2010 checklist of information to include when reporting a randomised trial*

Section/Topic	Item No	Checklist item	Reported on page No
**Title and abstract**			
	1a	Identification as a randomised trial in the title	1
	1b	Structured summary of trial design, methods, results, and conclusions (for specific guidance see CONSORT for abstracts)	5
**Introduction**			
Background and objectives	2a	Scientific background and explanation of rationale	4
	2b	Specific objectives or hypotheses	4
**Methods**			
Trial design	3a	Description of trial design (such as parallel, factorial) including allocation ratio	5
	3b	Important changes to methods after trial commencement (such as eligibility criteria), with reasons	
Participants	4a	Eligibility criteria for participants	5
	4b	Settings and locations where the data were collected	5
Interventions	5	The interventions for each group with sufficient details to allow replication, including how and when they were actually administered	5
Outcomes	6a	Completely defined pre-specified primary and secondary outcome measures, including how and when they were assessed	5/6
	6b	Any changes to trial outcomes after the trial commenced, with reasons	n/a
Sample size	7a	How sample size was determined	5 (refers to previous protocol publication)
	7b	When applicable, explanation of any interim analyses and stopping guidelines	5 (refers to previous protocol publication)
Randomisation:			
Sequence generation	8a	Method used to generate the random allocation sequence	5 (refers to previous

For peer review only - http://bmjopen.bmj.com/site/about/guidelines.xhtml

			protocol publication)
	8b	Type of randomisation; details of any restriction (such as blocking and block size)	5 (refers to previous protocol publication)
Allocation concealment mechanism	9	Mechanism used to implement the random allocation sequence (such as sequentially numbered containers), describing any steps taken to conceal the sequence until interventions were assigned	5 (refers to previous protocol publication)
Implementation	10	Who generated the random allocation sequence, who enrolled participants, and who assigned participants to interventions	5 (refers to previous protocol publication)
Blinding	11a	If done, who was blinded after assignment to interventions (for example, participants, care providers, those assessing outcomes) and how	5 (refers to previous protocol publication)
	11b	If relevant, description of the similarity of interventions	5 (refers to previous protocol publication)
Statistical methods	12a	Statistical methods used to compare groups for primary and secondary outcomes	5 / 6
	12b	Methods for additional analyses, such as subgroup analyses and adjusted analyses	6
**Results**			
Participant flow (a diagram is strongly recommended)	13a	For each group, the numbers of participants who were randomly assigned, received intended treatment, and were analysed for the primary outcome	8
	13b	For each group, losses and exclusions after randomisation, together with reasons	n/a
Recruitment	14a	Dates defining the periods of recruitment and follow-up	8
	14b	Why the trial ended or was stopped	Available in main RIGHT-2 results paper

For peer review only

For peer review only - http://bmjopen.bmj.com/site/about/guidelines.xhtml

			(Lancet, 2019)
Baseline data	15	A table showing baseline demographic and clinical characteristics for each group	22
Numbers analysed	16	For each group, number of participants (denominator) included in each analysis and whether the analysis was by original assigned groups	Available in main RIGHT-2 results paper (Lancet, 2019)
Outcomes and estimation	17a	For each primary and secondary outcome, results for each group, and the estimated effect size and its precision (such as 95% confidence interval)	Available in main RIGHT-2 results paper (Lancet, 2019)
	17b	For binary outcomes, presentation of both absolute and relative effect sizes is recommended	Available in main RIGHT-2 results paper (Lancet, 2019)
Ancillary analyses	18	Results of any other analyses performed, including subgroup analyses and adjusted analyses, distinguishing pre-specified from exploratory	8 / 9
Harms	19	All important harms or unintended effects in each group (for specific guidance see CONSORT for harms)	Available in main RIGHT-2 results paper (Lancet, 2019)
**Discussion**			
Limitations	20	Trial limitations, addressing sources of potential bias, imprecision, and, if relevant, multiplicity of analyses	11
Generalisability	21	Generalisability (external validity, applicability) of the trial findings	12
Interpretation	22	Interpretation consistent with results, balancing benefits and harms, and considering other relevant evidence	10 / 11

For peer review only - http://bmjopen.bmj.com/site/about/guidelines.xhtml

**Other information**			
Registration	23	Registration number and name of trial registry	3
Protocol	24	Where the full trial protocol can be accessed, if available	5
Funding	25	Sources of funding and other support (such as supply of drugs), role of funders	3 / 13

*We strongly recommend reading this statement in conjunction with the CONSORT 2010 Explanation and Elaboration for important clarifications on all the items. If relevant, we also recommend reading CONSORT extensions for cluster randomised trials, non-inferiority and equivalence trials, non-pharmacological treatments, herbal interventions, and pragmatic trials. Additional extensions are forthcoming: for those and for up to date references relevant to this checklist, see www.consort-statement.org.

For peer review only

For peer review only - http://bmjopen.bmj.com/site/about/guidelines.xhtml
